# Diosgenin From Dioscorea Nipponica Rhizoma Against Graves’ Disease—On Network Pharmacology and Experimental Evaluation

**DOI:** 10.3389/fphar.2021.806829

**Published:** 2022-01-24

**Authors:** Jingxin Xin, Wencong Cheng, Yongbing Yu, Juan Chen, Xinhuan Zhang, Shanshan Shao

**Affiliations:** ^1^ Department of Endocrinology, Shandong Provincial Hospital Affiliated to Shandong First Medical University, Jinan, China; ^2^ Department of Endocrinology, The Second Affiliated Hispital of Shandong First Medical University, Taian, China; ^3^ Shandong Clinical Research Center of Diabetes and Metabolic Diseases, Jinan, China; ^4^ Shandong Key Laboratory of Endocrinology and Lipid Metabolism, Jinan, China

**Keywords:** dioscorea nipponica rhizoma, diosgenin, graves’ disease, network pharmacology, IGF-1R, apoptosis, traditional chinese medicine

## Abstract

Dioscorea nipponica rhizoma (DNR) is commonly used for the cure of hyperthyroidism resulting from Graves’ disease (GD) or thyroid nodules. However, its therapeutic mechanism remains unclear. This study aimed to utilize network pharmacology integrated molecular docking and experimental verification to reveal the potential pharmacological mechanism of DNR against GD. First, the active componds of DNR were collected from the HERB database and a literature search was conducted. Then, according to multisource database, the predicted genes of DNR and GD were collected to generate networks. The analysis of protein–protein interaction and GO enrichment and KEGG pathway were employed to discover main mechanisms associated with therapeutic targets. Moreover, molecular docking simulation was applied in order to verify the interactions between the drug and target. Finally, our experiments validated the ameliorated effects of diosgenin, the main component of DNR, in terms of phosphorylation deactivation in IGF-1R, which in turn inhibited the phosphorylation and activation of PI3K-AKT and Rap1-MEK signaling pathways, promoting cell apoptosis and GD remission. Our present study provided a foundation for further investigation of the in-depth mechanisms of diosgenin in GD and will provide new scientific evidence for clinical application.

## 1 Introduction

Graves’ disease (GD) is an organ-specific autoimmune disease featured by hyperthyroidism, diffuse goiter and thyroid-associated ophthalmopathy (Bahn et al., 2011). Hyperthyroidism of GD is due to the liganding of thyrotropin receptor (TSHR) on thyroid cells by stimulatory autoantibodies, which act as a TSHR agonist and induce excessive secretion of thyroid hormones, causing the thyroid to escape the control of the pituitary gland (Ross et al., 2016). GD is the most common cause of hyperthyroidism in iodine-sufficient regions, with 20–30 GD patients per 100,000 people per year (Kahaly et al., 2018). Female patients are reported to be more likely to develop GD, with a population prevalence of 1–1.5% (Nyström et al., 2013). The primary role of currently used drugs for GD is to inhibit thyroid hormones synthesis by interfering with thyroid peroxidase expression rather than promoting apoptosis or inhibiting excessive proliferation of thyrocytes (Cooper, 2005). Therefore, these antithyroid drugs cannot effectively alleviate goiter, resulting in a heavy financial burden and mental stress on GD patients with large goiters. Proapoptotic and antiproliferative approaches might be potential therapies for the treatment of GD goiter.

According to traditional Chinese medicine (TCM) theory, Dioscorea nipponica rhizoma (DNR), the rhizome of Dioscorea nipponica Makino (DNM), is rich in a variety of steroidal saponins. DNR exhibits various pharmacological activities, such as anti-inflammatory (Yao et al., 2012), analgesic (Vasanthi et al., 2010), antitumor (X. Li et al., 2017) hypoglycemic (Tang et al., 2015), and hypolipidemic (Vasanthi et al., 2012) activities. In the treatment of thyroid disease, DNR is used for thyroid tumors and hyperthyroidism. Wang et al. used the DNM to treat established GD rat model and concluded that DNM could inhibit the process of iodine capture and improve the morphological structure disorder of thyroid in GD (Q. H. Wang and Chen, 2007). However, it was reported that diosgenin produced from the metabolism of intestinal flora is the major bioactive compound and plays a vital role after the oral administration of dioscin (Ou-Yang et al., 2018). Its main component, diosgenin (Yan et al., 2013; Yi et al., 2014), has also been reported to inhibit proliferation and cell progress (Bian et al., 2011), promote apoptosis (Mu et al., 2012) of thyrocytes, and relieve goiter (Cai et al., 2014). Nevertheless, its pharmacodynamic mechanism remains unclear.

With the widely application of bioinformatics, network pharmacology has emerged as effective tool toward TCM research (Hao and Xiao, 2014). Based on omics data analysis, high-performance virtual computing and network database retrieval, network pharmacology could not only build the priority of disease-associated genes, but also predict the target information and pharmacological effects of TCM compounds systematically (Hopkins, 2008; S.; Li and Zhang, 2013; T. T.; Luo et al., 2020). It is believed to a new original discipline of cost-effective drug development in the era of artificial intelligence and Big Data (S. Li, 2021). In the current results, we adopted a network pharmacology approach coupled with molecular docking to screen the putative targets and signaling pathways of DNR against GD and then conducted experimental verification on a rat model of GD goiter to further illustrate the pharmacological mechanism of DNR against GD.

## 2 Materials and Methods

### 2.1 Database-Based Network Pharmacology Analysis

#### 2.1.1 Screening of the Active Chemical Constituents of DNR

To collect the pharmacologically active ingredients of DNR, the term “DNR and DN” was searched first in the HERB database (http://herb.ac.cn/) (Fang et al., 2021). The database links 12,933 targets and 28,212 diseases with 7,263 Chinese herbal medicines and 49,258 Chinese medicine ingredients, providing six pairing relationships between them. The ingredients of DNR searched from HERB are mainly from the TCMSP, SymMap, TCMID, and TCM-ID databases. Then, the BATMAN database (http://bionet.ncpsd.org/batman-tcm) (Z. Liu et al., 2016), domestic as well as foreign literature (Guo et al., 2016; Lin et al., 2007) supplemented the search. According to the absorption, distribution, metabolism, excretion, and toxicity characteristics of ADME (Daina et al., 2017) (http://www.swissadme.ch/), the Lipinski rules (Omran and Rauch, 2014) were used to screen the active ingredients of DNR. It has four rules: MW ≤ 500; nON≤10; nOHNH≤5; MLogP≤4.15. A compound that met at least two conditions was determined to be an orally active drug. If the compound in the database does not meet the Lipinski rules, it can also be included if there is a compound in relevant reports clearly described as the active ingredient of DNR (https://pubchem.ncbi.nlm.nih.gov/).

### 2.2 Prediction and Screening of the Related Targets of DNR

To prepare for predicting the target of the active ingredient, the 2D structural formulas of the active ingredients were downloaded from PubChem database (Kim et al., 2021). Using the method of matching reversed pharmacophores from the Pharm Mapper database (http://www.lilab-ecust.cn/pharmmapper/) (X. Wang et al., 2016; X. Wang et al., 2017), we can predict the targets of each compound. The advantage of this method is that it uses active small molecules as probes to explore qualified putative targets and then predicts the biological activity of the compound. Then, we screened the human target genes of each component according to the score value. The Swiss Target Prediction database (http://www.swisstargetprediction.ch/) (Daina et al., 2019) is another way to predict the target. This method is based on the similarity of ligand 2D and 3D structures. The credibility of the composites was generally poor using this approach. Therefore, we added target genes with a credibility of one to the corresponding compound. After screening, we standardized the protein target information using the UniProt protein database (https://www.uniprot.org/) (UniProt, 2019).

### 2.3 Screening the Related Targets for GD

Using “Graves’ disease” as the key word, we obtained a large number of targets from the Gene Cards (Stelzer et al., 2016) (https://www.genecards.org), DisGeNET (Pinero et al., 2020) (http://www.disgenet.org/home/), CTD (Davis et al., 2021) (ctdbase.org), and GAD (Becker et al., 2004) (https://geneticassociationdb.nih.gov/) databases. Then, the therapeutic target of clinical first-line western medicine of GD was identified through the OMIM (Boyadjiev and Jabs, 2000) (http://www.omim.org), TTD (Y. Wang et al., 2020) (http://db.idrblab.net/ttd/), and DRUGBANK (Wishart et al., 2008) (https://www.drugbank.ca) (https://www.drugbank.ca) databases. In database analysis, the criteria for screening targets have little difference in databases. Briefly, they are generally judged by score value. If the target has a high score value, it indicates that the target is highly correlated to the disease. To be specific, the targets from the CTD and Gene Card databases were selected by median screening of the score value. The targets from the DisGeNET database were selected according to score values greater than the average value. In the OMIM database, the gene targets were selected with * in front of the number. After merging the targets obtained from the seven disease databases and deleting the duplicate GD target, the screened targets of disease were collected.

### 2.4 The Mapping Relation of Overlapping Targets of DNR in GD

To clarify the interaction between the predicted targets of DNR and the targets of GD, a Venn diagram was drawn with the Venn diagram website (http://www.bioinformatics.com.cn/plot_basic_proportional_2_or_3_venn_diagram_028) (W. Luo and Brouwer, 2013) to identify the intersection of the two targets. Then, the intersection target was submitted to the STRING database (https://string-db.org/) (Szklarczyk et al., 2017). To generate the protein interaction network, the organism type was set to “Homo Sapiens”, the minimum interaction threshold was set to “highest confidence” (>0.4), and the other settings were the default settings. Topological property analysis was carried out to identify important targets of diosgenin in GD.

### 2.5 GO and KEGG Analyses

To further elucidate the potential pharmacological mechanism of DNR against GD, we put common targets on DAVID 6.8 (https://david.ncifcrf.gov/) (Huang da et al., 2009) to perform GO biological process (BP), molecular function (MF), cell component (CC) and KEGG pathway analysis (Shi et al., 2017) The items with correction *p* ≤ 0.01, the top ten pathways with the most enriched targets were selected to visualize the data by bioinformatics (W. Luo and Brouwer, 2013) (http://www.bioinformatics.com.cn/). The information enrichments genes were analyzed using R-Studio.

### 2.6 Network Construction Analyses

To understand the interaction relationship of each target more intuitively, the targets were imported into the String database (https://string-db.org/cgi/input.pl) to construct a PPI network. Then, the PPI network was imported into Cytoscape 3.7.2 to perform network analysis. The following networks were constructed: 1) DNR component target network; 2) The target network of GD; 3) PPI network of DNR’s compound-GD targets; and 4) compound-core target-pathway network. The nodes in each network represent interacting molecules. An edge refers to the line connecting two nodes, which represents the interaction between nodes. Usually, three indicators are used to evaluate the topological properties of each node. The first is “Degree”, which means the number of directly connected nodes of one node in a network, and its level is proportional to the betweenness centrality of this node. This means that the more pathways that depend on this node, the more important this node is. The second is “betweenness centrality”, which means the proportion of nodes that as a shortest route in a network. The more times a node acts as an “intermediary”, the greater its degree of intermediary centrality. The third is “closeness centrality”, which means the average length of the shortest path between each two nodes. In other words, for a node, the shorter distance it is to other nodes, the higher its degree of closeness centrality. In short, if these three parameters have higher score values, the node is more important in this network.

### 2.7 Target-Compound Molecular Docking

The IGF-1R protein crystal structures were obtained from the RCSB Protein Data Bank (http://www.rcsb.org/pdb/) (Goodsell et al., 2020) and saved in pdb format. Diosgenin structures were downloaded through PubChem converted to. pdb format files via Open Babel (O'Boyle et al., 2011). Auto Dock Tools 1.5.6 software (Cosconati et al., 2010) was used to remove water molecules, to add nonpolar hydrogen bonds, to calibrate the Gasteiger charge and to save them as pdbqt format files. Diosgenin was performed with energy minimization, assigned the ligand atom type, calculated the charge, and saved in pdbqt format. Then, Auto Dock Tools 1.5.6 software was used to calculate the docking score to evaluate the matching degree and docking activity between a target and its ligand. A docking score < −4.25 can be considered as having binding activity; a score < −5.0 can be considered as having better binding activity; and a score < −7 can be considered as representing a strong docking activity between the ligand and the target. The binding model was visualized using PyMol2.3.0 software (Seeliger and de Groot, 2010).

### 2.8 Experimental Validation

#### 2.8.1 Animals and Treatments

Forty-eight male Sprague–Dawley (SD) rats (170–190 g, 6 weeks old) were purchased from Vital River Laboratory Animal Technology Co., Ltd (Beijing, China). After making adaption of 1 week, all rats were randomly distributed into four groups (*n* = 12 per group): Norm (normal control group), (methimazole group), MMI + L-Dio (methimazole plus diosgenin 20 mg kg^−1^ d^−1^ treatment group), and MMI + H-Dio (methimazole plus diosgenin 80 mg kg^−1^ d^−1^ treatment group). Briefly, 0.04% MMI was added to the drinking water for the three groups. One week later, rats were treated with 20 mg kg^−1^ d^−1^ or 80 mg kg^−1^ d^−1^ diosgenin by intraperitoneal injection. Correspondingly, the Norm and MMI groups were injected with the same amount of solvent (4% Tween-80) for 3 weeks (Han et al., 2009). All animals were anesthetized, and the thyroid glands were collected and fixed in 4% paraformaldehyde for hematoxylin-eosin (H&E) and immunohistochemistry staining. All animal operations were performed according to the animal guidelines and were approved by the Animal Ethics Committee of Shandong Provincial Hospital.

### 2.9 H&E Staining

The rats thyroid tissues were fixed in 4% paraformaldehyde and enclosed in paraffin. 4 μM slides were stained with hematoxylin and eosin (Shao et al., 2014). The histological changes were observed by upright microscopy (Axio Imager A2; Zeiss, Germany).

### 2.10 Immunohistochemical Analysis

Immunohistochemical staining was carried out for p-IGF-1R (AF3125; Affinity, China). Sections were incubated with the p-IGF-1R primary antibody at 4°C overnight, followed by incubation with HRP-conjugated secondary antibody. Finally, immunohistochemical reactions were visualized using DAB (Cai et al., 2014).

### 2.11 TUNEL Staining

A TUNEL apoptosis detection kit (KGA7071; KeyGEN, China) was used to discover cell nuclear DNA breakage during the late stage of apoptosis (He et al., 2019; Ma et al., 2020). Tissue sections were deparaffinized according to conventional methods. The sections were emulsified with proteinase K. After that, the paraffin sections were sequentially incubated in the dark with TdT buffer solution at 37°C for 1 h, streptavidin-fluorescein labeling solution was added for 30 min, and the sections were covered with DAPI containing an anti-fluorescence quenching mounting plate for 10 min. Finally, the apoptosis reaction of thyroid tissues were uncovered by a microimaging system (Axio Imager Z2, Zeiss, Germany).

### 2.12 Cells Culture and Processing

*Nthy-ori 3-1* cells (obtain from ATCC, Manassas, VA, United States) were cultured with RPMI (HyClone, United States) supplemented with 10% FBS (Gibco, United States) and 1% penicillin/streptomycin at 37°C and 5% CO2. When the thyrocytes were 70–80% confluent, they were starved in serum-free medium for 2 h and then preincubated with or without 100 ng/ml IGF-1 (cat. no. I1271, Sigma, United States; purity 95%) for 24 h. Then, cells were added with 10 μM diosgenin (cat. no. D1634, Sigma, United States; purity 93%) for another 24 h.

### 2.13 CCK-8 Assay

The cytotoxicity of IGF-1 and diosgenin was determined by CCK-8 assay. Briefly, *Nthy-ori 3-1* cells were seeded onto 96-well plates and cultured until they adhered completely. Then, the thyrocytes were induced by IGF-1, diosgenin or IGF-1 plus diosgenin at different concentrations for 24 or 48 h. Finally, 10 µl of CCK-8 solution was added and incubated at 37°C for 1 h. The absorbance at 450 nm was determined.

### 2.14 Western Blot Analysis

*Nthy-ori 3-1* cells were lysed in RIPA buffer containing protease and phosphatase inhibitors (Bimake, Houston, United States). The BCA protein quantitative analysis was used to determine the protein concentration. Proteins were resolved on 10% SDS–PAGE, and transferred to 0.22 μm PVDF membranes (Millipore, United States). Subsequently, the membranes were blocked with 5% skim milk for 1 h and were then probed with the following primary antibodies: p-IGF-1R (1:1,000; cat. no.3024; CST), P-IGF-1R (1:1,000; cat. no. AF3125; Affinity), p-ERK (1:1,000; cat. no. ab201015; Abcam), ERK (1:1,000; cat. no. ab184699; Abcam), p-AKT (1:1,000; cat. no. 66444-1-Ig; Proteintech), AKT (1:1,000; cat. no. 10176-2-AP; Proteintech), CASPASE3 (1:1,000; cat. no. 19677-1-AP; Proteintech), BCL2 (1:1,000; cat. no. 26593-1-AP; Proteintech), BAX (1:1,000; cat. no. 50599-2-AP; Proteintech), GAPDH (1:1,000; cat. no. 60004-1-Ig; Proteintech), and ß-actin (1:1,000; cat. no.66009-1-Ig; Proteintech). The membranes were gently incubated overnight at 4°C followed by incubation with HRP-conjugated secondary antibodies at room temperature for 1 h. The Alpha Q detection system was used for visualization.

### 2.15 Statistical Analysis

GraphPad Prism 8.0 software was used for Statistical analysis. Data are expressed as mean ± standard deviation (x ± s). For statistical analysis, means were compared using one-way ANOVA for multiple comparisons. A two-tailed *p* < 0.05 indicates that the difference is statistically significant.

## 3 Result

### 3.1 Compounds and Targets of DNR

**FIGURE 1:**
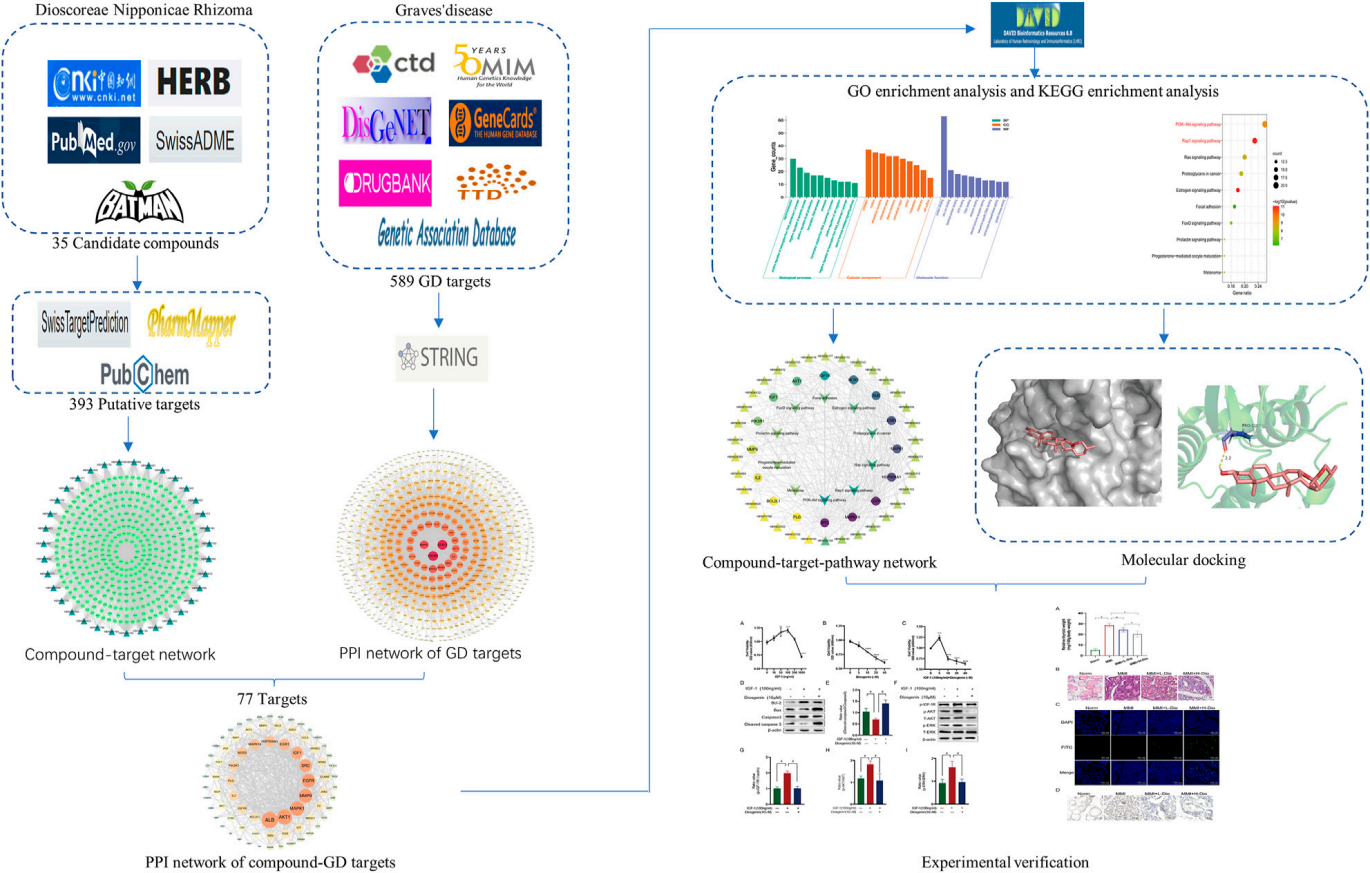
Workflow for investigating the mechanism of DNR in GD treatment.

**FIGURE 2:**
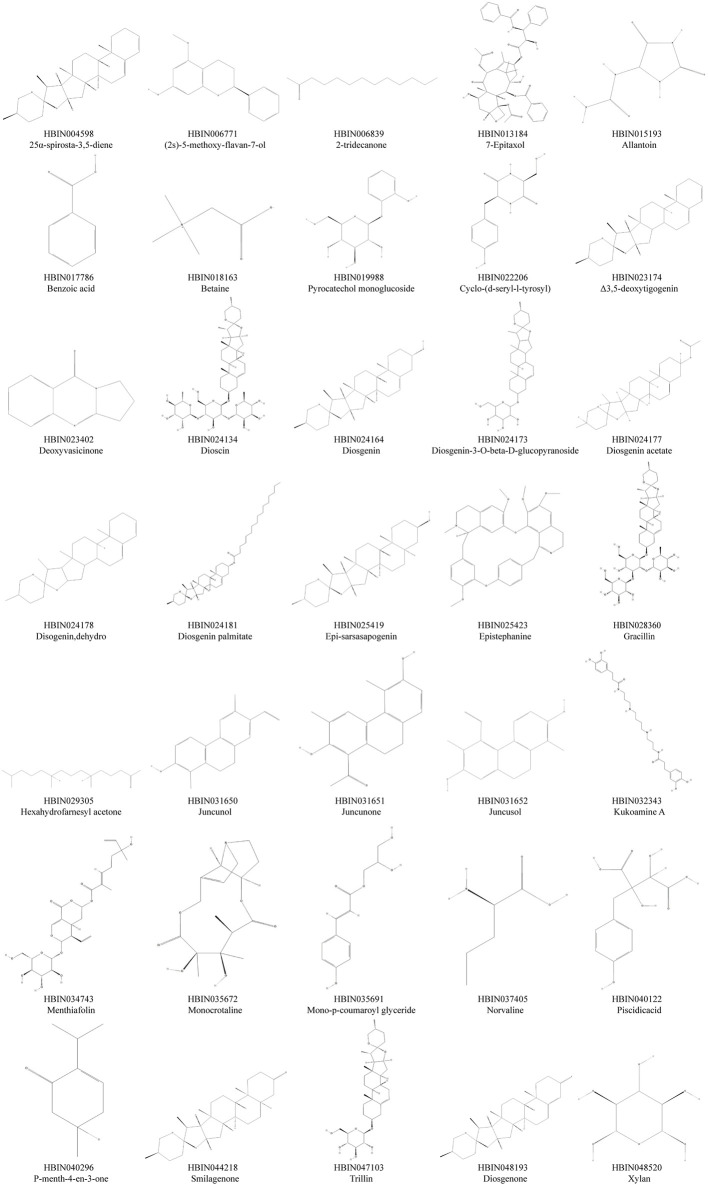
The chemical structure of active componds in DNR.

**FIGURE 3:**
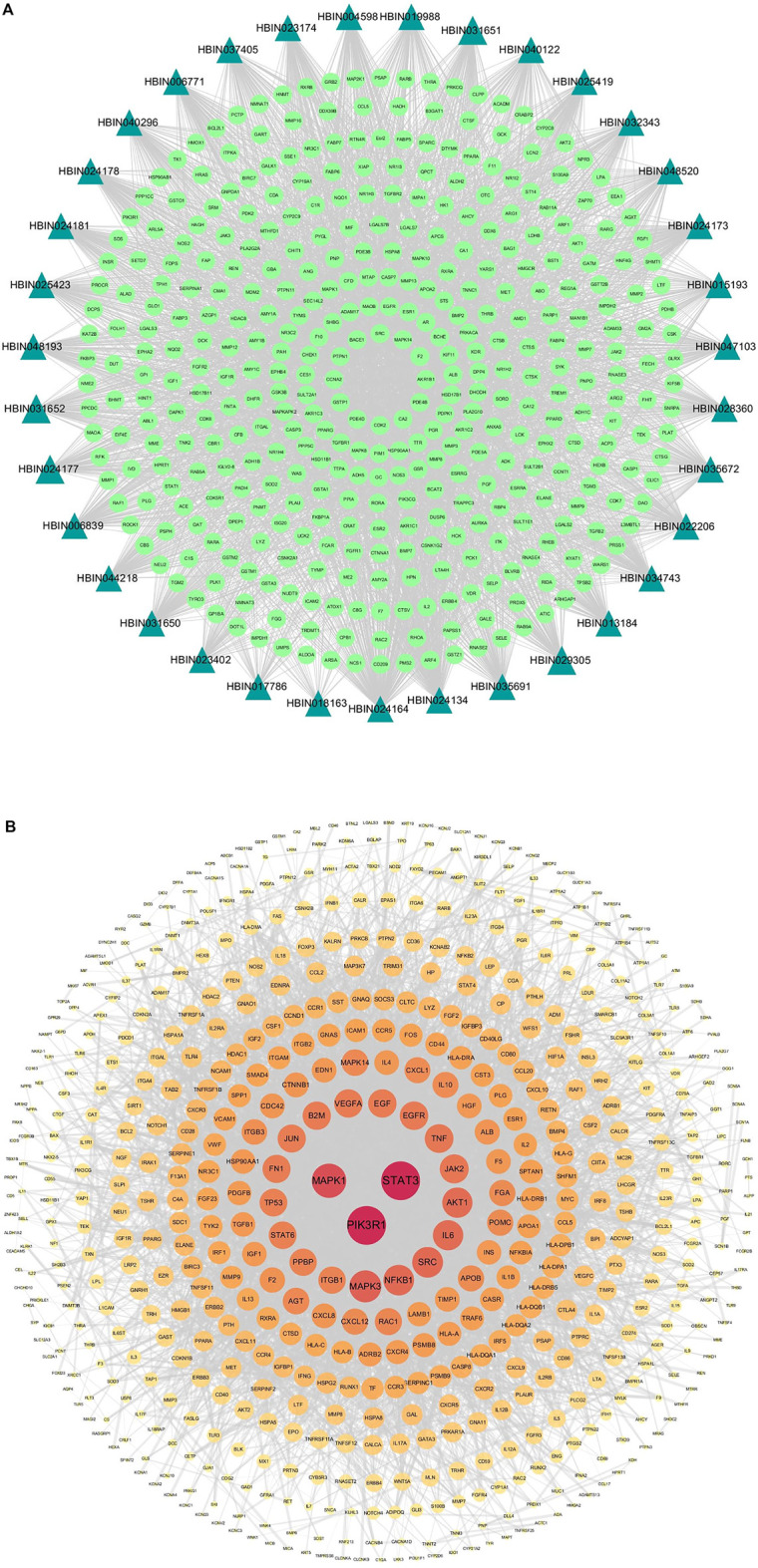
Compound-target network and protein-protein Interaction (PPI) network of GD targets. **(A)** The compound-target network of DNR. Active compounds were represented by bottle green triangle nodes, and targets were represented by pale green circular nodes. **(B)** The PPI network of GD targets. The nodes size and color depth image are proportional to their degree values.

A workflow of our study was summarized in Figure 1. We obtained 30 active compounds in DNR based on HERB database. Another five active compounds were incorporated into study after literature searching, including benzoic acid, pyrocatechol monoglucoside, cyclo-(d-seryl-l-tyrosyl), gracillin, and diosgenone. Ultimately, the structure of thirty-five active compounds of DNR were obtained and are presented in Figure 2, and their chemical information are showed in Table 1. We mainly counted the predicted targets of each component based on PharmMapper and obtained 394 potential targets of DNR. To further illustrate the correlationship between the 35 compounds and their predicted targets, we conducted a compound-target network (Figure 3A). A network topology analysis revealed that saponins account for approximately 40% of DNR, and the other ingredients are sterols, allantoin, resins, polysaccharides, starches, amino acids, flavonoids, and a small amount of 25α-Spirosta-3,5-diene. The composition is essentially the same as that reported in the literature (L. J. Zhang et al., 2012). Top six compounds were identified using the average degree (99.6) as threshold value: diosgenin (degree = 238), dioscin (degree = 198), mono-p-coumaroyl glyceride (degree = 102), hexahydrofarnesyl acetone (degree = 101), 7-epitaxol (degree = 101), and menthiafolin (degree = 100) (Supplementary Table S1). The targets enriched by diosgenin and dioscin were presented in significantly higher levels than other components, and the last four components are lipids and ketones, which are the basic components of DNR.

**TABLE 1:** Chemical information for the active compounds of DNR.

Number	HBIN ID	Compond	Composition	PubChem CID	MW	nON	nOHNH	MLogP
1	HBIN004598	25α-spirosta-3,5-diene	C_27_H_40_O_2_	337494	396.61	2	0	5.71
2	HBIN006771	(2s)-5-methoxy-flavan-7-ol	C_16_H_16_O_3_	14885875	256.3	3	1	2.45
3	HBIN006839	2-tridecanone	C_13_H_26_O	11622	198.34	1	0	3.54
4	HBIN013184	7-Epitaxol	C_47_H_51_NO_14_	184492	853.91	14	4	1.7
5	HBIN015193	Allantoin	C_4_H_6_N_4_O_3_	204	158.12	3	4	−1.85
6	HBIN017786	Benzoic acid	C_7_H_6_O_2_	243	122.12	2	1	1.6
7	HBIN018163	Betaine	C_5_H_11_NO_2_	247	117.15	2	0	−3.67
8	HBIN019988	Pyrocatechol monoglucoside	C_12_H_16_O_7_	9900144	272.25	7	5	−1.49
9	HBIN022206	Cyclo-(d-seryl-l-tyrosyl)	C_12_H_14_N_2_O_4_	3082196	250.25	4	4	−0.73
10	HBIN023174	Δ3,5-deoxytigogenin	C_27_H_40_O_2_	131751534	396.61	2	0	5.71
11	HBIN023402	Deoxyvasicinone	C_11_H_10_N_2_O	68261	186.21	2	0	2.04
12	HBIN024134	Dioscin	C_45_H_72_O_16_	119245	869.04	16	8	2.61
13	HBIN024164	Diosgenin	C_27_H_42_O_3_	99474	414.62	3	1	4.94
14	HBIN024173	Diosgenin-3-O-beta-D-glucopyranoside	C_33_H_52_O_8_	129716073	576.76	8	4	2.41
15	HBIN024177	Diosgenin acetate	C_29_H_44_O_4_	225768	456.66	4	0	5.18
16	HBIN024178	Disogenin,dehydro	C_27_H_40_O_2_	587211	396.61	2	0	5.71
17	HBIN024181	Diosgenin palmitate	C_43_H_72_O_4_	21159048	653. 02	4	0	7
18	HBIN025419	Epi-sarsasapogenin	C_27_H_44_O_3_	12304430	416.64	3	1	5.08
19	HBIN025423	Epistephanine	C_37_H_38_N_2_O_6_	5317122	606.71	8	0	3.48
20	HBIN028360	Gracillin	C_45_H_72_O_17_	159861	885.04	17	9	−1.46
21	HBIN029305	Hexahydrofarnesyl acetone	C_18_H_36_O	10408	268.48	1	0	4.79
22	HBIN031650	Juncunol	C_18_H_18_O	85926875	250.33	1	1	4.12
23	HBIN031651	Juncunone	C_18_H_18_O_3_	327720	282.33	3	2	2.58
24	HBIN031652	Juncusol	C_18_H_18_O_2_	72740	266.33	2	2	3.46
25	HBIN032343	Kukoamine A	C_28_H_42_N_4_O_6_	5318865	530.66	8	8	0.76
26	HBIN034743	Mentdiafolin	C_26_H_36_O_12_	76960104	540.56	12	5	−0.44
27	HBIN035672	Monocrotaline	C_16_H_23_NO_6_	9415	325.36	7	2	0.24
28	HBIN035691	Mono-p-coumaroyl glyceride	C_12_H_14_O_5_	5319874	238.24	5	3	0.48
29	HBIN037405	Norvaline	C_5_H_11_NO_2_	439575	117.15	3	2	−2.2
30	HBIN040122	Piscidicacid	C_11_H_12_O_7_	120693	256.21	7	5	−0.6
31	HBIN040296	P-menth-4-en-3-one	C_10_H_16_O	107372	152.23	1	0	2.2
32	HBIN044218	Smilagenone	C_27_H_42_O_3_	160498	414.62	3	0	4.94
33	HBIN047103	Trillin	C_33_H_52_O_8_	11827970	576.76	8	4	2.41
34	HBIN048193	Diosgenone	C_27_H_40_O_3_	10251134	412.6	3	0	4.83
35	HBIN048520	Xylan	C_5_H_10_O_6_	50909243	166.13	6	5	−2.73

### 3.2 The Target Network of GD

In our study, 589 targets were taken as potential disease genes of GD (Supplementary Table S2) and their interaction relationship was shown in PPI network (Figure 3B). Eight targets were identified highly related to the pathological process of GD, including PIK3RI (degree = 105), STAT3 (degree = 105), MAPK1 (degree = 84), MAPK3 (degree = 73), NF-κB (degree = 67), SRC (degree = 67), IL6 (degree = 65), and AKT1 (degree = 65) (Supplementary Table S3). These eight targets played important roles in GD.

### 3.3 PPI Network of DNR’s Compound-GD Targets

**FIGURE 4:**
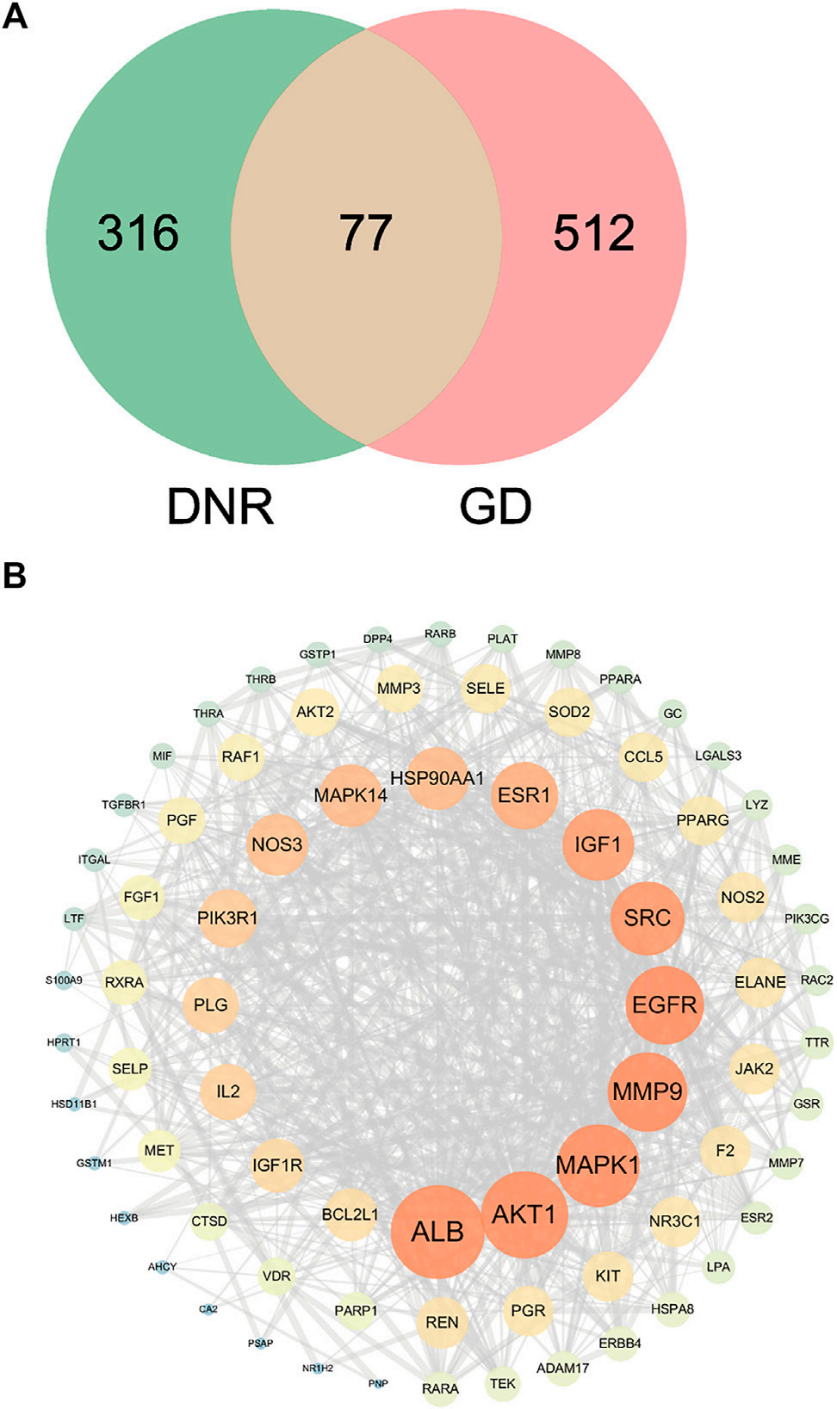
Venn diagram and PPI network for overlapped targets. **(A)** Venn diagram of overlapping genes of DNR and GD. **(B)** PPI network of GD’s active compounds and their related targets. The node size and color depth are proportional to their degree values.

In order to discover overlapped targets, we mapped 393 predicted genes of DNR with 590 related genes of GD. 77 overlapped genes were taken as possible therapeutic targets against GD and expressed by a Venn diagram (Figure 4A). Based on PPI network topological analyzing, 77 targets were exhibited in descending order by their degree, as shown in Supplementary Table S4, and the average degree of 77 putative genes was 17.7. As shown in Figure 4B, the genes were exhibited in a concentric circle according to their degree, and the innercircle was comprised of 16 core targets: ALB (degree = 60), AKT1 (degree = 54), MAPK1 (degree = 50), MMP9 (degree = 47), EGFR (degree = 46), SRC (degree = 42), IGF-1 (degree = 40), ESR1 (degree = 36), MAPK14 (degree = 32), HSP90AA1 (degree = 32), NOS3 (degree = 31), PIK3R1 (degree = 28), IL2 (degree = 26), PLG (degree = 26), IGF-1R (degree = 24), and BCL2L1 (degree = 23). These targets are the hub targets of the network and the core targets of DNR to treat GD and will be used in subsequent molecular docking studies.

### 3.4 Enrichment Analyses of GO and KEGG

**FIGURE 5:**
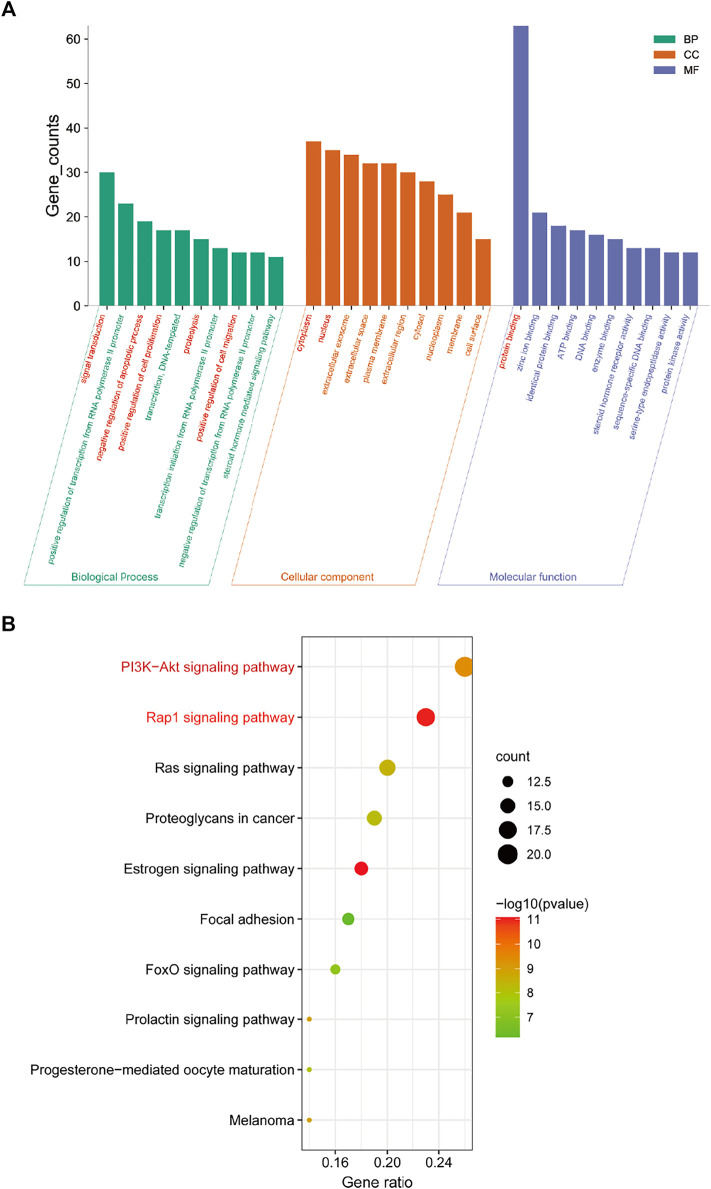
Enrichment analysis for key targets. **(A)** The GO enrichment analysis of key targets. BP means biological processes, CC means cell component, MF means molecular function. **(B)** The KEGG pathway analysis of key targets. Nodes size and color depth are proportional to the gene ratio involved in the pathways.

In order to further illustrate the potential pharmacological mechanism of DNR systematically, 77 common genes were enriched by DAVID. GO enrichment analysis screened out 84 biological processes (BP), 43 molecular functions (MF) and 36 cell components (CC) according to false discovery rate (FDR) < 0.05 and *p* value < 0.05. The top 10 GO items were selected based on counts of hit genes (Figure 5A). The interactions between the common targets of GD and DNR were mainly in the cytoplasm (GO:0005737) and nucleus (GO:0005634) and mainly through the interconnection between proteins (GO:0005515). For biological processes, the targets were mainly enriched in signal transduction (GO:0007165), apoptosis (GO:0043066), cell proliferation (GO:0008284), proteolysis (GO:0006508) and cell migration (GO:0030335). The top ten signal pathways are displayed in Figure 5B according to the number of targets enriched by KEGG and the *p* values. Two signaling pathways with the most enriched targets were obtained by analysis, namely, the PI3K signaling pathway (hsa04151) and the Rap1 signaling pathway (hsa04015) (Table 2). The results above illustrated the crucial mechanism of action underlying GD therapy.

**TABLE 2:** Information for top 10 pathways.

Pathway ID	Pathway name	Count	*p* Value
hsa04151	PI3K-Akt signaling pathway	20	4.36E-10
hsa04015	Rap1 signaling pathway	18	1.04E-11
hsa04014	Ras signaling pathway	16	3.33E-09
hsa05205	Proteoglycans in cancer	15	6.06E-09
hsa04915	Estrogen signaling pathway	14	8.30E-12
hsa04510	Focal adhesion	13	6.29E-07
hsa04068	FoxO signaling pathway	12	6.05E-08
hsa05218	Melanoma	11	1.29E-09
hsa04917	Prolactin signaling pathway	11	1.29E-09
hsa04914	Progesterone-mediated oocyte maturation	11	9.88E-09

### 3.5 Compound-Core Target-Pathway Network

**FIGURE 6:**
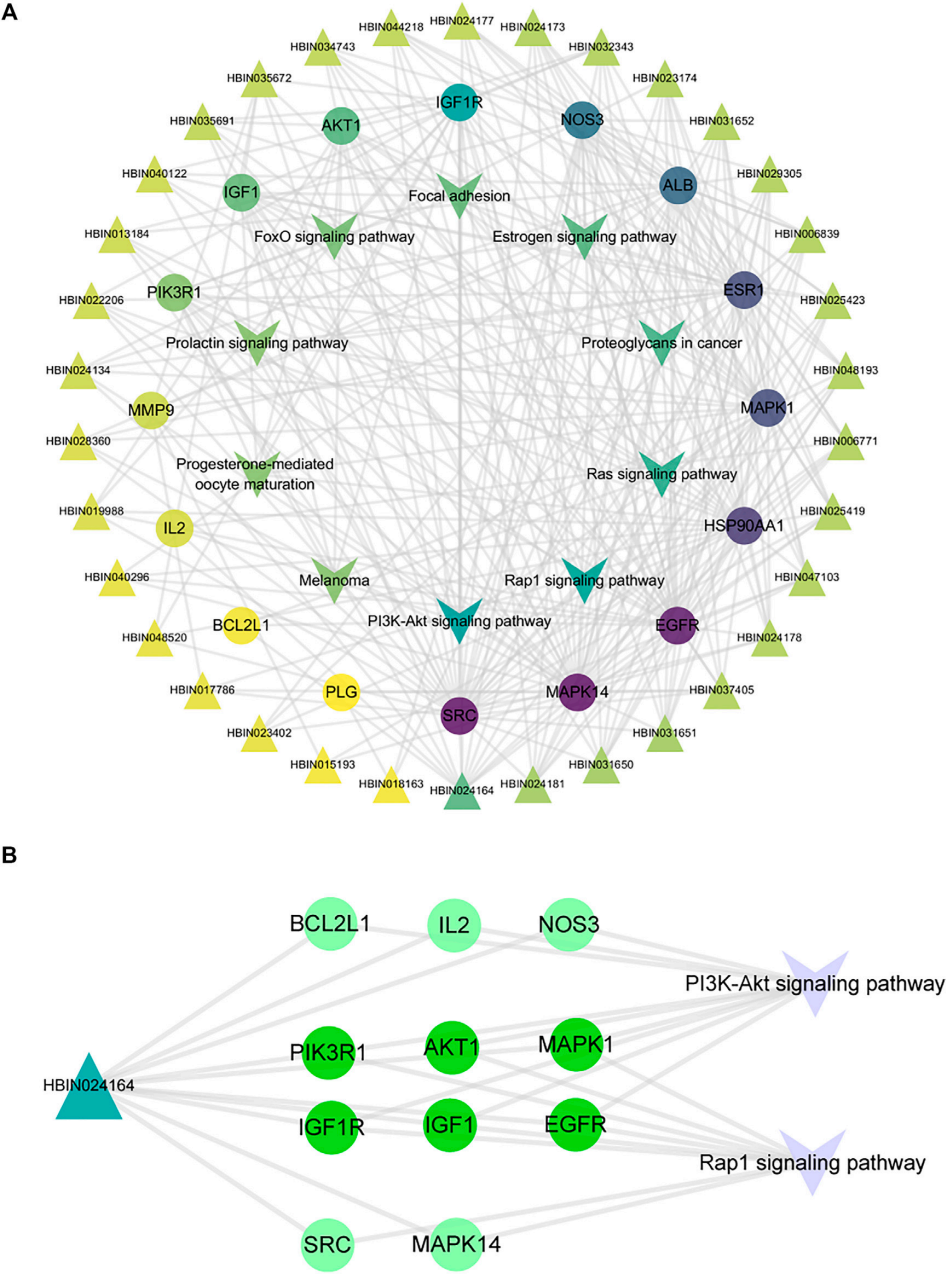
Compound-target-pathway network of DNR against GD. **(A)** Compound-core-target-pathway network. Triangle nodes represent active compounds, whose colors are proportional to their degree. Circular nodes represent core targets. The nodes color are showed from purple to yellow in descending order of degree values. Inverted triangle nodes represent signaling pathways. **(B)** The targets of diosgenin enrichment in the PI3K and Rap1 signaling pathways. The dark green targets enriched by diosgenin and two pathways together.

Through the connection of potential signaling pathways, compounds, as well as core targets, a compound-core target-pathway network was established. As exhibited in Figure 6A, DNR mainly acted through the PI3K-Akt and Rap1 signaling pathways. There were 23 components with degree values greater than the average (6.9). We selected the first four main components with degree ≥9: diosgenin (degree = 14), diosgenin palmitate (degree = 9), juncunol (degree = 9), and juncunone (degree = 9). Diosgenin is a crucial compond in the treatment of GD by DNR. Juncunol and juncunone are phenanthrene derivatives that play roles of clearing the heart, reducing fire, and promoting water and leaching. Subsequently, to observe the relationship between diosgenin and the first two pathways in more detail, we extracted a subnet to show the mapping path of diosgenin at key targets (Figure 6B). These results confirmed that there were six common targets of diosgenin, among which PI3K and Rap1 signaling pathways showing effects.

### 3.6 Molecular Docking Analysis

**FIGURE 7:**
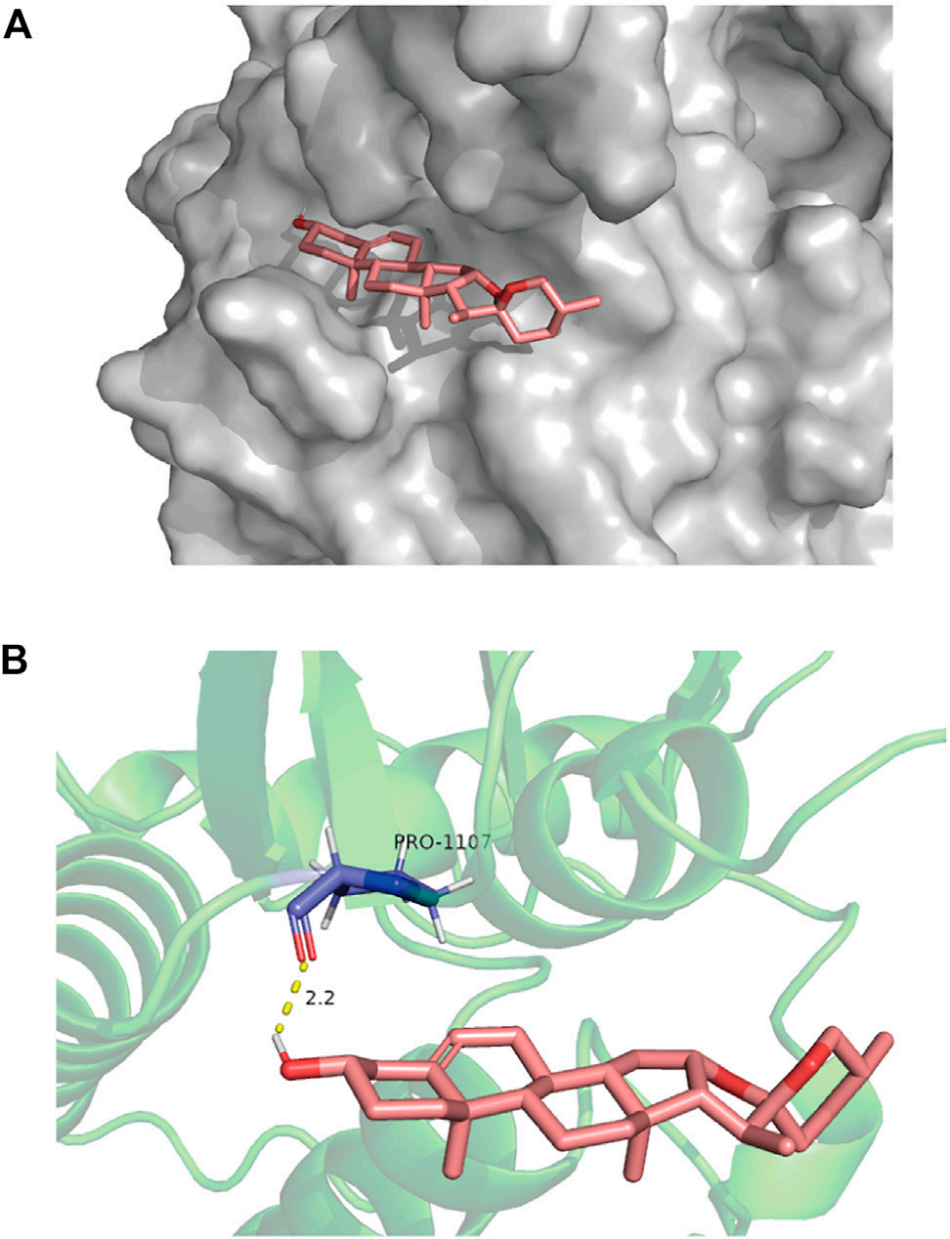
The molecular docking of IGF-1R and diosgenin is shown. **(A)** The picture shows that the structure of diosgenin in 3D is located in the binding pocket of IGF-1R. The pink image on the right represents diosgenin. **(B)** The protein target that binds to diosgenin is in the shape of a blue rod, and the binding sites are connected by yellow hydrogen bonds. The length of the hydrogen bonds is indicated next to the bond.

To test whether diosgenin acts on IGF-1R, we predicted that the core compound diosgenin and the hub target IGF-1R were molecularly docked. The greater the docking affinity, the better the binding ability of the compound to its target. In the molecular docking results, IGF-1R and diosgenin are connected to each other through hydrogen bonds, and they have high binding affinity (-8.66 kcal/mol) (Figure 7).

### 3.7 Diosgenin Induces Apoptosis in Nthy-ori 3-1 Cells by Inhibiting the Phosphorylation of IGF-1R *in vitro*

**FIGURE 8:**
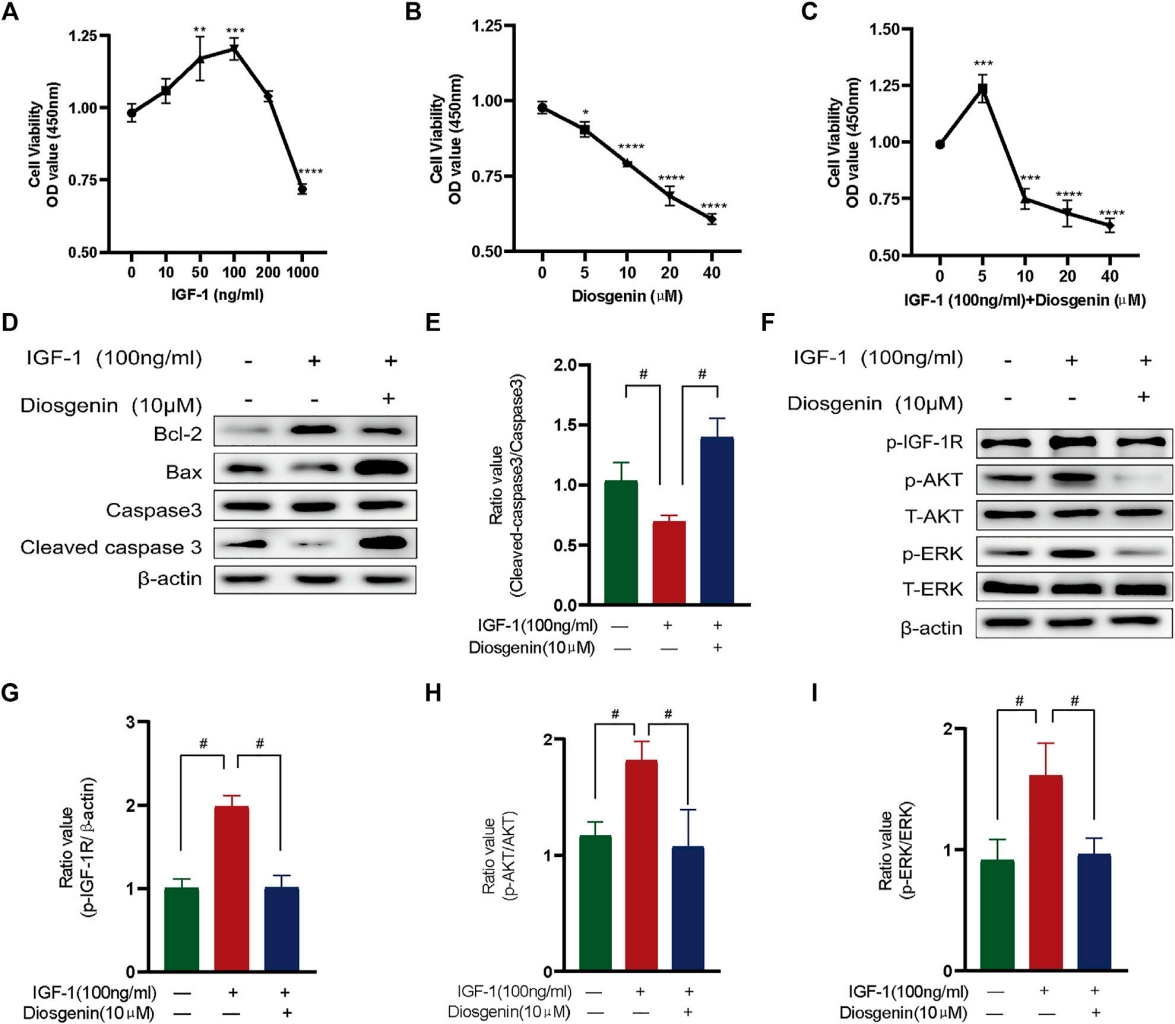
Diosgenin induces apoptosis in *Nthy*-*ori* *3*-*1* cells by inhibiting the phosphorylation of IGF-1R *in vitro* phosphorylation of IGF-1R *in vitro*. **(A)** Cell viability with IGF-1 (ng/ml) at increasing concentrations after culturing for 48 h. **(B)** Cell viability after 24 h of culture with diosgenin (μM) at different concentrations. **(C)** Cell viability with IGF1 (100 ng/ml) and diosgenin (μM) at different concentrations. **(D)** Apoptosis-related proteins were detected using WB as described above. *ß*-actin was used as an internal reference. **(F)** Diosgenin devitalized the PI3K/AKT and Raf1/MEK signaling pathways by inhibiting p-IGF-1R. **(E, G–I)** Data are shown as the mean ± standard deviation of three independent experiments. *p < 0.05, **p < 0.01, ***p < 0.001 and ****p < 0.0001 compared with the 0 group. #*p* < 0.05 compared with the IGF-1 group.

To verify the mechanism of diosgenin’s treatment of GD, we first investigated the effective concentration of diosgenin. The CCK8 results showed that IGF-1 significantly promoted cell proliferation in the concentration range of 0–100 ng/ml but showed obvious toxicity to *Nthy*-*ori* *3*-*1* *cells* at a concentration greater than 100 ng/ml (Figure 8A); therefore, 100 ng/ml IGF-1 was used for follow-up experiments. Diosgenin significantly inhibited the viability of thyrocytes in a dose-dependent manner (Figure 8B). Moreover, at concentrations of 5–40 μM, diosgenin had an increasingly stronger inhibitory effect on *Nthy-ori 3-1* cells pretreated with 100 ng/ml IGF-1. When we added 20 μM or even 40 μM diosgenin to IGF-1-induced cells, we found that *Nthy*-*ori* *3*-*1* cell vability were obviously decreased. Thus, we chose 10 μM diosgenin as our treatment concentration in the ensuing experiments (Figure 8C). To verify whether diosgenin contributes to the apoptosis of *Nthy*-*ori* *3*-*1* cells induced by IGF-1, we detected the protein levels of BCL2, BAX and caspase-3 by Western blot (Figures 8D,E). The thyrocytes were exposed to diosgenin (10 μM) with or without 100 ng/ml IGF-1 for 48 h. The levels of BCL2 were increased, and BAX and cleaved-caspase3 were decreased after pretreatment with IGF-1. These results indicated that IGF-1 inhibited the activation of apoptosis. In contrast, diosgenin successfully promoted apoptosis in IGF-1-induced cells. To verify whether diosgenin contributes to thyrocytes apoptosis through the inhibition of IGF-1R phosphorylation, we observed the activation of IGF-1R and its downstream pathways (Figures 8F–I). We found that IGF-1R phosphorylation was greatly increased in IGF-1-pretreated thyrocytes. After treated with diosgenin (10 μM), the phosphorylation level of IGF-1R decreased significantly, as did the two downstream pathways AKT and ERK. While the expression of the antiapoptotic protein BCL2 decreased, the proapoptotic protein BAX increased. Ultimately, caspase-3 was completely cleaved to its terminal fragments (17 kD). These results implied that diosgenin initiated apoptosis by inhibiting the phosphorylation of IGF-1R.

### 3.8 Diosgenin Could Suppress Thyrocyte Proliferation in MMI-Induced Rats

**FIGURE 9:**
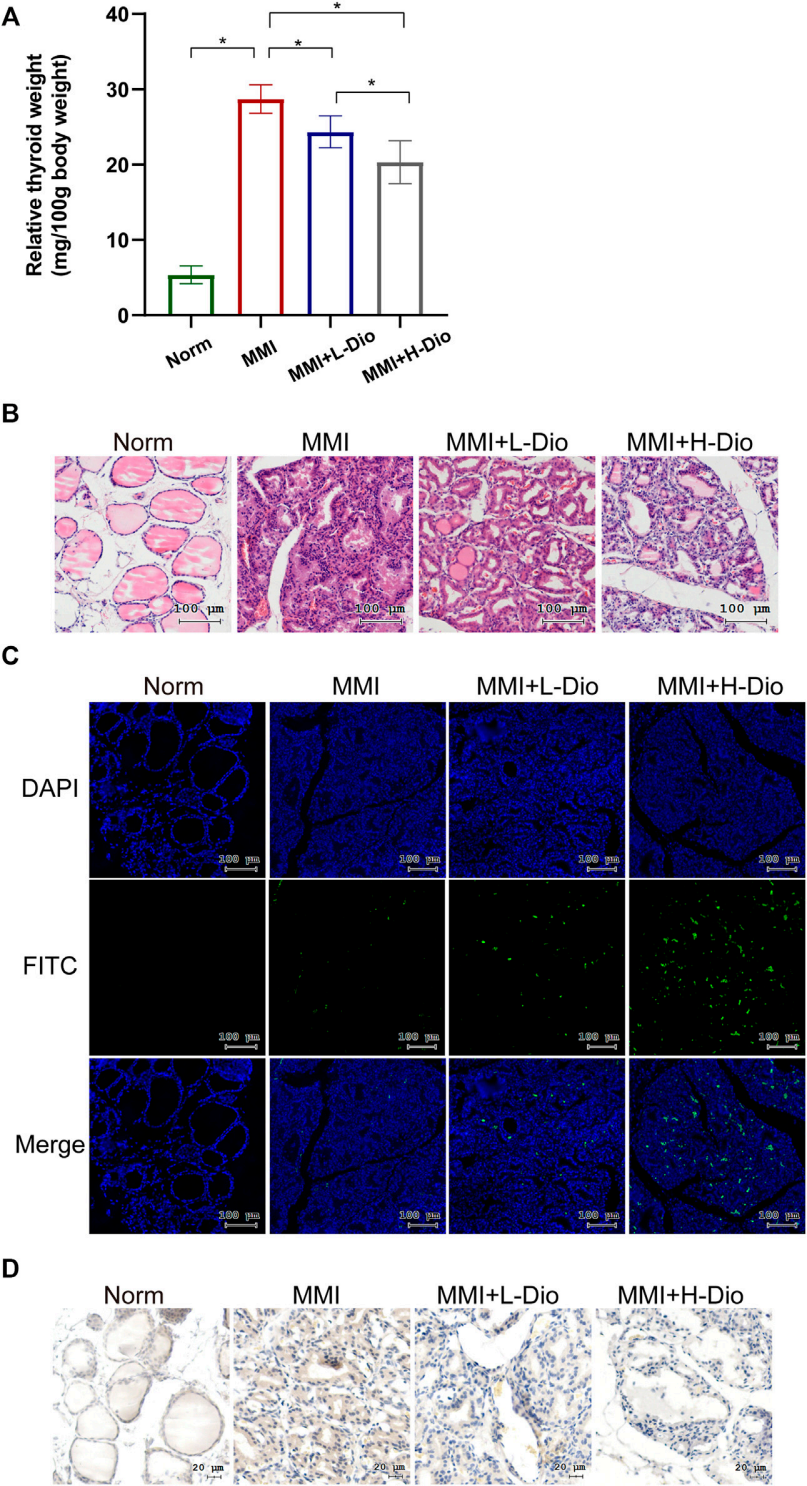
Diosgenin could inhibit thyrocyte proliferation in MMI-induced rats. **(A)** The effects of diosgenin treatment on goiter. **(B)** After 3 weeks of diosgenin treatment, **(A–D)** refer to histological changes in thyroid glands stained with H&E (magnification, ×200). **(C)** The TUNEL kit allows apoptotic cells to emit green light through the FITC channel. The nucleus is stained by DAPI. **(D)** Immunohistochemical staining of p-IGF-1R in the thyroid after 3 weeks of diosgenin treatment.

To confirm the inhibition of GD by diosgenin *in vivo*, we constructed a rat model of GD goiter. Based on previous research (Han et al., 2009; Cai et al., 2014) and following the best practice in pharmacological research (Heinrich et al., 2020), the safe, effective and non-toxic concentration of MMI and diosgenin were selected. The relative thyroid weight of the MMI group was significantly higher than that of the Norm group. Compared with the MMI group, diosgenin reduced the thyroid weight in the MMI + L-Dio and MMI + H-Dio groups by 17% (*p* < 0.05) and 30% (*p* < 0.05), respectively. Overall, as the dose of diosgenin increased, the relative weight of the thyroid gland gradually decreased compared with the MMI group (Figure 9A). H&E staining revealed that the monolayer of thyroid follicular epithelial cells in the Norm group was neatly arranged in a cubic shape, the structure of the follicles was clear, and the cytoplasm was abundant. However, in the MMI group, diffuse hyperplasia of thyroid follicular epithelial cells showed high columnar hyperplasia, and some showed papillary hyperplasia. The shape of the follicles was irregular, smaller, or even locked. The glia in the follicle cavity were significantly reduced, and absorption vacuoles of epithelial cells of different sizes were present around the follicle. Microscopy also revealed that the interstitial blood vessels were abundant and congested. These morphological abnormalities were relieved through diosgenin treatment. Surprisingly, most of the thyroid follicle morphology was restored in the MMI + H-Dio group (Figure 9B). To verify the effects of diosgenin on thyrocyte apoptosis, a TUNEL fluorescence staining assay was performed (Figure 9C). Much stronger green fluorescence was observed in the MMI + H-Dio groups, which revealed higher cell necrosis and apoptosis after treatment with high-dose diosgenin. To investigate the potential mechanism underlying the proapoptotic effects of diosgenin, immunohistochemical staining was used to examined the protein expression of IGF-1R in thyroid tissue. As shown in Figure 9D, the phosphorylation level of IGF-1R in the MMI group was upregulated obviously compared with that in the Norm group. However, this increase obviously reduced after diosgenin treatment.

## Discussion

Dioscin is a water-soluble steroidal saponin component extracted from DNR that can be hydrolyzed into diosgenin (Yan et al., 2013). Diosgenin could be considered as an important basic natural material for producing steroid hormone drugs, and it has a hormone-like effect, but the side effects are far lower than those of hormones (Parama et al., 2020). It could be absorbed quickly and its oral bioavailability reach up to 80.88% (Ru et al., 2014). In order to get diosgenin with better quality and purity from botanical sources, various techniques including elicitation, genetic transformations and bioconversions were employed (Nazir et al., 2021) (Chao et al., 2017; Qiao et al., 2018). The content of diosgenin in Medicinal Plants could be determinated accurately by enzyme-linked immunosorbent assay or a micellar electrokinetic capillary chromatographic (MECC) (J. Li et al., 2010; N. Wang et al., 2019), and the water contents in diosgenin could be assayed with high accuracy using the mathematical model of near-infrared spectroscopy technology (M. Zhang et al., 2019). For the past few years, the biological activities of diosgenin has drawn much attention by studied *in vivo* and *in vitro* (Leger et al., 2004; J.; Liagre et al., 2004; M. J.; Liu et al., 2005; Liagre et al., 2004; Srinivasan et al., 2009; Li et al., 2010; Ru et al., 2014; Chao et al., 2017; Qiao et al., 2018; Kessler et al., 2019; N.; Wang et al., 2019; M. Zhang et al., 2019; Mohamadi-Zarch et al., 2020). In previous studies, we found that diosgenin (Mu et al., 2012) promoted apoptosis of human primary thyroid cells under the action of IGF-1 through the caspase signaling pathway, but its specific mechanism is not clear. At the same time, it does not affect the serum TT4 level or the size of the thyroid gland in normal mice (Bian et al., 2011; Cai et al., 2014). Therefore, diosgenin may be a safe antithyroid drug to avoid hypothyroidism.

**FIGURE 10:**
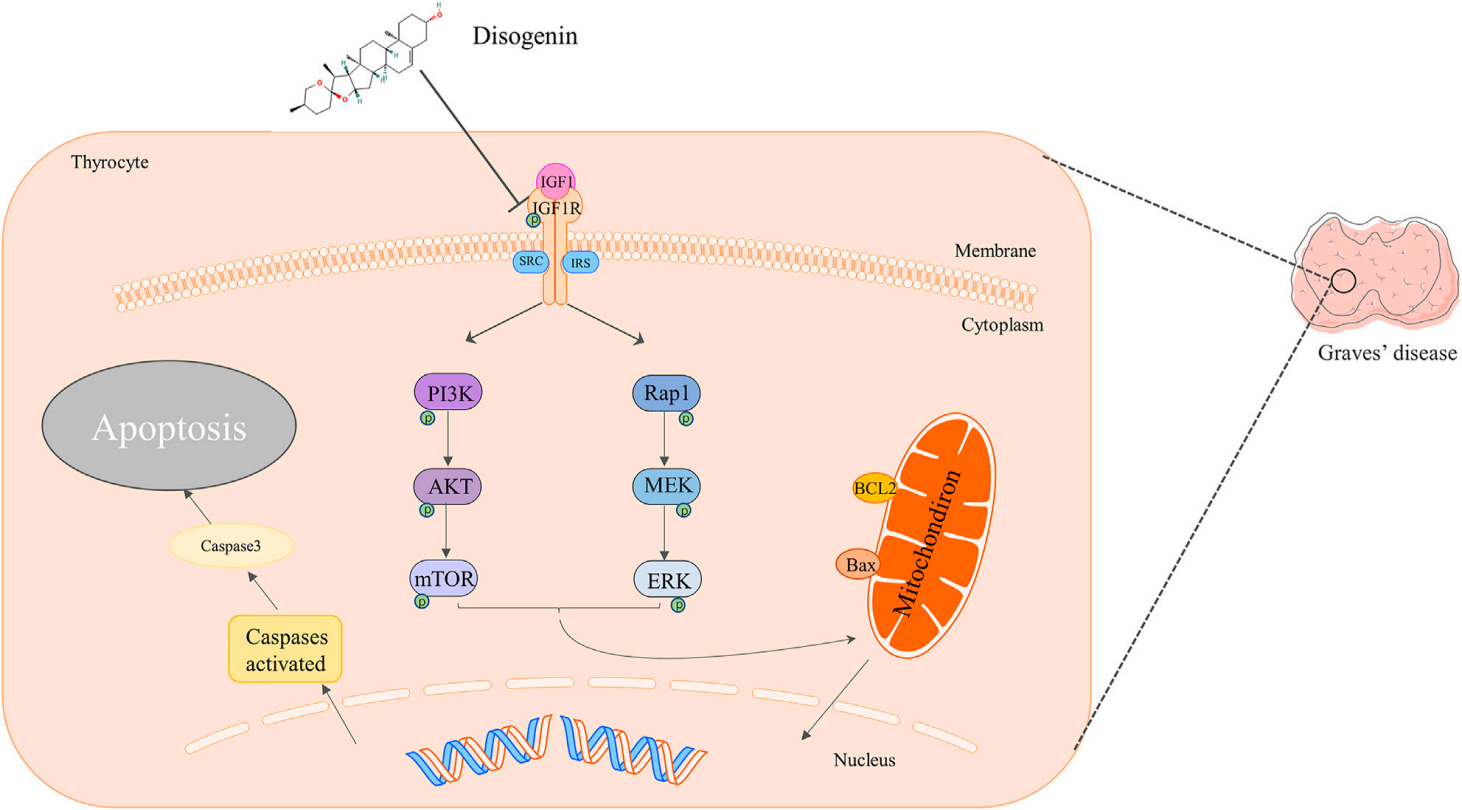
Mechanism of diosgenin in the treatment of Graves’ disease.

In this study, 589 targets of GD were collected from multiple databases, suggesting that the occurrence of GD may be caused by multiple targets. Through the HERB database and literature searches, 35 active components and 393 targets of DNR were screened, among which diosgenin was predicted as the most important compond. Then, the compond collected from GD and DNR were intersected to obtain 77 common targets, which can be speculated to be candidate therapeutic targets of DNR against GD. Based on topological property of PPI, 16 core targets for the treatment of GD by DNR were selected. Through GO and KEGG analyses of 77 common targets, it was found that the interaction between GD and DNR occurred mainly through the interconnection between proteins, and the main mechanisms were signal transduction, apoptosis and proliferation. KEGG analysis showed that DNR may play a role through the PI3K and Rap1 signaling pathways. Next, we established compond-core target-pathway co-module and found that diosgenin was enriches fourteen core targets in sixteen. Then, we extracted diosgenin, two key signaling pathways and corresponding targets from the PPI network, and we observed that most targets were in IGF-1, IGF-1R and its downstream pathways. IGF-1 is a growth factor that can promote cell proliferation, differentiation and angiogenesis. IGF-1R is a transmembrane tyrosine kinase protein with a tetramer structure composed of two subunits, *a* and *ß*. The combination of IGF-1 and IGF-1R can activate the PI3K/Akt and Raf1/MEK signaling pathways to suppress the apoptosis and promote the proliferation of tumor cells (Ryan and Goss, 2008; Weroha and Haluska, 2008; Bruchim et al., 2009). IGF-1 and IGF-1R also play important roles in the formation and development of GD (Minuto et al., 1989), thyroid carcinoma and thyroid nodules (Karagiannis et al., 2020; Y. J.; Liu et al., 2011; Y. J.; Liu et al., 2013). Transgenic mice overexpressing IGF-1 and IGF-1R could feedback the decrease of thyroid-stimulating hormone, suggesting that IGF-1 and IGF-1R could stimulate thyroid function to some extent (Clément et al., 2001). The expression of IGF-1R was significantly increased in thyroid cells and orbital fibroblasts in GD patients (Smith, 2003). Teprotumumab, a monoclonal antibody IGF-1 receptor antagonist, has achieved important curative effects in Graves’ ophthalmopathy (Lanzolla et al., 2020). Therefore, IGF-1R plays vital part in the onset and development of GD (Tsui et al., 2008). This showed that drugs targeting the IGF-1 receptor can be very promising in the treatment of GD. Hence, we conducted validation experiments according to the standards of research in the field of pharmacology (Heinrich et al., 2018). The molecular docking showed that the binding activity of IGF-1R to diosgenin was high and that the structure was relatively stable. Then we used IGF-1 to induce excessive thyrocytes proliferation *in vitro* (Brandi et al., 1987; Bian et al., 2011; Mu et al., 2012; Yamanaka et al., 2012). Some predicted hub targets of diosgenin from network pharmacology were verified by Western blot. The results showed that diosgenin may inhibit the phosphorylation activation of IGF-1R compared with the IGF-1 group and then inhibit the PI3K and Raf1 pathways. This inhibition can lead to a decrease in the antiapoptotic protein BCL2 and an increase in the proapoptotic protein Bax, and finally activated caspase 3 to cleaved caspase 3, resulting in irreversible apoptosis. *In vivo,* we used MMI to compensate for thyroid enlargement in rats. The results showed that diosgenin reduced goiter in GD rats in a dose-dependent manner by promoting thyroid cell apoptosis. In addition, the phosphorylation of IGF-1R decreased with increasing thyroid cell apoptosis. This research confirmed that diosgenin could target and inhibit IGF-1R by molecular docking and *in vivo* and *in vitro* experiments. These results supported our prediction from network pharmacology. However, our study also had potential limitations. First, although network pharmacology could help us to realize the interactions between disease-specific molecules and drug-targeting proteins in the network target, it cannot tell whether the effect of the drug on the target is up-regulated or down-regulated (S. Li, 2021). Secondly, about the common targets of DNR and GD, we didn’t analyze whether these targets have statistically significant. From another point of view, DNM has many componds related to saponins, it is also a good thought to explore their similarities and differences with network pharmacology. In the future, more extensive experiments are needed to reveal the above possibilities, such as proving the direct combined of diosgenin with IGF-1R. Based on the above results, we have drawn a simple diagram of the mechanism (Figure 10).

## Conclusion

In conclusion, this work identified the mechanism of diosgenin on Graves’ disease at the system level through network pharmacological analysis and biological verification. We confirmed that diosgenin can bind to IGF-1R and inhibit the activation of Akt and ERK pathways by inhibiting the phosphorylation of IGF-1R, resulting in increasing apoptosis of hyperproliferated thyroid cells in Graves’ disease and alleviating goiter. Therefore, diosgenin plays an important role in the treatment of Graves’ disease.

## Data Availability

The datasets presented in this study can be found in online repositories. The names of the repository/repositories and accession number(s) can be found in the article/Supplementary Material.
