# Supplementary material for: Diosgenin From Dioscorea Nipponica Rhizoma Against Graves’ Disease—On Network Pharmacology and Experimental Evaluation
Source: Front Pharmacol. 2022 Jan 24;12:806829. doi: 10.3389/fphar.2021.806829 (PMC8819592; doi:10.3389/fphar.2021.806829)
Supplement: Supplementary file 1 [file Table1.docx]

**Table 1** Chemical information for the active compounds of DNR.

| Number | HBIN ID | Compond | Composition | PubChem CID | MW | nON | nOHNH | MLogP |
| --- | --- | --- | --- | --- | --- | --- | --- | --- |
| 1 | HBIN004598 | 25α-spirosta-3,5-diene | C27H40O2 | 337494 | 396.61 | 2 | 0 | 5.71 |
| 2 | HBIN006771 | (2s)-5-methoxy-flavan-7-ol | C16H16O3 | 14885875 | 256.3 | 3 | 1 | 2.45 |
| 3 | HBIN006839 | 2-tridecanone | C13H26O | 11622 | 198.34 | 1 | 0 | 3.54 |
| 4 | HBIN013184 | 7-Epitaxol | C47H51NO14 | 184492 | 853.91 | 14 | 4 | 1.7 |
| 5 | HBIN015193 | Allantoin | C4H6N4O3 | 204 | 158.12 | 3 | 4 | -1.85 |
| 6 | HBIN017786 | Benzoic acid | C7H6O2 | 243 | 122.12 | 2 | 1 | 1.6 |
| 7 | HBIN018163 | Betaine | C5H11NO2 | 247 | 117.15 | 2 | 0 | -3.67 |
| 8 | HBIN019988 | Pyrocatechol monoglucoside | C12H16O7 | 9900144 | 272.25 | 7 | 5 | -1.49 |
| 9 | HBIN022206 | Cyclo-(d-seryl-l-tyrosyl) | C12H14N2O4 | 3082196 | 250.25 | 4 | 4 | -0.73 |
| 10 | HBIN023174 | Δ3,5-deoxytigogenin | C27H40O2 | 131751534 | 396.61 | 2 | 0 | 5.71 |
| 11 | HBIN023402 | Deoxyvasicinone | C11H10N2O | 68261 | 186.21 | 2 | 0 | 2.04 |
| 12 | HBIN024134 | Dioscin | C45H72O16 | 119245 | 869.04 | 16 | 8 | 2.61 |
| 13 | HBIN024164 | Diosgenin | C27H42O3 | 99474 | 414.62 | 3 | 1 | 4.94 |
| 14 | HBIN024173 | Diosgenin-3-O-beta-D-glucopyranoside | C33H52O8 | 129716073 | 576.76 | 8 | 4 | 2.41 |
| 15 | HBIN024177 | Diosgenin acetate | C29H44O4 | 225768 | 456.66 | 4 | 0 | 5.18 |
| 16 | HBIN024178 | Disogenin,dehydro | C27H40O2 | 587211 | 396.61 | 2 | 0 | 5.71 |
| 17 | HBIN024181 | Diosgenin palmitate | C43H72O4 | 21159048 | 653. 02 | 4 | 0 | 7 |
| 18 | HBIN025419 | Epi-sarsasapogenin | C27H44O3 | 12304430 | 416.64 | 3 | 1 | 5.08 |
| 19 | HBIN025423 | Epistephanine | C37H38N2O6 | 5317122 | 606.71 | 8 | 0 | 3.48 |
| 20 | HBIN028360 | Gracillin | C45H72O17 | 159861 | 885.04 | 17 | 9 | -1.46 |
| 21 | HBIN029305 | Hexahydrofarnesyl acetone | C18H36O | 10408 | 268.48 | 1 | 0 | 4.79 |
| 22 | HBIN031650 | Juncunol | C18H18O | 85926875 | 250.33 | 1 | 1 | 4.12 |
| 23 | HBIN031651 | Juncunone | C18H18O3 | 327720 | 282.33 | 3 | 2 | 2.58 |
| 24 | HBIN031652 | Juncusol | C18H18O2 | 72740 | 266.33 | 2 | 2 | 3.46 |
| 25 | HBIN032343 | Kukoamine A | C28H42N4O6 | 5318865 | 530.66 | 8 | 8 | 0.76 |
| 26 | HBIN034743 | Menthiafolin | C26H36O12 | 76960104 | 540.56 | 12 | 5 | -0.44 |
| 27 | HBIN035672 | Monocrotaline | C16H23NO6 | 9415 | 325.36 | 7 | 2 | 0.24 |
| 28 | HBIN035691 | Mono-p-coumaroyl glyceride | C12H14O5 | 5319874 | 238.24 | 5 | 3 | 0.48 |
| 29 | HBIN037405 | Norvaline | C5H11NO2 | 439575 | 117.15 | 3 | 2 | -2.2 |
| 30 | HBIN040122 | Piscidicacid | C11H12O7 | 120693 | 256.21 | 7 | 5 | -0.6 |
| 31 | HBIN040296 | P-menth-4-en-3-one | C10H16O | 107372 | 152.23 | 1 | 0 | 2.2 |
| 32 | HBIN044218 | Smilagenone | C27H42O3 | 160498 | 414.62 | 3 | 0 | 4.94 |
| 33 | HBIN047103 | Trillin | C33H52O8 | 11827970 | 576.76 | 8 | 4 | 2.41 |
| 34 | HBIN048193 | Diosgenone | C27H40O3 | 10251134 | 412.6 | 3 | 0 | 4.83 |
| 35 | HBIN048520 | Xylan | C5H10O6 | 50909243 | 166.13 | 6 | 5 | -2.73 |
