# Supplementary material for: Diosgenin From Dioscorea Nipponica Rhizoma Against Graves’ Disease—On Network Pharmacology and Experimental Evaluation
Source: Front Pharmacol. 2022 Jan 24;12:806829. doi: 10.3389/fphar.2021.806829 (PMC8819592; doi:10.3389/fphar.2021.806829)
Supplement: Supplementary file 2 [file Table2.docx]

**Table 2.** Information for top 10 pathways

| Pathway ID | Pathway name | Count | PValue |
| --- | --- | --- | --- |
| hsa04151 | PI3K-Akt signaling pathway | 20 | 4.36E-10 |
| hsa04015 | Rap1 signaling pathway | 18 | 1.04E-11 |
| hsa04014 | Ras signaling pathway | 16 | 3.33E-09 |
| hsa05205 | Proteoglycans in cancer | 15 | 6.06E-09 |
| hsa04915 | Estrogen signaling pathway | 14 | 8.30E-12 |
| hsa04510 | Focal adhesion | 13 | 6.29E-07 |
| hsa04068 | FoxO signaling pathway | 12 | 6.05E-08 |
| hsa05218 | Melanoma | 11 | 1.29E-09 |
| hsa04917 | Prolactin signaling pathway | 11 | 1.29E-09 |
| hsa04914 | Progesterone-mediated oocyte maturation | 11 | 9.88E-09 |
