# Supplementary material for: Diosgenin From Dioscorea Nipponica Rhizoma Against Graves’ Disease—On Network Pharmacology and Experimental Evaluation
Source: Front Pharmacol. 2022 Jan 24;12:806829. doi: 10.3389/fphar.2021.806829 (PMC8819592; doi:10.3389/fphar.2021.806829)
Supplement: Supplementary file 3 [file Table5.docx]

**TableS3** Information for GD target after PPI analysis.

| Number | Name | Protein name | Degree | BetweennessCentrality | ClosenessCentrality |
| --- | --- | --- | --- | --- | --- |
| 1 | PIK3R1 | Phosphatidylinositol 4,5-bisphosphate 3-kinase catalytic subunit gamma isoform | 105 | 0.0762 | 0.450472 |
| 2 | STAT3 | Somatostatin | 105 | 0.08714 | 0.463968 |
| 3 | MAPK1 | Mitogen-activated protein kinase kinase kinase 7 | 84 | 0.05303 | 0.447307 |
| 4 | MAPK3 | Mitogen-activated protein kinase 14 | 73 | 0.02853 | 0.443155 |
| 5 | NFKB1 | Neurofibromin | 67 | 0.044 | 0.435741 |
| 6 | SRC | Spectrin alpha chain, non-erythrocytic 1 | 67 | 0.0295 | 0.43908 |
| 7 | AKT1 | RAC-alpha serine/threonine-protein kinase | 65 | 0.04745 | 0.444876 |
| 8 | IL6 | Interleukin-5 | 65 | 0.02922 | 0.422255 |
| 9 | JAK2 | Inositol 1,4,5-trisphosphate receptor type 3 | 59 | 0.01723 | 0.413122 |
| 10 | TNF | Transmembrane protease serine 6 | 58 | 0.02743 | 0.42413 |
| 11 | EGFR | Epidermal growth factor receptor | 57 | 0.02451 | 0.43442 |
| 12 | B2M | Beta-2-microglobulin [Cleaved into: Beta-2-microglobulin form pI 5.3] | 56 | 0.02722 | 0.407539 |
| 13 | EGF | Pro-epidermal growth factor | 56 | 0.01637 | 0.421014 |
| 14 | VEGFA | Vitamin D3 receptor | 56 | 0.0148 | 0.422255 |
| 15 | FN1 | Fibronectin | 55 | 0.0088 | 0.406383 |
| 16 | JUN | Tyrosine-protein kinase JAK2 | 55 | 0.03362 | 0.432779 |
| 17 | STAT6 | Signal transducer and activator of transcription 4 | 53 | 0.02261 | 0.426339 |
| 18 | TP53 | DNA topoisomerase 2-alpha | 53 | 0.04944 | 0.416727 |
| 19 | ITGB1 | Integrin alpha-M | 51 | 0.01742 | 0.406671 |
| 20 | PPBP | Peroxisome proliferator-activated receptor gamma | 51 | 0.01261 | 0.381746 |
| 21 | RAC1 | Parvalbumin alpha | 50 | 0.02844 | 0.420088 |
| 22 | AGT | Angiotensinogen | 48 | 0.01698 | 0.391929 |
| 23 | MAPK14 | Mitogen-activated protein kinase 1 | 48 | 0.03325 | 0.422566 |
| 24 | APOB | Apolipoprotein B-100 | 46 | 0.02259 | 0.395992 |
| 25 | CXCL12 | Stromal cell-derived factor 1 | 46 | 0.0145 | 0.405233 |
| 26 | FGA | Fibrinogen alpha chain | 46 | 0.00537 | 0.395172 |
| 27 | POMC | Purine nucleoside phosphorylase | 46 | 0.02581 | 0.372562 |
| 28 | CXCL1 | Growth-regulated alpha protein | 45 | 0.01055 | 0.391661 |
| 29 | IL10 | Insulin-like growth factor-binding protein 3 | 45 | 0.01171 | 0.384048 |
| 30 | ITGB3 | Integrin beta-2 | 43 | 0.01148 | 0.393274 |
| 31 | F2 | Prothrombin | 42 | 0.01399 | 0.384306 |
| 32 | ALB | Serum albumin | 41 | 0.00759 | 0.391126 |
| 33 | CDC42 | Cell division control protein 42 homolog | 40 | 0.01387 | 0.3949 |
| 34 | CTNNB1 | Catenin beta-1 | 40 | 0.01919 | 0.388738 |
| 35 | CXCL8 | Interleukin-8 | 40 | 0.00902 | 0.398193 |
| 36 | EDN1 | Endothelin-1 | 39 | 0.00743 | 0.402954 |
| 37 | F5 | Coagulation factor V | 39 | 0.00432 | 0.372078 |
| 38 | HGF | Beta-hexosaminidase subunit beta | 39 | 0.00649 | 0.403237 |
| 39 | IL4 | Interleukin-37 | 39 | 0.00791 | 0.396266 |
| 40 | TIMP1 | Thyroid hormone receptor beta | 39 | 0.00291 | 0.381746 |
| 41 | HSP90AA1 | Corticosteroid 11-beta-dehydrogenase isozyme 2 | 38 | 0.0176 | 0.406671 |
| 42 | IGF1 | Interferon gamma receptor 1 | 38 | 0.00548 | 0.395445 |
| 43 | INS | Interleukin-9 | 38 | 0.04286 | 0.405807 |
| 44 | TGFB1 | Protransforming growth factor alpha [Cleaved into: Transforming growth factor alpha | 38 | 0.00801 | 0.399582 |
| 45 | LAMB1 | Neural cell adhesion molecule L1 | 37 | 0.00791 | 0.387949 |
| 46 | PDGFB | Platelet-derived growth factor subunit A | 37 | 0.00421 | 0.397641 |
| 47 | ADRB2 | Beta-2 adrenergic receptor | 36 | 0.01709 | 0.365434 |
| 48 | CXCR4 | Prolactin receptor | 36 | 0.00693 | 0.385081 |
| 49 | HLA-A | Hypoxia-inducible factor 1-alpha | 36 | 0.00554 | 0.353268 |
| 50 | PSMB8 | Presenilin-2 | 36 | 0.01089 | 0.369439 |
| 51 | CASR | Extracellular calcium-sensing receptor | 35 | 0.00268 | 0.35568 |
| 52 | TRAF6 | Thyroid peroxidase | 35 | 0.01452 | 0.384822 |
| 53 | APOA1 | Apolipoprotein A-I | 34 | 0.00769 | 0.373533 |
| 54 | HLA-DRB1 | HLA class II histocompatibility antigen, DR alpha chain | 34 | 0.00716 | 0.349603 |
| 55 | IL1B | Interleukin-1 alpha | 34 | 0.00776 | 0.398193 |
| 56 | NFKBIA | Nuclear factor NF-kappa-B p100 subunit | 34 | 0.0074 | 0.393814 |
| 57 | SPTAN1 | Osteopontin | 34 | 0.02619 | 0.382255 |
| 58 | CD44 | CD44 antigen | 33 | 0.01193 | 0.384564 |
| 59 | CST3 | Cystatin-C | 33 | 0.0046 | 0.367779 |
| 60 | ESR1 | Estrogen receptor | 33 | 0.00911 | 0.41223 |
| 61 | HLA-DRA | HLA class II histocompatibility antigen, DQ beta 1 chain | 33 | 0.00367 | 0.34939 |
| 62 | IL2 | Interleukin-1 receptor antagonist protein | 33 | 0.00888 | 0.407829 |
| 63 | PLG | 1-phosphatidylinositol 4,5-bisphosphate phosphodiesterase gamma-2 | 33 | 0.00294 | 0.361742 |
| 64 | CCR5 | C-C chemokine receptor type 5 | 32 | 0.0053 | 0.37329 |
| 65 | FOS | Proto-oncogene c-Fos | 32 | 0.01049 | 0.404947 |
| 66 | ICAM1 | Basement membrane-specific heparan sulfate proteoglycan core protein | 32 | 0.00658 | 0.374755 |
| 67 | GNAS | Guanine nucleotide-binding protein G | 31 | 0.00886 | 0.345806 |
| 68 | ITGAM | Integrin alpha-L | 31 | 0.01112 | 0.388738 |
| 69 | ITGB2 | Integrin beta-1 | 31 | 0.01063 | 0.387949 |
| 70 | SMAD4 | Antileukoproteinase | 31 | 0.01848 | 0.367308 |
| 71 | SPP1 | Kunitz-type protease inhibitor 2 | 31 | 0.00463 | 0.370155 |
| 72 | VCAM1 | Ubiquitin carboxyl-terminal hydrolase 8 | 31 | 0.006 | 0.379219 |
| 73 | FGF23 | Fibroblast growth factor 23 | 30 | 0.00629 | 0.379219 |
| 74 | IRF1 | Interleukin-1 receptor-associated kinase 1 | 30 | 0.00452 | 0.354579 |
| 75 | MMP9 | Neutrophil collagenase | 30 | 0.00465 | 0.395445 |
| 76 | NR3C1 | Oxysterols receptor LXR-beta | 30 | 0.00969 | 0.398193 |
| 77 | TYK2 | Thioredoxin | 30 | 0.00614 | 0.38534 |
| 78 | VWF | Vimentin | 30 | 0.01032 | 0.380731 |
| 79 | CTSD | Cathepsin D | 29 | 0.01329 | 0.368726 |
| 80 | HLA-B | HLA class I histocompatibility antigen, A-26 alpha chain | 29 | 0.00134 | 0.33045 |
| 81 | HLA-C | HLA class I histocompatibility antigen, B-67 alpha chain | 29 | 0.00134 | 0.33045 |
| 82 | IL13 | Interleukin-12 subunit beta | 29 | 0.00408 | 0.385599 |
| 83 | RXRA | Runt-related transcription factor 2 | 29 | 0.02814 | 0.391393 |
| 84 | CASP8 | Caspase-8 | 28 | 0.0173 | 0.37232 |
| 85 | CCR3 | C-C chemokine receptor type 3 | 28 | 0.00045 | 0.336465 |
| 86 | HLA-DPA1 | HLA class II histocompatibility antigen, DM alpha chain | 28 | 0.00111 | 0.331214 |
| 87 | HLA-DPB1 | HLA class II histocompatibility antigen, DP alpha 1 chain | 28 | 0.00111 | 0.331214 |
| 88 | HLA-DQA1 | HLA class II histocompatibility antigen, DP beta 1 chain | 28 | 0.00111 | 0.331214 |
| 89 | HLA-DQA2 | HLA class II histocompatibility antigen, DQ alpha 1 chain | 28 | 0.00111 | 0.331214 |
| 90 | HLA-DQB1 | HLA class II histocompatibility antigen, DQ alpha 2 chain | 28 | 0.00111 | 0.331214 |
| 91 | HLA-DRB5 | HLA class II histocompatibility antigen, DRB1-13 beta chain | 28 | 0.00111 | 0.331214 |
| 92 | IRF5 | Interferon regulatory factor 1 | 28 | 0.00387 | 0.354141 |
| 93 | PSMB9 | Proteasome subunit beta type-8 | 28 | 0.00486 | 0.3622 |
| 94 | SERPINC1 | P-selectin | 28 | 0.00198 | 0.352833 |
| 95 | TF | Angiopoietin-1 receptor | 28 | 0.00208 | 0.368963 |
| 96 | CCL5 | C-C motif chemokine 5 | 27 | 0.00247 | 0.373777 |
| 97 | HLA-G | HLA class II histocompatibility antigen, DR beta 5 chain | 27 | 0.00089 | 0.330069 |
| 98 | MYC | Interferon-induced GTP-binding protein Mx1 | 27 | 0.00963 | 0.402105 |
| 99 | BMP4 | Bone morphogenetic protein 4 | 26 | 0.00644 | 0.351534 |
| 100 | CCL20 | C-C motif chemokine 20 | 26 | 0.00431 | 0.358349 |
| 101 | CD40LG | CD40 ligand | 26 | 0.00725 | 0.380226 |
| 102 | CD80 | T-lymphocyte activation antigen CD80 | 26 | 0.00709 | 0.391393 |
| 103 | CXCL10 | C-X-C motif chemokine 10 | 26 | 0.00091 | 0.357455 |
| 104 | FGF2 | Fibroblast growth factor 2 | 26 | 0.00387 | 0.380226 |
| 105 | IGFBP3 | Insulin-like growth factor-binding protein 1 | 26 | 0.00308 | 0.372078 |
| 106 | LYZ | Lactotransferrin | 26 | 0.00225 | 0.369439 |
| 107 | RETN | Proto-oncogene tyrosine-protein kinase receptor Ret | 26 | 0.0038 | 0.371836 |
| 108 | CCND1 | G1/S-specific cyclin-D1 | 25 | 0.00674 | 0.385081 |
| 109 | CCR1 | C-C chemokine receptor type 1 | 25 | 0.00064 | 0.35568 |
| 110 | CLTC | Clathrin heavy chain 1 | 25 | 0.00843 | 0.372562 |
| 111 | CSF1 | Macrophage colony-stimulating factor 1 | 25 | 0.00171 | 0.372078 |
| 112 | GNAQ | Guanine nucleotide-binding protein G | 25 | 0.00326 | 0.359248 |
| 113 | HDAC1 | Granzyme B | 25 | 0.00998 | 0.364504 |
| 114 | IGF2 | Insulin-like growth factor 1 receptor | 25 | 0.00109 | 0.361059 |
| 115 | NCAM1 | Nicotinamide phosphoribosyltransferase | 25 | 0.00639 | 0.31851 |
| 116 | SOCS3 | Alpha-synuclein | 25 | 0.02229 | 0.355459 |
| 117 | SST | Proto-oncogene tyrosine-protein kinase Src | 25 | 0.00433 | 0.338853 |
| 118 | BIRC3 | Baculoviral IAP repeat-containing protein 3 | 24 | 0.0022 | 0.348117 |
| 119 | C4A | Complement C4-A | 24 | 0.00965 | 0.346224 |
| 120 | CD28 | T-cell-specific surface glycoprotein CD28 | 24 | 0.00689 | 0.377719 |
| 121 | CXCR3 | C-X-C chemokine receptor type 3 | 24 | 0.00068 | 0.331597 |
| 122 | ELANE | Neutrophil elastase | 24 | 0.00238 | 0.36381 |
| 123 | F13A1 | Coagulation factor XIII A chain | 24 | 0.00055 | 0.361971 |
| 124 | SDC1 | Sodium channel protein type 5 subunit alpha | 24 | 0.00364 | 0.364272 |
| 125 | SERPINE1 | Antithrombin-III | 24 | 0.00303 | 0.366837 |
| 126 | TNFRSF1B | Tumor necrosis factor receptor superfamily member 1A | 24 | 0.00823 | 0.361971 |
| 127 | CCR4 | C-C chemokine receptor type 4 | 23 | 0.0007 | 0.327803 |
| 128 | CXCL11 | C-X-C motif chemokine 11 | 23 | 0.00026 | 0.333916 |
| 129 | ERBB2 | Receptor tyrosine-protein kinase erbB-2 | 23 | 0.00256 | 0.389531 |
| 130 | HSPG2 | Heat shock cognate 71 kDa protein | 23 | 0.01068 | 0.366837 |
| 131 | IFNG | Interferon beta | 23 | 0.00421 | 0.382 |
| 132 | IGFBP1 | Insulin-like growth factor II | 23 | 0.00041 | 0.35305 |
| 133 | PTH | Prostaglandin G/H synthase 2 | 23 | 0.00563 | 0.324646 |
| 134 | RUNX1 | Nuclear receptor ROR-gamma | 23 | 0.00943 | 0.358349 |
| 135 | TNFSF11 | Tumor necrosis factor ligand superfamily member 10 | 23 | 0.01448 | 0.379219 |
| 136 | BPI | Bactericidal permeability-increasing protein | 22 | 0.00133 | 0.359473 |
| 137 | CIITA | MHC class II transactivator | 22 | 0.00036 | 0.324278 |
| 138 | CTLA4 | Cytotoxic T-lymphocyte protein 4 | 22 | 0.00706 | 0.359925 |
| 139 | CXCL9 | C-X-C motif chemokine 9 | 22 | 0 | 0.327616 |
| 140 | CXCR2 | C-X-C chemokine receptor type 2 | 22 | 0 | 0.327616 |
| 141 | CXCR5 | C-X-C chemokine receptor type 5 | 22 | 0 | 0.327616 |
| 142 | GAL | Galanin peptides | 22 | 0 | 0.327616 |
| 143 | HSPA8 | Endoplasmic reticulum chaperone BiP | 22 | 0.01675 | 0.370155 |
| 144 | IRF8 | Interferon regulatory factor 5 | 22 | 0.0011 | 0.333527 |
| 145 | PSAP | Myeloblastin | 22 | 0 | 0.327616 |
| 146 | VEGFC | Vascular endothelial growth factor A | 22 | 0.0005 | 0.364968 |
| 147 | CP | Ceruloplasmin | 21 | 0.0004 | 0.347273 |
| 148 | CSF2 | Granulocyte-macrophage colony-stimulating factor | 21 | 0.00137 | 0.385599 |
| 149 | HIF1A | Hepatocyte growth factor | 21 | 0.00195 | 0.388738 |
| 150 | HP | High mobility group protein B1 | 21 | 0.00443 | 0.355239 |
| 151 | MAP3K7 | Membrane-associated guanylate kinase, WW and PDZ domain-containing protein 2 | 21 | 0.00399 | 0.353486 |
| 152 | RAF1 | Ras-related C3 botulinum toxin substrate 2 | 21 | 0.0104 | 0.386379 |
| 153 | STAT4 | Signal transducer and activator of transcription 3 | 21 | 0.00668 | 0.364504 |
| 154 | TRIM31 | Thyrotropin-releasing hormone receptor | 21 | 0.00003 | 0.319042 |
| 155 | WFS1 | von Willebrand factor | 21 | 0.0006 | 0.345806 |
| 156 | CCL2 | C-C motif chemokine 2 | 20 | 0.00082 | 0.376726 |
| 157 | EDNRA | Endothelin-1 receptor | 20 | 0.00131 | 0.369439 |
| 158 | GNAO1 | Guanine nucleotide-binding protein subunit alpha-11 | 20 | 0.00439 | 0.375984 |
| 159 | IL2RA | Interleukin-23 receptor | 20 | 0.00304 | 0.391393 |
| 160 | TAB2 | Synaptophysin | 20 | 0.00295 | 0.351318 |
| 161 | TLR4 | Toll-like receptor 3 | 20 | 0.00823 | 0.371595 |
| 162 | EZR | Ezrin | 19 | 0.00342 | 0.370155 |
| 163 | HMGB1 | High mobility group protein HMGI-C | 19 | 0.00469 | 0.369677 |
| 164 | IRAK1 | IQ calmodulin-binding motif-containing protein 1 | 19 | 0.00355 | 0.333916 |
| 165 | LTF | Lymphotoxin-alpha | 19 | 0.00032 | 0.353268 |
| 166 | MET | Methyl-CpG-binding protein 2 | 19 | 0.00092 | 0.381746 |
| 167 | MMP8 | Matrilysin | 19 | 0.00026 | 0.351318 |
| 168 | NOTCH1 | Nitric oxide synthase, endothelial | 19 | 0.00661 | 0.35436 |
| 169 | PPARA | POU domain, class 5, transcription factor 1 | 19 | 0.01255 | 0.381491 |
| 170 | PPARG | Peroxisome proliferator-activated receptor alpha | 19 | 0.01475 | 0.371836 |
| 171 | SERPINF2 | Plasminogen activator inhibitor 1 | 19 | 0.00018 | 0.345181 |
| 172 | TSHR | Thyrotropin subunit beta | 19 | 0.00132 | 0.303818 |
| 173 | CALCA | Calcitonin gene-related peptide 1 | 18 | 0.00104 | 0.305926 |
| 174 | CD86 | T-lymphocyte activation antigen CD86 | 18 | 0.00403 | 0.381491 |
| 175 | GATA3 | Trans-acting T-cell-specific transcription factor GATA-3 | 18 | 0.00231 | 0.361742 |
| 176 | GNA11 | Glutaminase kidney isoform, mitochondrial | 18 | 0.00058 | 0.351103 |
| 177 | IL12B | Interleukin-12 subunit alpha | 18 | 0.00062 | 0.368252 |
| 178 | IL17A | Interleukin-15 | 18 | 0.00381 | 0.360377 |
| 179 | IL1A | Interleukin-18 receptor accessory protein | 18 | 0.00227 | 0.372562 |
| 180 | IL2RB | Interleukin-2 receptor subunit alpha | 18 | 0.00278 | 0.382766 |
| 181 | PLAUR | Tissue-type plasminogen activator | 18 | 0.00496 | 0.361514 |
| 182 | PRKAR1A | Prickle-like protein 1 | 18 | 0.00713 | 0.364504 |
| 183 | PTPRC | Tyrosine-protein phosphatase non-receptor type 3 | 18 | 0.00372 | 0.335677 |
| 184 | PTX3 | 6-pyruvoyl tetrahydrobiopterin synthase | 18 | 0.00008 | 0.350673 |
| 185 | TIMP2 | Metalloproteinase inhibitor 1 | 18 | 0.00008 | 0.350673 |
| 186 | ADCYAP1 | Pituitary adenylate cyclase-activating polypeptide | 17 | 0 | 0.295666 |
| 187 | ADM | ADM [Cleaved into: Adrenomedullin | 17 | 0 | 0.295666 |
| 188 | ADRB1 | Beta-1 adrenergic receptor | 17 | 0 | 0.295666 |
| 189 | CALCR | Calcitonin receptor | 17 | 0 | 0.295666 |
| 190 | CD36 | Platelet glycoprotein 4 | 17 | 0.00684 | 0.358573 |
| 191 | CGA | Glycoprotein hormones alpha chain | 17 | 0 | 0.295666 |
| 192 | FSHR | Follicle-stimulating hormone receptor | 17 | 0 | 0.295666 |
| 193 | HRH2 | Hypoxanthine-guanine phosphoribosyltransferase | 17 | 0 | 0.295666 |
| 194 | INSL3 | Insulin | 17 | 0 | 0.295666 |
| 195 | KCNAB2 | Potassium voltage-gated channel subfamily A member 4 | 17 | 0.02603 | 0.307899 |
| 196 | LEP | Low-density lipoprotein receptor | 17 | 0.00527 | 0.382766 |
| 197 | LHCGR | Galectin-3 | 17 | 0 | 0.295666 |
| 198 | MC2R | Mannose-binding protein C | 17 | 0 | 0.295666 |
| 199 | NFKB2 | Nuclear factor NF-kappa-B p105 subunit | 17 | 0.00083 | 0.343731 |
| 200 | PTHLH | Parathyroid hormone | 17 | 0 | 0.295666 |
| 201 | PTPN2 | Tyrosine-protein phosphatase non-receptor type 12 | 17 | 0.00114 | 0.367543 |
| 202 | TSHB | E3 ubiquitin-protein ligase TRIM31 | 17 | 0 | 0.295666 |
| 203 | FOXP3 | Forkhead box protein P3 | 16 | 0.00091 | 0.356343 |
| 204 | HDAC2 | Histone deacetylase 1 | 16 | 0.00663 | 0.355901 |
| 205 | IL18 | Interleukin-17 receptor A | 16 | 0.00156 | 0.366603 |
| 206 | KALRN | Transcription factor AP-1 | 16 | 0.00053 | 0.323912 |
| 207 | NOS2 | Nucleotide-binding oligomerization domain-containing protein 2 | 16 | 0.00167 | 0.371595 |
| 208 | PRKCB | cAMP-dependent protein kinase type I-alpha regulatory subunit | 16 | 0.00269 | 0.364504 |
| 209 | PTEN | Proteasome subunit beta type-9 | 16 | 0.00237 | 0.371114 |
| 210 | TNFRSF1A | Tumor necrosis factor receptor superfamily member 13C | 16 | 0.00162 | 0.361742 |
| 211 | BCL2 | Apoptosis regulator Bcl-2 | 15 | 0.00365 | 0.373777 |
| 212 | HSPA1A | Heat shock protein HSP 90-alpha | 15 | 0.00203 | 0.35568 |
| 213 | IGF1R | Insulin-like growth factor I | 15 | 0.00196 | 0.37451 |
| 214 | ITGA4 | Interferon regulatory factor 8 | 15 | 0.00062 | 0.347483 |
| 215 | ITGAL | Integrin alpha-6 | 15 | 0.00178 | 0.336861 |
| 216 | NEU1 | Nebulin | 15 | 0 | 0.339656 |
| 217 | NGF | NF-kappa-B inhibitor alpha | 15 | 0.00291 | 0.385081 |
| 218 | SIRT1 | Leucine-rich repeat protein SHOC-2 | 15 | 0.00385 | 0.354579 |
| 219 | SLPI | Slit homolog 2 protein | 15 | 0 | 0.339656 |
| 220 | AKT2 | RAC-beta serine/threonine-protein kinase | 14 | 0.00159 | 0.366368 |
| 221 | CD40 | Tumor necrosis factor receptor superfamily member 5 | 14 | 0.00052 | 0.370634 |
| 222 | CDKN1B | Cyclin-dependent kinase inhibitor 1B | 14 | 0.00179 | 0.360377 |
| 223 | EPO | Erythropoietin | 14 | 0.00166 | 0.356343 |
| 224 | ERBB3 | Receptor tyrosine-protein kinase erbB-3 | 14 | 0.00026 | 0.356565 |
| 225 | GAST | Gastrin [Cleaved into: Gastrin-71 | 14 | 0.00014 | 0.335677 |
| 226 | GNRH1 | Protein ALEX | 14 | 0.00011 | 0.335873 |
| 227 | HSPA5 | Heat shock 70 kDa protein 4 | 14 | 0.00179 | 0.326124 |
| 228 | LRP2 | Lipoprotein lipase | 14 | 0.0057 | 0.347694 |
| 229 | TNFRSF11A | Tumor necrosis factor alpha-induced protein 3 | 14 | 0.0002 | 0.332753 |
| 230 | TNFSF12 | Tumor necrosis factor ligand superfamily member 11 | 14 | 0.00092 | 0.330069 |
| 231 | TRH | TNF receptor-associated factor 6 | 14 | 0.00011 | 0.335873 |
| 232 | CD59 | CD59 glycoprotein | 13 | 0.00279 | 0.336663 |
| 233 | ERBB4 | Receptor tyrosine-protein kinase erbB-4 | 13 | 0.00073 | 0.359925 |
| 234 | FGFR3 | Fibroblast growth factor receptor 3 | 13 | 0.00042 | 0.37451 |
| 235 | IL12A | Interleukin-11 | 13 | 0.00011 | 0.337854 |
| 236 | IL5 | Interleukin-4 receptor subunit alpha | 13 | 0.00024 | 0.340463 |
| 237 | LTA | Low-density lipoprotein receptor-related protein 2 | 13 | 0.00003 | 0.325383 |
| 238 | MLN | Proliferation marker protein Ki-67 | 13 | 0.00002 | 0.334696 |
| 239 | PLCG2 | Urokinase plasminogen activator surface receptor | 13 | 0.0018 | 0.360377 |
| 240 | TNFSF13B | Tumor necrosis factor ligand superfamily member 12 | 13 | 0.00006 | 0.327242 |
| 241 | TRHR | Pro-thyrotropin-releasing hormone | 13 | 0.00002 | 0.334696 |
| 242 | WNT5A | Serine/threonine-protein kinase WNK4 | 13 | 0.00075 | 0.350031 |
| 243 | BCL2L1 | Bcl-2-like protein 1 | 12 | 0.00259 | 0.364504 |
| 244 | CD274 | Programmed cell death 1 ligand 1 | 12 | 0.00041 | 0.305274 |
| 245 | ESR2 | Estrogen receptor beta | 12 | 0.00094 | 0.360151 |
| 246 | IL23R | Interleukin-23 subunit alpha | 12 | 0.0003 | 0.370394 |
| 247 | NOS3 | Nitric oxide synthase | 12 | 0.00049 | 0.357455 |
| 248 | RARA | RAF proto-oncogene serine/threonine-protein kinase | 12 | 0.00124 | 0.348965 |
| 249 | TNFRSF13C | Tumor necrosis factor receptor superfamily member 11B | 12 | 0 | 0.325199 |
| 250 | TTR | Thyrotropin receptor | 12 | 0.00042 | 0.334501 |
| 251 | CALR | Calreticulin | 11 | 0.00119 | 0.321188 |
| 252 | CSNK2B | Casein kinase II subunit beta | 11 | 0.0022 | 0.334892 |
| 253 | EPAS1 | Endothelial PAS domain-containing protein 1 | 11 | 0.00138 | 0.335677 |
| 254 | FAS | Tumor necrosis factor receptor superfamily member 6 | 11 | 0.0012 | 0.357455 |
| 255 | HEXB | Beta-hexosaminidase subunit alpha | 11 | 0.00349 | 0.322091 |
| 256 | HLA-DMA | HLA class I histocompatibility antigen, Cw-8 alpha chain | 11 | 0 | 0.272727 |
| 257 | IFNB1 | Interferon alpha-2 | 11 | 0.00105 | 0.331405 |
| 258 | IL23A | Interleukin-22 | 11 | 0.00026 | 0.363118 |
| 259 | IL6R | Interleukin-6 | 11 | 0.00031 | 0.361514 |
| 260 | ITGA6 | Integrin alpha-4 | 11 | 0.00006 | 0.35568 |
| 261 | ITGB4 | Integrin beta-3 | 11 | 0.00012 | 0.354141 |
| 262 | KIT | Killer cell immunoglobulin-like receptor 3DL1 | 11 | 0.00085 | 0.363579 |
| 263 | KITLG | Mast/stem cell growth factor receptor Kit | 11 | 0.00119 | 0.351318 |
| 264 | LDLR | Laminin subunit beta-1 | 11 | 0.00053 | 0.341885 |
| 265 | MPO | Matrix metalloproteinase-9 | 11 | 0.00015 | 0.327055 |
| 266 | PDGFRA | Platelet-derived growth factor subunit B | 11 | 0.0003 | 0.357009 |
| 267 | PGR | Placenta growth factor | 11 | 0.00039 | 0.344144 |
| 268 | PRL | cGMP-dependent protein kinase 1 | 11 | 0.00106 | 0.355459 |
| 269 | RARB | Retinoic acid receptor alpha | 11 | 0.00112 | 0.346014 |
| 270 | SMARCB1 | Mothers against decapentaplegic homolog 4 | 11 | 0.00151 | 0.332946 |
| 271 | ADAM17 | Disintegrin and metalloproteinase domain-containing protein 17 | 10 | 0.00273 | 0.344144 |
| 272 | BLK | Tyrosine-protein kinase Blk | 10 | 0.00107 | 0.358349 |
| 273 | BMPR2 | Bone morphogenetic protein receptor type-2 | 10 | 0.00241 | 0.304463 |
| 274 | CAT | Catalase | 10 | 0.00209 | 0.314662 |
| 275 | CDKN2A | Tumor suppressor ARF | 10 | 0.00059 | 0.343319 |
| 276 | CYB5R3 | NADH-cytochrome b5 reductase 3 | 10 | 0 | 0.32191 |
| 277 | ETS1 | Protein C-ets-1 | 10 | 0.00039 | 0.352615 |
| 278 | FASLG | Tumor necrosis factor ligand superfamily member 6 | 10 | 0.00053 | 0.360604 |
| 279 | IL1R1 | Interleukin-1 beta | 10 | 0.00043 | 0.361286 |
| 280 | IL3 | Interleukin-2 receptor subunit beta | 10 | 0.00014 | 0.332946 |
| 281 | IL4R | Interleukin-4 | 10 | 0.00014 | 0.351966 |
| 282 | IL6ST | Interleukin-6 receptor subunit alpha | 10 | 0.00047 | 0.339455 |
| 283 | L1CAM | Keratin, type II cytoskeletal 5 | 10 | 0.00122 | 0.355459 |
| 284 | LPL | Apolipoprotein | 10 | 0.00475 | 0.334306 |
| 285 | MMP3 | Neprilysin | 10 | 0.00044 | 0.35436 |
| 286 | MX1 | Mucin-1 | 10 | 0.0005 | 0.3295 |
| 287 | PDCD1 | Pericentrin | 10 | 0.00012 | 0.282963 |
| 288 | PIK3CG | Progesterone receptor | 10 | 0.00034 | 0.359473 |
| 289 | PRTN3 | Homeobox protein prophet of Pit-1 | 10 | 0 | 0.32191 |
| 290 | RNASET2 | Rho-related GTP-binding protein RhoH | 10 | 0 | 0.32191 |
| 291 | TAP1 | TGF-beta-activated kinase 1 and MAP3K7-binding protein 2 | 10 | 0.00029 | 0.311244 |
| 292 | TEK | T-box transcription factor TBX21 | 10 | 0.00025 | 0.356122 |
| 293 | TLR3 | Toll-like receptor 1 | 10 | 0.00125 | 0.325383 |
| 294 | TXN | Transthyretin | 10 | 0.00455 | 0.33607 |
| 295 | YAP1 | DNA repair protein XRCC1 | 10 | 0.00132 | 0.349603 |
| 296 | ADIPOQ | Adiponectin | 9 | 0.00044 | 0.348752 |
| 297 | CYP1A1 | Cytochrome P450 1A1 | 9 | 0.01587 | 0.289394 |
| 298 | ENG | Endoglin | 9 | 0.00163 | 0.340666 |
| 299 | FGFR4 | Fibroblast growth factor receptor 4 | 9 | 0.00075 | 0.351318 |
| 300 | GLI3 | Gap junction alpha-1 protein | 9 | 0.00399 | 0.318865 |
| 301 | MMP7 | Stromelysin-1 | 9 | 0.0003 | 0.332559 |
| 302 | NOTCH4 | Neurogenic locus notch homolog protein 2 | 9 | 0.00167 | 0.322999 |
| 303 | PTGS2 | Phosphatidylinositol 3,4,5-trisphosphate 3-phosphatase and dual-specificity protein phosphatase | 9 | 0.00012 | 0.361286 |
| 304 | RAC2 | Ras-related C3 botulinum toxin substrate 1 | 9 | 0.00054 | 0.33548 |
| 305 | RUNX2 | Runt-related transcription factor 1 | 9 | 0.00191 | 0.339053 |
| 306 | S100B | Protein S100-A9 | 9 | 0.00034 | 0.336268 |
| 307 | AGER | Advanced glycosylation end product-specific receptor | 8 | 0.00015 | 0.340261 |
| 308 | APC | Adenomatous polyposis coli protein | 8 | 0.00047 | 0.328555 |
| 309 | BMPR1A | Bone morphogenetic protein receptor type-1A | 8 | 0.00147 | 0.295057 |
| 310 | CD79A | B-cell antigen receptor complex-associated protein alpha chain | 8 | 0.00446 | 0.326868 |
| 311 | FCGR2A | Low affinity immunoglobulin gamma Fc region receptor II-a | 8 | 0.00032 | 0.337059 |
| 312 | GH1 | Glutathione hydrolase 1 proenzyme | 8 | 0.00032 | 0.358125 |
| 313 | HSPA1L | Heat shock 70 kDa protein 1A | 8 | 0.00011 | 0.315529 |
| 314 | IFIH1 | Indoleamine 2,3-dioxygenase 1 | 8 | 0.00033 | 0.323181 |
| 315 | IL15 | Interleukin-13 | 8 | 0.0105 | 0.326682 |
| 316 | LPA | Leiomodin-1 | 8 | 0.00047 | 0.310569 |
| 317 | MYLK | Myosin-11 | 8 | 0.00382 | 0.333722 |
| 318 | PTPN22 | Tyrosine-protein phosphatase non-receptor type 2 | 8 | 0 | 0.259982 |
| 319 | SOD1 | Suppressor of cytokine signaling 3 | 8 | 0.00111 | 0.315702 |
| 320 | SOD2 | Superoxide dismutase [Cu-Zn] | 8 | 0.00209 | 0.317804 |
| 321 | TAP2 | Antigen peptide transporter 1 | 8 | 0 | 0.297353 |
| 322 | TGFA | Thyroglobulin | 8 | 0.00022 | 0.338053 |
| 323 | TGFBR1 | Transforming growth factor beta-1 proprotein [Cleaved into: Latency-associated peptide | 8 | 0.0002 | 0.31922 |
| 324 | TNFAIP3 | Tumor necrosis factor | 8 | 0.00004 | 0.3164 |
| 325 | ANGPT1 | Angiopoietin-1 | 7 | 0.0001 | 0.353486 |
| 326 | COL11A2 | Collagen alpha-2 | 7 | 0.00002 | 0.316575 |
| 327 | COL1A1 | Collagen alpha-1 | 7 | 0.00002 | 0.316575 |
| 328 | COL3A1 | Collagen alpha-1 | 7 | 0.00002 | 0.316575 |
| 329 | COL5A1 | Collagen alpha-1 | 7 | 0.00002 | 0.316575 |
| 330 | CRP | C-reactive protein [Cleaved into: C-reactive protein | 7 | 0.00127 | 0.327991 |
| 331 | FGF1 | Fibroblast growth factor 1 | 7 | 0.00001 | 0.342294 |
| 332 | FLT1 | Vascular endothelial growth factor receptor 1 | 7 | 0.00004 | 0.333527 |
| 333 | FXYD2 | Sodium/potassium-transporting ATPase subunit gamma | 7 | 0.01308 | 0.305926 |
| 334 | IL18R1 | Interleukin-18 | 7 | 0.00023 | 0.345181 |
| 335 | ITPR3 | Integrin beta-4 | 7 | 0.00393 | 0.315355 |
| 336 | NOD2 | NACHT, LRR and PYD domains-containing protein 1 | 7 | 0.0005 | 0.313457 |
| 337 | NOTCH2 | Neurogenic locus notch homolog protein 1 | 7 | 0.00042 | 0.276411 |
| 338 | PECAM1 | Platelet-derived growth factor receptor alpha | 7 | 0.00033 | 0.345806 |
| 339 | SLIT2 | Solute carrier family 2, facilitated glucose transporter member 1 | 7 | 0.01392 | 0.311752 |
| 340 | TNFSF10 | Tumor necrosis factor receptor superfamily member 4 | 7 | 0.00121 | 0.337655 |
| 341 | VDR | Vascular cell adhesion protein 1 | 7 | 0.00152 | 0.302056 |
| 342 | VIM | Vascular endothelial growth factor C | 7 | 0.01396 | 0.343114 |
| 343 | ACTA2 | Alpha-actin-2 | 6 | 0.00037 | 0.276812 |
| 344 | APEX1 | Apurinic-apyrimidinic endonuclease 1) | 6 | 0.00608 | 0.301104 |
| 345 | APOH | Beta-2-glycoprotein 1 | 6 | 0.00017 | 0.315008 |
| 346 | BAX | Apoptosis regulator BAX | 6 | 0.00006 | 0.302056 |
| 347 | CSF3 | Granulocyte colony-stimulating factor | 6 | 0.00006 | 0.337257 |
| 348 | CYFIP2 | Cytoplasmic FMR1-interacting protein 2 | 6 | 0.00007 | 0.330831 |
| 349 | DNMT1 | DNA methyltransferase HsaI | 6 | 0.00651 | 0.305274 |
| 350 | DNMT3A | DNA MTase HsaIIIA | 6 | 0.00608 | 0.29643 |
| 351 | GSR | Glutathione peroxidase 1 | 6 | 0.00161 | 0.261048 |
| 352 | HSPA4 | Heat shock 70 kDa protein 1-like | 6 | 0.00019 | 0.306417 |
| 353 | IFNGR1 | Interferon gamma | 6 | 0.00015 | 0.330831 |
| 354 | IL1RN | Interleukin-1 receptor type 1 | 6 | 0.00007 | 0.325568 |
| 355 | IL37 | Interleukin-33 | 6 | 0.0002 | 0.321729 |
| 356 | MYH11 | Myc proto-oncogene protein | 6 | 0.00101 | 0.307899 |
| 357 | NKX2-5 | Homeobox protein Nkx-2.1 | 6 | 0.00121 | 0.294905 |
| 358 | PDGFA | Programmed cell death protein 1 | 6 | 0.00007 | 0.327991 |
| 359 | PLAT | Platelet-activating factor acetylhydrolase | 6 | 0.00009 | 0.315877 |
| 360 | POU5F1 | Pituitary-specific positive transcription factor 1 | 6 | 0.00366 | 0.337456 |
| 361 | PTPN12 | Parathyroid hormone-related protein | 6 | 0.00009 | 0.31449 |
| 362 | RHOH | Resistin | 6 | 0.00002 | 0.300787 |
| 363 | TBX21 | T-box transcription factor TBX19 | 6 | 0.00008 | 0.308396 |
| 364 | TLR6 | Toll-like receptor 5 | 6 | 0.00069 | 0.318865 |
| 365 | CD55 | Complement decay-accelerating factor | 5 | 0.00018 | 0.312773 |
| 366 | CETP | Cholesteryl ester transfer protein | 5 | 0.00001 | 0.291455 |
| 367 | COG2 | Conserved oligomeric Golgi complex subunit 2 | 5 | 0.00012 | 0.319042 |
| 368 | DCC | Netrin receptor DCC | 5 | 0.00018 | 0.318687 |
| 369 | DNMT3B | DNA | 5 | 0.00163 | 0.274032 |
| 370 | F3 | Tissue factor | 5 | 0.00002 | 0.296124 |
| 371 | GAD1 | Glutamate decarboxylase 1 | 5 | 0.00332 | 0.274294 |
| 372 | GFRA1 | Protein names | 5 | 0.00045 | 0.336465 |
| 373 | GJA1 | Appetite-regulating hormone | 5 | 0.00004 | 0.337854 |
| 374 | GPX1 | Alanine aminotransferase 1 | 5 | 0.00001 | 0.243415 |
| 375 | HSD11B1 | Histamine H2 receptor | 5 | 0.00883 | 0.293846 |
| 376 | IL17F | Interleukin-17A | 5 | 0.00072 | 0.320112 |
| 377 | IL18RAP | Interleukin-18 receptor 1 | 5 | 0.00006 | 0.312432 |
| 378 | IL7 | Interleukin-6 receptor subunit beta | 5 | 0.00021 | 0.326682 |
| 379 | KLHL3 | Kit ligand | 5 | 0.01126 | 0.264421 |
| 380 | NF1 | Sialidase-1 | 5 | 0.00005 | 0.297662 |
| 381 | PSEN2 | Prosaposin | 5 | 0.00025 | 0.30142 |
| 382 | RET | Renin | 5 | 0.00349 | 0.331597 |
| 383 | SH2B3 | Alpha-2-antiplasmin | 5 | 0.00003 | 0.303335 |
| 384 | SNCA | SWI/SNF-related matrix-associated actin-dependent regulator of chromatin subfamily B member 1 | 5 | 0.00128 | 0.320112 |
| 385 | SOD3 | Superoxide dismutase [Mn], mitochondrial | 5 | 0.00062 | 0.310233 |
| 386 | THRA | Thrombomodulin | 5 | 0.00013 | 0.32631 |
| 387 | THRB | Thyroid hormone receptor alpha | 5 | 0.00013 | 0.32631 |
| 388 | USP8 | Tyrosinase | 5 | 0.00002 | 0.329689 |
| 389 | AHCY | Adenosylhomocysteinase | 4 | 0.01043 | 0.238254 |
| 390 | ANGPT2 | Angiopoietin-2 | 4 | 0.00001 | 0.329121 |
| 391 | ARHGEF2 | Rho guanine nucleotide exchange factor 2 | 4 | 0 | 0.297353 |
| 392 | ATF6 | Cyclic AMP-dependent transcription factor ATF-6 alpha | 4 | 0.00022 | 0.310738 |
| 393 | ATP1A1 | Sodium/potassium-transporting ATPase subunit alpha-1 | 4 | 0.00005 | 0.238155 |
| 394 | ATP1A2 | Sodium/potassium-transporting ATPase subunit alpha-2 | 4 | 0.00005 | 0.238155 |
| 395 | ATP1B1 | Sodium/potassium-transporting ATPase subunit beta-1 | 4 | 0 | 0.234644 |
| 396 | ATP1B2 | Sodium/potassium-transporting ATPase subunit beta-2 | 4 | 0 | 0.234644 |
| 397 | ATP1B4 | Protein ATP1B4 | 4 | 0.00124 | 0.270028 |
| 398 | BAK1 | Bcl-2 homologous antagonist/killer | 4 | 0 | 0.297353 |
| 399 | BGLAP | Osteocalcin | 4 | 0.00022 | 0.298127 |
| 400 | CACNA1D | Voltage-dependent L-type calcium channel subunit alpha-1D | 4 | 0.00167 | 0.298749 |
| 401 | CACNB4 | Voltage-dependent L-type calcium channel subunit beta-4 | 4 | 0.00013 | 0.25776 |
| 402 | CD69 | Early activation antigen CD69 | 4 | 0.00024 | 0.300314 |
| 403 | CEP57 | Centrosomal protein of 57 kDa | 4 | 0.00009 | 0.296584 |
| 404 | DLL4 | Delta-like protein 4 | 4 | 0 | 0.274163 |
| 405 | F9 | Coagulation factor IX | 4 | 0.00001 | 0.293395 |
| 406 | GAD2 | Glutamate decarboxylase 2 | 4 | 0.00262 | 0.271951 |
| 407 | GGT1 | GDNF family receptor alpha-1 | 4 | 0.00029 | 0.226303 |
| 408 | IFNA2 | Interferon-induced helicase C domain-containing protein 1 | 4 | 0.00007 | 0.297662 |
| 409 | IL33 | Interleukin-3 | 4 | 0.00006 | 0.307239 |
| 410 | IL9 | Interleukin-7 | 4 | 0.00001 | 0.321368 |
| 411 | KDM6A | Potassium voltage-gated channel subfamily V member 2 | 4 | 0.00002 | 0.310065 |
| 412 | KIR3DL1 | Lysine-specific demethylase 6A | 4 | 0 | 0.292795 |
| 413 | LIPC | LIM/homeobox protein Lhx4 | 4 | 0.00001 | 0.290421 |
| 414 | MUC1 | Methionine synthase reductase | 4 | 0 | 0.331214 |
| 415 | OBSCN | Glucocorticoid receptor | 4 | 0 | 0.297353 |
| 416 | PARP1 | N-glycosylase/DNA lyase [Includes: 8-oxoguanine DNA glycosylase | 4 | 0.00115 | 0.279922 |
| 417 | PGF | Platelet endothelial cell adhesion molecule | 4 | 0 | 0.320291 |
| 418 | PNP | Plasminogen | 4 | 0.01045 | 0.184898 |
| 419 | PRDX1 | Platelet basic protein | 4 | 0.00008 | 0.29953 |
| 420 | RORC | E3 ubiquitin-protein ligase RNF213 | 4 | 0 | 0.320829 |
| 421 | SCN1B | Sodium channel protein type 1 subunit alpha | 4 | 0.00001 | 0.277079 |
| 422 | SELE | Succinate dehydrogenase [ubiquinone] iron-sulfur subunit, mitochondrial | 4 | 0.00001 | 0.315355 |
| 423 | SELP | L-selectin | 4 | 0.00009 | 0.303818 |
| 424 | STK39 | Signal transducer and activator of transcription 6 | 4 | 0.00607 | 0.283804 |
| 425 | TLR7 | Toll-like receptor 6 | 4 | 0 | 0.284508 |
| 426 | TLR8 | Toll-like receptor 7 | 4 | 0 | 0.284508 |
| 427 | TNNI3 | Tumor necrosis factor ligand superfamily member 4 | 4 | 0.00174 | 0.256032 |
| 428 | TNNT2 | Troponin I, cardiac muscle | 4 | 0.00174 | 0.256032 |
| 429 | TP63 | Cellular tumor antigen p53 | 4 | 0.00002 | 0.30142 |
| 430 | TPO | Tumor protein 63 | 4 | 0.00702 | 0.274688 |
| 431 | ACVR1 | Activin receptor type-1 | 3 | 0 | 0.265032 |
| 432 | BMP6 | Bone morphogenetic protein 6 | 3 | 0.0001 | 0.243312 |
| 433 | CACNA1A | Voltage-dependent P/Q-type calcium channel subunit alpha-1A | 3 | 0.00107 | 0.303979 |
| 434 | CACNA1S | Voltage-dependent L-type calcium channel subunit alpha-1S | 3 | 0 | 0.250109 |
| 435 | CRLF1 | Cytokine receptor-like factor 1 | 3 | 0 | 0.293846 |
| 436 | CS | Citrate synthase, mitochondrial | 3 | 0.66667 | 1 |
| 437 | CYP27B1 | 25-hydroxyvitamin D-1 alpha hydroxylase, mitochondrial | 3 | 0.00016 | 0.290274 |
| 438 | CYP7A1 | Cytochrome P450 7A1 | 3 | 0 | 0.287795 |
| 439 | DDC | Aromatic-L-amino-acid decarboxylase | 3 | 0.00035 | 0.218452 |
| 440 | DPP4 | Dipeptidyl peptidase 4 | 3 | 0.01753 | 0.291011 |
| 441 | FCGR3B | Low affinity immunoglobulin gamma Fc region receptor III-B | 3 | 0.00349 | 0.316225 |
| 442 | FLT3 | Receptor-type tyrosine-protein kinase FLT3 | 3 | 0 | 0.338253 |
| 443 | G6PD | Glucose-6-phosphate 1-dehydrogenase | 3 | 0.00045 | 0.296891 |
| 444 | GLS | Transcriptional activator GLI3 | 3 | 0 | 0.216963 |
| 445 | GZMB | Glutathione S-transferase P | 3 | 0.00362 | 0.274032 |
| 446 | IL11 | Interleukin-10 | 3 | 0 | 0.293846 |
| 447 | IL22 | Interleukin-21 | 3 | 0.00001 | 0.319042 |
| 448 | IQCB1 | Insulin-like 3 | 3 | 0 | 0.289687 |
| 449 | KCNJ10 | ATP-sensitive inward rectifier potassium channel 1 | 3 | 0.66667 | 1 |
| 450 | KLRK1 | Kelch-like protein 3 | 3 | 0.00697 | 0.24677 |
| 451 | LHX4 | LIM/homeobox protein Lhx3 | 3 | 0.00697 | 0.238254 |
| 452 | LMOD1 | Hepatic triacylglycerol lipase | 3 | 0 | 0.253428 |
| 453 | MTR | Methylenetetrahydrofolate reductase | 3 | 0.00697 | 0.192735 |
| 454 | NEB | Neural cell adhesion molecule 1 | 3 | 0 | 0.255918 |
| 455 | NLRP1 | Homeobox protein Nkx-2.5 | 3 | 0.00003 | 0.27829 |
| 456 | NPPA | Neurogenic locus notch homolog protein 4 | 3 | 0.00358 | 0.276678 |
| 457 | PCNT | Paired box protein Pax-8 | 3 | 0 | 0.289687 |
| 458 | PRICKLE1 | Peroxiredoxin-1 | 3 | 0 | 0.270156 |
| 459 | PRKG1 | Serine/threonine-protein kinase D1 | 3 | 0.00697 | 0.297817 |
| 460 | RNF213 | Ribonuclease T2 | 3 | 0 | 0.263812 |
| 461 | SELL | E-selectin | 3 | 0.00006 | 0.288374 |
| 462 | SKI | NAD-dependent protein deacetylase sirtuin-1 | 3 | 0 | 0.282823 |
| 463 | SOST | Extracellular superoxide dismutase [Cu-Zn] | 3 | 0.00088 | 0.285359 |
| 464 | TG | Serotransferrin | 3 | 0.00074 | 0.268636 |
| 465 | TLR1 | Metalloproteinase inhibitor 2 | 3 | 0.00001 | 0.282127 |
| 466 | WNK4 | Serine/threonine-protein kinase WNK1 | 3 | 0.00439 | 0.209813 |
| 467 | XRCC1 | Xanthine dehydrogenase/oxidase [Includes: Xanthine dehydrogenase | 3 | 0.00004 | 0.235705 |
| 468 | ACTC1 | Actin, alpha cardiac muscle 1 | 2 | 0 | 0.203987 |
| 469 | ADA | Adenosine deaminase | 2 | 0.01389 | 0.226214 |
| 470 | ADAMTS13 | A disintegrin and metalloproteinase with thrombospondin motifs 13 | 2 | 0.00349 | 0.276145 |
| 471 | ALPP | Alkaline phosphatase, placental type | 2 | 0 | 1 |
| 472 | ATM | Serine-protein kinase ATM | 2 | 0 | 0.302215 |
| 473 | AUTS2 | Autism susceptibility gene 2 protein | 2 | 0 | 0.272598 |
| 474 | BSND | Barttin | 2 | 1 | 1 |
| 475 | C1QA | Complement C1q subcomponent subunit A | 2 | 0.00001 | 0.263691 |
| 476 | CCL17 | C-C motif chemokine 17 | 2 | 0 | 0.27208 |
| 477 | CYP21A2 | Steroid 21-hydroxylase | 2 | 0.00349 | 0.227381 |
| 478 | CYP2D6 | Cytochrome P450 2D6 | 2 | 0 | 0.230028 |
| 479 | FCGR2B | Low affinity immunoglobulin gamma Fc region receptor II-b | 2 | 0.00004 | 0.283523 |
| 480 | FLNB | Filamin-B | 2 | 0 | 0.30063 |
| 481 | GC | Vitamin D-binding protein | 2 | 0.00004 | 0.26676 |
| 482 | GCH1 | GTP cyclohydrolase 1 | 2 | 0 | 1 |
| 483 | GHRL | Somatotropin | 2 | 0 | 0.296124 |
| 484 | GPT | Progonadoliberin-1 | 2 | 0 | 0.297972 |
| 485 | HMGA2 | HLA class I histocompatibility antigen, alpha chain G | 2 | 0.00001 | 0.301897 |
| 486 | HPRT1 | Haptoglobin | 2 | 0 | 0.156131 |
| 487 | IDO1 | Inducible T-cell costimulator | 2 | 0.00048 | 0.227291 |
| 488 | IL17RA | Interleukin-17F | 2 | 0 | 0.265278 |
| 489 | IL21 | Interleukin-2 | 2 | 0 | 0.31922 |
| 490 | KCNB1 | Voltage-gated potassium channel subunit beta-2 | 2 | 0.00044 | 0.27482 |
| 491 | KCNJ1 | Potassium voltage-gated channel subfamily D member 3 | 2 | 0.00008 | 0.186402 |
| 492 | KCNJ16 | ATP-sensitive inward rectifier potassium channel 10 | 2 | 0 | 0.75 |
| 493 | KCNJ2 | Inward rectifier potassium channel 16 | 2 | 0 | 0.75 |
| 494 | KCNQ2 | Inward rectifier potassium channel 2 | 2 | 0.00027 | 0.283103 |
| 495 | KCNQ3 | Potassium voltage-gated channel subfamily KQT member 2 | 2 | 0.00027 | 0.283103 |
| 496 | KRT19 | NKG2-D type II integral membrane protein | 2 | 0.00349 | 0.297508 |
| 497 | LHX3 | Lutropin-choriogonadotropic hormone receptor | 2 | 0 | 0.238056 |
| 498 | MAPT | Mitogen-activated protein kinase 3 | 2 | 0 | 0.310065 |
| 499 | MECP2 | Adrenocorticotropic hormone receptor | 2 | 0 | 0.272209 |
| 500 | MME | Promotilin | 2 | 0.00083 | 0.285075 |
| 501 | MRAS | Myeloperoxidase | 2 | 0 | 0.278968 |
| 502 | MTHFR | Ras-related protein M-Ras | 2 | 0 | 0.161682 |
| 503 | MTRR | Methionine synthase | 2 | 0 | 0.161682 |
| 504 | OGG1 | Obscurin | 2 | 0 | 0.231609 |
| 505 | PLA2G7 | Phosphatidylinositol 3-kinase regulatory subunit alpha | 2 | 0 | 0.283945 |
| 506 | POU1F1 | Pro-opiomelanocortin | 2 | 0.00349 | 0.192605 |
| 507 | PRKD1 | Protein kinase C beta type | 2 | 0 | 0.286643 |
| 508 | PTPN3 | Tyrosine-protein phosphatase non-receptor type 22 | 2 | 0 | 0.311075 |
| 509 | PTS | Receptor-type tyrosine-protein phosphatase C | 2 | 0 | 1 |
| 510 | PVALB | Pentraxin-related protein PTX3 | 2 | 0.00049 | 0.258224 |
| 511 | REN | RAS guanyl-releasing protein 1 | 2 | 0.00001 | 0.305763 |
| 512 | S100A9 | Ryanodine receptor 2 | 2 | 0 | 0.273639 |
| 513 | SCN1A | Protein S100-B | 2 | 0 | 0.276812 |
| 514 | SCN4A | Sodium channel subunit beta-1 | 2 | 0 | 0.276812 |
| 515 | SCN5A | Sodium channel protein type 4 subunit alpha | 2 | 0 | 0.276812 |
| 516 | SDHA | Syndecan-1 | 2 | 0 | 0.75 |
| 517 | SDHB | Succinate dehydrogenase [ubiquinone] flavoprotein subunit, mitochondrial | 2 | 0 | 0.75 |
| 518 | SHOC2 | #N/A | 2 | 0 | 0.278968 |
| 519 | SLC12A1 | Ski oncogene | 2 | 0.00265 | 0.222179 |
| 520 | SOX9 | Sclerostin | 2 | 0.00002 | 0.286071 |
| 521 | THBD | TGF-beta receptor type-1 | 2 | 0 | 0.292496 |
| 522 | TLR9 | Toll-like receptor 8 | 2 | 0 | 0.303818 |
| 523 | TNFRSF11B | Tumor necrosis factor receptor superfamily member 11A | 2 | 0.00002 | 0.281434 |
| 524 | TNFRSF25 | Tumor necrosis factor receptor superfamily member 1B | 2 | 0 | 0.277213 |
| 525 | TNFRSF4 | Tumor necrosis factor receptor superfamily member 25 | 2 | 0.00004 | 0.265032 |
| 526 | TNFSF4 | Tumor necrosis factor ligand superfamily member 13B | 2 | 0.0002 | 0.277886 |
| 527 | TYR | Non-receptor tyrosine-protein kinase TYK2 | 2 | 0 | 0.216471 |
| 528 | XDH | Protein Wnt-5a | 2 | 0 | 0.156131 |
| 529 | ABCB1 | ATP-dependent translocase ABCB1 | 1 | 0 | 0.294299 |
| 530 | ACP5 | Tartrate-resistant acid phosphatase type 5 | 1 | 0 | 0.275084 |
| 531 | ADAMTSL1 | ADAMTS-like protein 1 | 1 | 0 | 0.216471 |
| 532 | ALDH1A2 | Retinal dehydrogenase 2 | 1 | 0 | 0.22453 |
| 533 | AQP4 | Aquaporin-4 | 1 | 0 | 0.6 |
| 534 | BTNL2 | Butyrophilin-like protein 2 | 1 | 0 | 0.259159 |
| 535 | CA2 | Carbonic anhydrase 2 | 1 | 0 | 0.280059 |
| 536 | CASQ2 | Calsequestrin-2 | 1 | 0 | 1 |
| 537 | CD163 | Scavenger receptor cysteine-rich type 1 protein M130 | 1 | 0 | 0.262243 |
| 538 | CD46 | Membrane cofactor protein | 1 | 0 | 0.257297 |
| 539 | CD5 | T-cell surface glycoprotein CD5 | 1 | 0 | 0.246452 |
| 540 | CEACAM5 | Carcinoembryonic antigen-related cell adhesion molecule 5 | 1 | 0 | 0.240352 |
| 541 | CEL | Bile salt-activated lipase | 1 | 0 | 0.250656 |
| 542 | CHCHD10 | Coiled-coil-helix-coiled-coil-helix domain-containing protein 10, mitochondrial | 1 | 0 | 0.6 |
| 543 | CHGA | Chromogranin-A | 1 | 0 | 1 |
| 544 | CLCNKA | Chloride channel protein ClC-Ka | 1 | 0 | 0.666667 |
| 545 | CLCNKB | Chloride channel protein ClC-Kb | 1 | 0 | 0.666667 |
| 546 | DEFB4A | Beta-defensin 4A | 1 | 0 | 0.284933 |
| 547 | DFFA | DNA fragmentation factor subunit alpha | 1 | 0 | 0.215171 |
| 548 | DIO2 | Type II iodothyronine deiodinase | 1 | 0 | 1 |
| 549 | DIO3 | Thyroxine 5-deiodinase | 1 | 0 | 1 |
| 550 | DYNC2H1 | Cytoplasmic dynein 2 heavy chain 1 | 1 | 0 | 0.241874 |
| 551 | FOXD3 | Forkhead box protein D3 | 1 | 0 | 0.252423 |
| 552 | GSTM1 | Glutathione reductase, mitochondrial | 1 | 0 | 0.22453 |
| 553 | GSTP1 | Glutathione S-transferase Mu 1 | 1 | 0 | 0.22453 |
| 554 | HEXA | Histone deacetylase 2 | 1 | 0 | 0.243726 |
| 555 | HSD11B2 | Corticosteroid 11-beta-dehydrogenase isozyme 1 | 1 | 0 | 0.185317 |
| 556 | ICOS | Intercellular adhesion molecule 1 | 1 | 0 | 0.275613 |
| 557 | KCNA1 | Kalirin | 1 | 0 | 0.235512 |
| 558 | KCNA2 | Potassium voltage-gated channel subfamily A member 1 | 1 | 0 | 0.235512 |
| 559 | KCNA4 | Potassium voltage-gated channel subfamily A member 2 | 1 | 0 | 0.235512 |
| 560 | KCNC1 | Potassium voltage-gated channel subfamily B member 1 | 1 | 0 | 0.235512 |
| 561 | KCNC3 | Potassium voltage-gated channel subfamily C member 1 | 1 | 0 | 0.235512 |
| 562 | KCND3 | Potassium voltage-gated channel subfamily C member 3 | 1 | 0 | 0.235512 |
| 563 | KCNV2 | Potassium voltage-gated channel subfamily KQT member 3 | 1 | 0 | 0.235512 |
| 564 | KRT5 | Keratin, type I cytoskeletal 19 | 1 | 0 | 0.229384 |
| 565 | LGALS3 | Leptin | 1 | 0 | 0.263934 |
| 566 | MAGI2 | Lysozyme C | 1 | 0 | 0.280059 |
| 567 | MBL2 | Microtubule-associated protein tau | 1 | 0 | 0.257297 |
| 568 | MICA | Hepatocyte growth factor receptor | 1 | 0 | 0.197996 |
| 569 | MICB | MHC class I polypeptide-related sequence A | 1 | 0 | 0.197996 |
| 570 | MIF | MHC class I polypeptide-related sequence B | 1 | 0 | 0.267257 |
| 571 | MKI67 | Macrophage migration inhibitory factor | 1 | 0 | 1 |
| 572 | NAMPT | Myosin light chain kinase, smooth muscle | 1 | 0 | 0.156088 |
| 573 | NKX2-1 | Beta-nerve growth factor | 1 | 0 | 0.24913 |
| 574 | NPPB | Natriuretic peptides A | 1 | 0 | 0.216799 |
| 575 | NR1H2 | Natriuretic peptides B | 1 | 0 | 0.281434 |
| 576 | PAX8 | Poly [ADP-ribose] polymerase 1 | 1 | 0 | 0.271178 |
| 577 | PROP1 | Prolactin | 1 | 0 | 0.161545 |
| 578 | RASGRP1 | Retinoic acid receptor beta | 1 | 0 | 0.239849 |
| 579 | RYR2 | Retinoic acid receptor RXR-alpha | 1 | 0 | 1 |
| 580 | SLC12A3 | Solute carrier family 12 member 1 | 1 | 0 | 0.173479 |
| 581 | SLC2A1 | Solute carrier family 12 member 3 | 1 | 0 | 0.294299 |
| 582 | SPINT2 | Transcription factor SOX-9 | 1 | 0 | 0.287506 |
| 583 | SYP | STE20/SPS1-related proline-alanine-rich protein kinase | 1 | 0 | 1 |
| 584 | TBX19 | Antigen peptide transporter 2 | 1 | 0 | 0.271564 |
| 585 | TLR5 | Toll-like receptor 4 | 1 | 0 | 0.27105 |
| 586 | TMPRSS6 | Toll-like receptor 9 | 1 | 0 | 0.279649 |
| 587 | TOP2A | Troponin T, cardiac muscle | 1 | 0 | 1 |
| 588 | WNK1 | Wolframin | 1 | 0 | 0.2092 |
| 589 | ZNF423 | Transcriptional coactivator YAP1 | 1 | 0 | 0.271178 |
