# Supplementary material for: Diosgenin From Dioscorea Nipponica Rhizoma Against Graves’ Disease—On Network Pharmacology and Experimental Evaluation
Source: Front Pharmacol. 2022 Jan 24;12:806829. doi: 10.3389/fphar.2021.806829 (PMC8819592; doi:10.3389/fphar.2021.806829)
Supplement: Supplementary file 4 [file Table3.docx]

**Table S1** Information of active componds of DNR after PPI analysis.

| Number | Name | Degree | Betweenness Centrality | Closeness Centrality |
| --- | --- | --- | --- | --- |
| 1 | HBIN024164 | 238 | 0.31259661 | 0.550129 |
| 2 | HBIN024134 | 198 | 0.01931533 | 0.406844 |
| 3 | HBIN035691 | 102 | 0.02721181 | 0.409178 |
| 4 | HBIN013184 | 101 | 0.02209629 | 0.408397 |
| 5 | HBIN029305 | 101 | 0.02496109 | 0.408397 |
| 6 | HBIN034743 | 100 | 0.02513884 | 0.407619 |
| 7 | HBIN015193 | 99 | 0.07775525 | 0.406844 |
| 8 | HBIN022206 | 99 | 0.03901321 | 0.406844 |
| 9 | HBIN028360 | 99 | 0.02142479 | 0.406844 |
| 10 | HBIN035672 | 99 | 0.09928495 | 0.406844 |
| 11 | HBIN047103 | 99 | 0.01476876 | 0.406844 |
| 12 | HBIN024173 | 98 | 0.01454534 | 0.406072 |
| 13 | HBIN032343 | 98 | 0.05613393 | 0.406072 |
| 14 | HBIN048520 | 98 | 0.06904429 | 0.406072 |
| 15 | HBIN019988 | 97 | 0.04006481 | 0.405303 |
| 16 | HBIN025419 | 97 | 0.01178626 | 0.405303 |
| 17 | HBIN031651 | 97 | 0.01928796 | 0.405303 |
| 18 | HBIN040122 | 97 | 0.07458387 | 0.405303 |
| 19 | HBIN004598 | 96 | 0.01038842 | 0.404537 |
| 20 | HBIN006771 | 96 | 0.02984349 | 0.403774 |
| 21 | HBIN023174 | 96 | 0.01038842 | 0.404537 |
| 22 | HBIN024178 | 96 | 0.01038842 | 0.404537 |
| 23 | HBIN037405 | 96 | 0.06002066 | 0.404537 |
| 24 | HBIN040296 | 96 | 0.02836071 | 0.404537 |
| 25 | HBIN024177 | 95 | 0.01065533 | 0.403774 |
| 26 | HBIN024181 | 95 | 0.01019799 | 0.403774 |
| 27 | HBIN025423 | 95 | 0.02409881 | 0.403774 |
| 28 | HBIN031652 | 95 | 0.01161513 | 0.403774 |
| 29 | HBIN048193 | 95 | 0.01078275 | 0.403774 |
| 30 | HBIN006839 | 94 | 0.01658983 | 0.403013 |
| 31 | HBIN031650 | 94 | 0.01443469 | 0.403013 |
| 32 | HBIN044218 | 94 | 0.01009708 | 0.403013 |
| 33 | HBIN023402 | 65 | 0.01748363 | 0.381462 |
| 34 | HBIN017786 | 44 | 0.02172256 | 0.36833 |
| 35 | HBIN018163 | 27 | 0.01357026 | 0.35786 |
