# Supplementary material for: Diosgenin From Dioscorea Nipponica Rhizoma Against Graves’ Disease—On Network Pharmacology and Experimental Evaluation
Source: Front Pharmacol. 2022 Jan 24;12:806829. doi: 10.3389/fphar.2021.806829 (PMC8819592; doi:10.3389/fphar.2021.806829)
Supplement: Supplementary file 5 [file Table4.docx]

**Table S2** Graves disease related target.

| Number | Gene name | Uniprot ID | Protein name | Database |
| --- | --- | --- | --- | --- |
| 1 | MAPK1 | P28482 | Mitogen-activated protein kinase 1 | GeneCards、CTD |
| 2 | MAPK3 | P27361 | Mitogen-activated protein kinase 3 | GeneCards、CTD |
| 3 | AKT1 | P31749 | RAC-alpha serine/threonine-protein kinase | GeneCards、CTD、DisGeNET |
| 4 | ITGB1 | P05556 | Integrin beta-1 | GeneCards、DisGeNET |
| 5 | PPBP | P02775 | Platelet basic protein | GeneCards |
| 6 | RAC1 | P63000 | Ras-related C3 botulinum toxin substrate 1 | GeneCards |
| 7 | MAPK14 | Q16539 | Mitogen-activated protein kinase 14 | GeneCards |
| 8 | CXCL1 | P09341 | Growth-regulated alpha protein | GeneCards、CTD |
| 9 | CDC42 | P60953 | Cell division control protein 42 homolog | GeneCards |
| 10 | TIMP1 | P01033 | Metalloproteinase inhibitor 1 | GeneCards、DisGeNET |
| 11 | HSP90AA1 | P07900 | Heat shock protein HSP 90-alpha | GeneCards |
| 12 | PSMB8 | P28062 | Proteasome subunit beta type-8 | GeneCards、DisGeNET |
| 13 | TRAF6 | Q9Y4K3 | TNF receptor-associated factor 6 | GeneCards、DisGeNET |
| 14 | SPTAN1 | Q13813 | Spectrin alpha chain, non-erythrocytic 1 | GeneCards |
| 15 | CD44 | P16070 | CD44 antigen | GeneCards |
| 16 | HLA-DRA | P01903 | HLA class II histocompatibility antigen, DR alpha chain | GeneCards |
| 17 | FOS | P01100 | Proto-oncogene c-Fos | GeneCards、CTD |
| 18 | TYK2 | P29597 | Non-receptor tyrosine-protein kinase TYK2 | GeneCards、CTD |
| 19 | RXRA | P19793 | Retinoic acid receptor RXR-alpha | GeneCards、CTD |
| 20 | HLA-DRB5 | Q30154 | HLA class II histocompatibility antigen, DR beta 5 chain | GeneCards |
| 21 | HLA-DQA2 | P01906 | HLA class II histocompatibility antigen, DQ alpha 2 chain | GeneCards、DisGeNET |
| 22 | CCR3 | P51677 | C-C chemokine receptor type 3 | |
| 23 | SHFM1 | #N/A | SH2B adapter protein 3 | GeneCards |
| 24 | LYZ | P61626 | Lysozyme C | GeneCards |
| 25 | IGFBP3 | P17936 | Insulin-like growth factor-binding protein 3 | GeneCards、CTD |
| 26 | CCL20 | P78556 | C-C motif chemokine 20 | GeneCards、CTD、DisGeNET |
| 27 | SOCS3 | O14543 | Suppressor of cytokine signaling 3 | GeneCards、DisGeNET |
| 28 | NCAM1 | P13591 | Neural cell adhesion molecule 1 | GeneCards、DisGeNET |
| 29 | HDAC1 | Q13547 | Histone deacetylase 1 | GeneCards、CTD |
| 30 | CSF1 | P09603 | Macrophage colony-stimulating factor 1 | GeneCards |
| 31 | CCR1 | P32246 | C-C chemokine receptor type 1 | GeneCards |
| 32 | GNAQ | P50148 | Guanine nucleotide-binding protein G | GeneCards |
| 33 | CLTC | Q00610 | Clathrin heavy chain 1 | GeneCards |
| 34 | BIRC3 | Q13489 | Baculoviral IAP repeat-containing protein 3 | GeneCards |
| 35 | SDC1 | P18827 | Syndecan-1 | GeneCards |
| 36 | F13A1 | P00488 | Coagulation factor XIII A chain | GeneCards |
| 37 | RUNX1 | Q01196 | Runt-related transcription factor 1 | GeneCards |
| 38 | IGFBP1 | P08833 | Insulin-like growth factor-binding protein 1 | GeneCards |
| 39 | CCR4 | P51679 | C-C chemokine receptor type 4 | GeneCards |
| 40 | CXCL11 | O14625 | C-X-C motif chemokine 11 | GeneCards、DisGeNET |
| 41 | CIITA | P33076 | MHC class II transactivator | GeneCards |
| 42 | IRF8 | Q02556 | Interferon regulatory factor 8 | GeneCards |
| 43 | BPI | P17213 | Bactericidal permeability-increasing protein | GeneCards |
| 44 | VEGFC | P49767 | Vascular endothelial growth factor C | GeneCards |
| 45 | CXCL9 | Q07325 | C-X-C motif chemokine 9 | GeneCards、DisGeNET |
| 46 | CXCR2 | P25025 | C-X-C chemokine receptor type 2 | GeneCards |
| 47 | CXCR5 | P32302 | C-X-C chemokine receptor type 5 | GeneCards、DisGeNET |
| 48 | GAL | P22466 | Galanin peptides [Cleaved into: Galanin; Galanin message-associated peptide | GeneCards |
| 49 | HSPA8 | P11142 | Heat shock cognate 71 kDa protein | GeneCards |
| 50 | CSF2 | P04141 | Granulocyte-macrophage colony-stimulating factor | GeneCards |
| 51 | MAP3K7 | O43318 | Mitogen-activated protein kinase kinase kinase 7 | GeneCards |
| 52 | TRIM31 | Q9BZY9 | E3 ubiquitin-protein ligase TRIM31 | GeneCards |
| 53 | HIF1A | Q16665 | Hypoxia-inducible factor 1-alpha | GeneCards、CTD、DisGeNET |
| 54 | TAB2 | Q9NYJ8 | TGF-beta-activated kinase 1 and MAP3K7-binding protein 2 | GeneCards、DisGeNET |
| 55 | GNAO1 | P09471 | Guanine nucleotide-binding protein G | GeneCards、DisGeNET |
| 56 | IRAK1 | P51617 | Interleukin-1 receptor-associated kinase 1 | GeneCards |
| 57 | MMP8 | P22894 | Neutrophil collagenase | GeneCards |
| 58 | LTF | P02788 | Lactotransferrin | GeneCards |
| 59 | SERPINF2 | P08697 | Alpha-2-antiplasmin | GeneCards |
| 60 | PPARA | Q07869 | Peroxisome proliferator-activated receptor alpha | GeneCards |
| 61 | EZR | P15311 | Ezrin | GeneCards |
| 62 | PLAUR | Q03405 | Urokinase plasminogen activator surface receptor | GeneCards |
| 63 | PTX3 | P26022 | Pentraxin-related protein PTX3 | GeneCards |
| 64 | CD86 | P42081 | T-lymphocyte activation antigen CD86 | GeneCards |
| 65 | GNA11 | P29992 | Guanine nucleotide-binding protein subunit alpha-11 | GeneCards |
| 66 | PTPN2 | P17706 | Tyrosine-protein phosphatase non-receptor type 2 | GeneCards |
| 67 | NFKB2 | Q00653 | Nuclear factor NF-kappa-B p100 subunit | GeneCards、DisGeNET |
| 68 | PTHLH | P12272 | Parathyroid hormone-related protein | GeneCards |
| 69 | FSHR | P23945 | Follicle-stimulating hormone receptor | GeneCards |
| 70 | INSL3 | P51460 | Insulin-like 3 | GeneCards、DisGeNET |
| 71 | HRH2 | P25021 | Histamine H2 receptor | GeneCards |
| 72 | ADRB1 | P08588 | Beta-1 adrenergic receptor | GeneCards、DisGeNET |
| 73 | MC2R | Q01718 | Adrenocorticotropic hormone receptor | GeneCards |
| 74 | LHCGR | P22888 | Lutropin-choriogonadotropic hormone receptor | GeneCards、DisGeNET |
| 75 | ADCYAP1 | P18509 | Pituitary adenylate cyclase-activating polypeptide | GeneCards |
| 76 | HDAC2 | Q92769 | Histone deacetylase 2 | GeneCards、DisGeNET |
| 77 | KALRN | O60229 | Kalirin | GeneCards |
| 78 | PRKCB | P05771 | Protein kinase C beta type | GeneCards |
| 79 | HSPA1A | P0DMV8 | Heat shock 70 kDa protein 1A | GeneCards、DisGeNET |
| 80 | ITGA4 | P13612 | Integrin alpha-4 | GeneCards |
| 81 | SLPI | P03973 | Antileukoproteinase | GeneCards |
| 82 | SIRT1 | Q96EB6 | NAD-dependent protein deacetylase sirtuin-1 | GeneCards、CTD |
| 83 | TNFSF12 | O43508 | Tumor necrosis factor ligand superfamily member 12 | GeneCards |
| 84 | HSPA5 | P11021 | Endoplasmic reticulum chaperone BiP | GeneCards、DisGeNET |
| 85 | AKT2 | P31751 | RAC-beta serine/threonine-protein kinase | GeneCards |
| 86 | CDKN1B | P46527 | Cyclin-dependent kinase inhibitor 1B | GeneCards、CTD |
| 87 | GAST | P01350 | Gastrin [Cleaved into: Gastrin-71 | GeneCards、CTD |
| 88 | CD59 | P13987 | CD59 glycoprotein | GeneCards |
| 89 | TNFSF13B | Q9Y275 | Tumor necrosis factor ligand superfamily member 13B | GeneCards、DisGeNET |
| 90 | PLCG2 | P16885 | 1-phosphatidylinositol 4,5-bisphosphate phosphodiesterase gamma-2 | GeneCards |
| 91 | WNT5A | P41221 | Protein Wnt-5a | GeneCards |
| 92 | TNFRSF13C | Q96RJ3 | Tumor necrosis factor receptor superfamily member 13C | GeneCards |
| 93 | BCL2L1 | Q07817 | Bcl-2-like protein 1 | |
| 94 | NOS3 | P29474 | Nitric oxide synthase, endothelial | GeneCards、DisGeNET |
| 95 | RARA | P10276 | Retinoic acid receptor alpha | GeneCards |
| 96 | IL23A | Q9NPF7 | Interleukin-23 subunit alpha | GeneCards、DisGeNET |
| 97 | HLA-DMA | P28067 | HLA class II histocompatibility antigen, DM alpha chain | GeneCards |
| 98 | IFNB1 | P01574 | Interferon beta | GeneCards |
| 99 | PGR | P06401 | Progesterone receptor | GeneCards |
| 100 | IL6R | P08887 | Interleukin-6 receptor subunit alpha | GeneCards、CTD |
| 101 | KIT | P10721 | Mast/stem cell growth factor receptor Kit | GeneCards、CTD |
| 102 | SMARCB1 | Q12824 | SWI/SNF-related matrix-associated actin-dependent regulator of chromatin subfamily B member 1 | GeneCards |
| 103 | CSNK2B | P67870 | Casein kinase II subunit beta | GeneCards |
| 104 | EPAS1 | Q99814 | Endothelial PAS domain-containing protein 1 | GeneCards |
| 105 | ITGA6 | P23229 | Integrin alpha-6 | GeneCards |
| 106 | RARB | P10826 | Retinoic acid receptor beta | GeneCards |
| 107 | ITGB4 | P16144 | Integrin beta-4 | GeneCards |
| 108 | MX1 | P20591 | Interferon-induced GTP-binding protein Mx1 | GeneCards、CTD |
| 109 | ETS1 | P14921 | Protein C-ets-1 | GeneCards、DisGeNET |
| 110 | IL6ST | P40189 | Interleukin-6 receptor subunit beta | GeneCards |
| 111 | L1CAM | P32004 | Neural cell adhesion molecule L1 | GeneCards |
| 112 | CYB5R3 | P00387 | NADH-cytochrome b5 reductase 3 | GeneCards |
| 113 | BLK | P51451 | Tyrosine-protein kinase Blk | GeneCards、DisGeNET |
| 114 | TAP1 | Q03518 | Antigen peptide transporter 1 | GeneCards、DisGeNET |
| 115 | TXN | P10599 | Thioredoxin | GeneCards |
| 116 | TEK | Q02763 | Angiopoietin-1 receptor | GeneCards、CTD |
| 117 | YAP1 | P46937 | Transcriptional coactivator YAP1 | GeneCards |
| 118 | PIK3CG | P48736 | Phosphatidylinositol 4,5-bisphosphate 3-kinase catalytic subunit gamma isoform | GeneCards、DisGeNET |
| 119 | FGFR4 | P22455 | Fibroblast growth factor receptor 4 | GeneCards |
| 120 | MMP7 | P09237 | Matrilysin | GeneCards |
| 121 | RAC2 | P15153 | Ras-related C3 botulinum toxin substrate 2 | GeneCards、DisGeNET |
| 122 | S100B | P04271 | Protein S100-B | GeneCards、DisGeNET |
| 123 | NOTCH4 | Q99466 | Neurogenic locus notch homolog protein 4 | GeneCards、DisGeNET |
| 124 | IL15 | P40933 | Interleukin-15 | GeneCards、DisGeNET |
| 125 | HSPA1L | P34931 | Heat shock 70 kDa protein 1-like | GeneCards |
| 126 | TGFA | P01135 | Protransforming growth factor alpha [Cleaved into: Transforming growth factor alpha | GeneCards |
| 127 | AGER | Q15109 | Advanced glycosylation end product-specific receptor | GeneCards |
| 128 | BMPR1A | P36894 | Bone morphogenetic protein receptor type-1A | GeneCards |
| 129 | IL18R1 | Q13478 | Interleukin-18 receptor 1 | GeneCards、DisGeNET |
| 130 | SLIT2 | O94813 | Slit homolog 2 protein | GeneCards、DisGeNET |
| 131 | TNFSF10 | P50591 | Tumor necrosis factor ligand superfamily member 10 | GeneCards |
| 132 | PECAM1 | P16284 | Platelet endothelial cell adhesion molecule | GeneCards |
| 133 | ANGPT1 | Q15389 | Angiopoietin-1 | GeneCards |
| 134 | IL37 | Q9NZH6 | Interleukin-37 | GeneCards、DisGeNET |
| 135 | HSPA4 | P34932 | Heat shock 70 kDa protein 4 | GeneCards、DisGeNET |
| 136 | PLAT | P00750 | Tissue-type plasminogen activator | GeneCards、CTD |
| 137 | POU5F1 | Q01860 | POU domain, class 5, transcription factor 1 | GeneCards |
| 138 | PDGFA | P04085 | Platelet-derived growth factor subunit A | GeneCards |
| 139 | TBX21 | Q9UL17 | T-box transcription factor TBX21 | GeneCards、DisGeNET |
| 140 | CYFIP2 | Q96F07 | Cytoplasmic FMR1-interacting protein 2 | GeneCards |
| 141 | TLR6 | Q9Y2C9 | Toll-like receptor 6 | GeneCards、DisGeNET |
| 142 | APOH | P02749 | Beta-2-glycoprotein 1 | GeneCards |
| 143 | DNMT3A | Q9Y6K1 | DNA | GeneCards |
| 144 | IL18RAP | O95256 | Interleukin-18 receptor accessory protein | GeneCards、DisGeNET |
| 145 | IL17F | Q96PD4 | Interleukin-17F | GeneCards、DisGeNET |
| 146 | SH2B3 | Q9UQQ2 | SH2B adapter protein 3 | GeneCards、DisGeNET |
| 147 | HSD11B1 | P28845 | Corticosteroid 11-beta-dehydrogenase isozyme 1 | GeneCards、CTD |
| 148 | GFRA1 | P56159 | GDNF family receptor alpha-1 | GeneCards |
| 149 | DCC | P43146 | Netrin receptor DCC | GeneCards、CTD |
| 150 | GPX1 | P07203 | Glutathione peroxidase 1 | GeneCards、CTD |
| 151 | USP8 | P40818 | Ubiquitin carboxyl-terminal hydrolase 8 | GeneCards |
| 152 | SOD3 | P08294 | Extracellular superoxide dismutase [Cu-Zn] | GeneCards |
| 153 | DNMT3B | Q9UBC3 | DNA | GeneCards |
| 154 | SCN1B | Q07699 | Sodium channel subunit beta-1 | GeneCards |
| 155 | TLR7 | Q9NYK1 | Toll-like receptor 7 | GeneCards、DisGeNET |
| 156 | TLR8 | Q9NR97 | Toll-like receptor 8 | GeneCards、DisGeNET |
| 157 | IL9 | P15248 | Interleukin-9 | GeneCards |
| 158 | IFNA2 | P01563 | Interferon alpha-2 | GeneCards、DisGeNET |
| 159 | IL33 | O95760 | Interleukin-33 | GeneCards |
| 160 | RORC | P51449 | Nuclear receptor ROR-gamma | GeneCards |
| 161 | PGF | P49763 | Placenta growth factor | GeneCards |
| 162 | CEP57 | Q86XR8 | Centrosomal protein of 57 kDa | GeneCards |
| 163 | OBSCN | Q5VST9 | Obscurin | GeneCards |
| 164 | STK39 | Q9UEW8 | STE20/SPS1-related proline-alanine-rich protein kinase | GeneCards |
| 165 | CD69 | Q07108 | Early activation antigen CD69 | GeneCards |
| 166 | PRDX1 | Q06830 | Peroxiredoxin-1 | GeneCards、CTD |
| 167 | CACNA1D | Q01668 | Voltage-dependent L-type calcium channel subunit alpha-1D | GeneCards |
| 168 | BAK1 | Q16611 | Bcl-2 homologous antagonist/killer | GeneCards、DisGeNET |
| 169 | ATP1B2 | P14415 | Sodium/potassium-transporting ATPase subunit beta-2 | GeneCards |
| 170 | ATP1B4 | Q9UN42 | Protein ATP1B4 | GeneCards |
| 171 | ATF6 | P18850 | Cyclic AMP-dependent transcription factor ATF-6 alpha | GeneCards |
| 172 | ARHGEF2 | Q92974 | Rho guanine nucleotide exchange factor 2 | GeneCards |
| 173 | LIPC | P11150 | Hepatic triacylglycerol lipase | GeneCards |
| 174 | PARP1 | P09874 | Poly [ADP-ribose] polymerase 1 | GeneCards、DisGeNET |
| 175 | ANGPT2 | O15123 | Angiopoietin-2 | GeneCards |
| 176 | AHCY | P23526 | Adenosylhomocysteinase | GeneCards |
| 177 | DLL4 | Q9NR61 | Delta-like protein 4 | GeneCards、DisGeNET |
| 178 | PNP | P00491 | Purine nucleoside phosphorylase | GeneCards |
| 179 | PRICKLE1 | Q96MT3 | Prickle-like protein 1 | GeneCards、DisGeNET |
| 180 | LHX4 | Q969G2 | LIM/homeobox protein Lhx4 | GeneCards |
| 181 | WNK4 | Q96J92 | Serine/threonine-protein kinase WNK4 | GeneCards |
| 182 | IL22 | Q9GZX6 | Interleukin-22 | GeneCards、DisGeNET |
| 183 | KLRK1 | P26718 | NKG2-D type II integral membrane protein | GeneCards |
| 184 | IL11 | P20809 | Interleukin-11 | GeneCards |
| 185 | TLR1 | Q15399 | Toll-like receptor 1 | GeneCards、DisGeNET |
| 186 | SKI | P12755 | Ski oncogene | GeneCards |
| 187 | GLS | O94925 | Glutaminase kidney isoform, mitochondrial | GeneCards |
| 188 | FLT3 | P36888 | Receptor-type tyrosine-protein kinase FLT3 | GeneCards、CTD |
| 189 | DDC | P20711 | Aromatic-L-amino-acid decarboxylase | GeneCards |
| 190 | CS | O75390 | Citrate synthase, mitochondrial | GeneCards |
| 191 | GZMB | P10144 | Granzyme B | GeneCards |
| 192 | DPP4 | P27487 | Dipeptidyl peptidase 4 | GeneCards |
| 193 | ACVR1 | Q04771 | Activin receptor type-1 | GeneCards |
| 194 | POU1F1 | P28069 | Pituitary-specific positive transcription factor 1 | GeneCards |
| 195 | LHX3 | Q9UBR4 | LIM/homeobox protein Lhx3 | GeneCards |
| 196 | KRT19 | P08727 | Keratin, type I cytoskeletal 19 | GeneCards |
| 197 | KCNJ16 | Q9NPI9 | Inward rectifier potassium channel 16 | GeneCards |
| 198 | S100A9 | P06702 | Protein S100-A9 | GeneCards、CTD、DisGeNET |
| 199 | IL17RA | Q96F46 | Interleukin-17 receptor A | GeneCards、DisGeNET |
| 200 | TLR9 | Q9NR96 | Toll-like receptor 9 | GeneCards、DisGeNET |
| 201 | TNFSF4 | P23510 | Tumor necrosis factor ligand superfamily member 4 | GeneCards |
| 202 | PRKD1 | Q15139 | Serine/threonine-protein kinase D1 | GeneCards |
| 203 | HPRT1 | P00492 | Hypoxanthine-guanine phosphoribosyltransferase | GeneCards |
| 204 | HMGA2 | P52926 | High mobility group protein HMGI-C | GeneCards |
| 205 | PTPN3 | P26045 | Tyrosine-protein phosphatase non-receptor type 3 | GeneCards |
| 206 | IDO1 | P14902 | Indoleamine 2,3-dioxygenase 1 | GeneCards |
| 207 | TNFRSF4 | P43489 | Tumor necrosis factor receptor superfamily member 4 | GeneCards |
| 208 | FCGR2B | P31994 | Low affinity immunoglobulin gamma Fc region receptor II-b | GeneCards、DisGeNET |
| 209 | CCL17 | Q92583 | C-C motif chemokine 17 | GeneCards |
| 210 | TNFRSF25 | Q93038 | Tumor necrosis factor receptor superfamily member 25 | GeneCards、DisGeNET |
| 211 | C1QA | P02745 | Complement C1q subcomponent subunit A | GeneCards |
| 212 | AUTS2 | Q8WXX7 | Autism susceptibility gene 2 protein | GeneCards |
| 213 | OGG1 | O15527 | N-glycosylase/DNA lyase [Includes: 8-oxoguanine DNA glycosylase | GeneCards、DisGeNET |
| 214 | PTS | Q03393 | 6-pyruvoyl tetrahydrobiopterin synthase | GeneCards |
| 215 | ZNF423 | Q2M1K9 | Zinc finger protein 423 | GeneCards |
| 216 | PROP1 | O75360 | Homeobox protein prophet of Pit-1 | GeneCards |
| 217 | NR1H2 | P55055 | Oxysterols receptor LXR-beta | GeneCards |
| 218 | NAMPT | P43490 | Nicotinamide phosphoribosyltransferase | GeneCards、DisGeNET |
| 219 | TOP2A | P11388 | DNA topoisomerase 2-alpha | GeneCards |
| 220 | MKI67 | P46013 | Proliferation marker protein Ki-67 | GeneCards、CTD |
| 221 | LGALS3 | P17931 | Galectin-3 | GeneCards、CTD |
| 222 | TMPRSS6 | Q8IU80 | Transmembrane protease serine 6 | GeneCards |
| 223 | MICB | Q29980 | MHC class I polypeptide-related sequence B | GeneCards |
| 224 | KCNC1 | P48547 | Potassium voltage-gated channel subfamily C member 1 | GeneCards |
| 225 | KCNA4 | P22459 | Potassium voltage-gated channel subfamily A member 4 | GeneCards、CTD |
| 226 | RASGRP1 | O95267 | RAS guanyl-releasing protein 1 | GeneCards |
| 227 | SPINT2 | O43291 | Kunitz-type protease inhibitor 2 | GeneCards |
| 228 | FOXD3 | Q9UJU5 | Forkhead box protein D3 | GeneCards、DisGeNET |
| 229 | DIO3 | P55073 | Thyroxine 5-deiodinase | GeneCards、CTD |
| 230 | DFFA | O00273 | DNA fragmentation factor subunit alpha | GeneCards |
| 231 | DEFB4A | O15263 | Beta-defensin 4A | GeneCards |
| 232 | MAGI2 | Q86UL8 | Membrane-associated guanylate kinase, WW and PDZ domain-containing protein 2 | GeneCards、DisGeNET |
| 233 | CHGA | P10645 | Chromogranin-A | GeneCards |
| 234 | CEL | P19835 | Bile salt-activated lipase | GeneCards |
| 235 | CEACAM5 | P06731 | Carcinoembryonic antigen-related cell adhesion molecule 5 | GeneCards |
| 236 | CD5 | P06127 | T-cell surface glycoprotein CD5 | GeneCards |
| 237 | ICOS | Q9Y6W8 | Inducible T-cell costimulator | GeneCards、DisGeNET |
| 238 | CD163 | Q86VB7 | Scavenger receptor cysteine-rich type 1 protein M130 | GeneCards |
| 239 | CASQ2 | O14958 | Calsequestrin-2 | GeneCards |
| 240 | CA2 | P00918 | Carbonic anhydrase 2 | GeneCards |
| 241 | CD46 | P15529 | Membrane cofactor protein | GeneCards |
| 242 | AQP4 | P55087 | Aquaporin-4 | GeneCards、DisGeNET |
| 243 | ALDH1A2 | O94788 | Retinal dehydrogenase 2 | GeneCards |
| 244 | ADAMTSL1 | Q8N6G6 | ADAMTS-like protein 1 | |
| 245 | ABCB1 | P08183 | ATP-dependent translocase ABCB1 | CTD/GeneCardss/GAD |
| 246 | ACP5 | P13686 | Tartrate-resistant acid phosphatase type 5 | GeneCardss |
| 247 | ACTA2 | P62736 | Actin, aortic smooth muscle | GeneCardss |
| 248 | ACTC1 | P68032 | Actin, alpha cardiac muscle 1 | GeneCardss |
| 249 | ADA | P00813 | Adenosine deaminase | GeneCardss |
| 250 | ADAM17 | P78536 | Disintegrin and metalloproteinase domain-containing protein 17 | GeneCardss |
| 251 | ADAMTS13 | Q76LX8 | A disintegrin and metalloproteinase with thrombospondin motifs 13 | GeneCardss |
| 252 | ADIPOQ | Q15848 | Adiponectin | GeneCardss |
| 253 | ADM | P35318 | ADM [Cleaved into: Adrenomedullin | CTD |
| 254 | ADRB2 | P07550 | Beta-2 adrenergic receptor | GeneCardss/GAD |
| 255 | AGT | P01019 | Angiotensinogen | GeneCardss |
| 256 | ALB | P02768 | Serum albumin | CTD/GeneCardss |
| 257 | ALPP | P05187 | Alkaline phosphatase, placental type | GeneCardss |
| 258 | APC | P25054 | Adenomatous polyposis coli protein | GeneCardss |
| 259 | APEX1 | P27695 | Apurinic-apyrimidinic endonuclease 1 | GAD |
| 260 | APOA1 | P02647 | Apolipoprotein A-I | GeneCardss |
| 261 | APOB | P04114 | Apolipoprotein B-100 | GeneCardss |
| 262 | ATM | Q13315 | Serine-protein kinase ATM | GeneCardss |
| 263 | ATP1A1 | P05023 | Sodium/potassium-transporting ATPase subunit alpha-1 | GeneCardss |
| 264 | ATP1A2 | P50993 | Sodium/potassium-transporting ATPase subunit alpha-2 | GeneCardss |
| 265 | ATP1B1 | P05026 | Sodium/potassium-transporting ATPase subunit beta-1 | GeneCardss |
| 266 | B2M | P61769 | Beta-2-microglobulin [Cleaved into: Beta-2-microglobulin form pI 5.3] | GeneCardss |
| 267 | BAX | Q07812 | Apoptosis regulator BAX | GeneCardss |
| 268 | BCL2 | P10415 | Apoptosis regulator Bcl-2 | GeneCardss |
| 269 | BGLAP | P02818 | Osteocalcin | GeneCardss |
| 270 | BMP4 | P12644 | Bone morphogenetic protein 4 | GeneCardss |
| 271 | BMP6 | P22004 | Bone morphogenetic protein 6 | GeneCardss |
| 272 | BMPR2 | Q13873 | Bone morphogenetic protein receptor type-2 | GeneCardss |
| 273 | BSND | Q8WZ55 | Barttin | GeneCardss |
| 274 | BTNL2 | Q9UIR0 | Butyrophilin-like protein 2 | GeneCardss |
| 275 | C4A | P0C0L4 | Complement C4-A | GeneCardss/GAD |
| 276 | CACNA1A | O00555 | Voltage-dependent P/Q-type calcium channel subunit alpha-1A | GeneCardss |
| 277 | CACNA1S | Q13698 | Voltage-dependent L-type calcium channel subunit alpha-1S | GeneCardss/OMIM |
| 278 | CACNB4 | O00305 | Voltage-dependent L-type calcium channel subunit beta-4 | GeneCardss |
| 279 | CALCA | P06881 | Calcitonin gene-related peptide 1 | GeneCardss |
| 280 | CALCR | P30988 | Calcitonin receptor | GeneCardss |
| 281 | CALR | P27797 | Calreticulin | GeneCardss |
| 282 | CASP8 | Q14790 | Caspase-8 | GeneCardss |
| 283 | CASR | P41180 | Extracellular calcium-sensing receptor | GeneCardss |
| 284 | CAT | P04040 | Catalase | GeneCardss |
| 285 | CCL2 | P13500 | C-C motif chemokine 2 | GeneCardss |
| 286 | CCL5 | P13501 | C-C motif chemokine 5 | GeneCardss |
| 287 | CCND1 | P24385 | G1/S-specific cyclin-D1 | GeneCardss |
| 288 | CCR5 | P51681 | C-C chemokine receptor type 5 | GeneCardss |
| 289 | CD274 | Q9NZQ7 | Programmed cell death 1 ligand 1 | GAD |
| 290 | CD28 | P10747 | T-cell-specific surface glycoprotein CD28 | GeneCardss |
| 291 | CD36 | P16671 | Platelet glycoprotein 4 | GeneCardss |
| 292 | CD40 | P25942 | Tumor necrosis factor receptor superfamily member 5 | TTD/GeneCards/GAD |
| 293 | CD40LG | P29965 | CD40 ligand | GeneCardss |
| 294 | CD55 | P08174 | Complement decay-accelerating factor | GeneCardss |
| 295 | CD79A | P11912 | B-cell antigen receptor complex-associated protein alpha chain | GeneCardss |
| 296 | CD80 | P33681 | T-lymphocyte activation antigen CD80 | GeneCardss |
| 297 | CDKN2A | Q8N726 | Tumor suppressor ARF | CTD/GeneCardss |
| 298 | CETP | P11597 | Cholesteryl ester transfer protein | GeneCardss |
| 299 | CGA | P01215 | Glycoprotein hormones alpha chain | GeneCardss |
| 300 | CHCHD10 | Q8WYQ3 | Coiled-coil-helix-coiled-coil-helix domain-containing protein 10, mitochondrial | GeneCardss |
| 301 | CLCNKA | P51800 | Chloride channel protein ClC-Ka | GeneCardss |
| 302 | CLCNKB | P51801 | Chloride channel protein ClC-Kb | GeneCardss |
| 303 | COG2 | Q14746 | Conserved oligomeric Golgi complex subunit 2 | GeneCardss |
| 304 | COL11A2 | P13942 | Collagen alpha-2 | GeneCardss |
| 305 | COL1A1 | P02452 | Collagen alpha-1 | GeneCardss |
| 306 | COL3A1 | P02461 | Collagen alpha-1 | GeneCardss |
| 307 | COL5A1 | P20908 | Collagen alpha-1 | GeneCardss |
| 308 | CP | P00450 | Ceruloplasmin | GeneCardss |
| 309 | CRLF1 | O75462 | Cytokine receptor-like factor 1 | GeneCardss |
| 310 | CRP | P02741 | C-reactive protein [Cleaved into: C-reactive protein | GeneCardss |
| 311 | CSF3 | P09919 | Granulocyte colony-stimulating factor | GeneCardss |
| 312 | CST3 | P01034 | Cystatin-C | GeneCardss |
| 313 | CTLA4 | P16410 | Cytotoxic T-lymphocyte protein 4 | DisGeNET/GeneCardss/OMIM/GAD |
| 314 | CTNNB1 | P35222 | Catenin beta-1 | GeneCardss |
| 315 | CTSD | P07339 | Cathepsin D | GeneCardss |
| 316 | CXCL10 | P02778 | C-X-C motif chemokine 10 | GeneCardss/GAD |
| 317 | CXCL12 | P48061 | Stromal cell-derived factor 1 | GeneCardss |
| 318 | CXCL8 | P10145 | Interleukin-8 | GeneCardss |
| 319 | CXCR3 | P49682 | C-X-C chemokine receptor type 3 | GeneCardss |
| 320 | CXCR4 | P61073 | C-X-C chemokine receptor type 4 | GeneCardss |
| 321 | CYP1A1 | P04798 | Cytochrome P450 1A1 | Drugbank/GAD |
| 322 | CYP21A2 | P08686 | Steroid 21-hydroxylase | GeneCardss |
| 323 | CYP27B1 | O15528 | 25-hydroxyvitamin D-1 alpha hydroxylase, mitochondrial | GeneCardss/GAD |
| 324 | CYP2D6 | P10635 | Cytochrome P450 2D6 | Drugbank/GeneCardss/GAD |
| 325 | CYP7A1 | P22680 | Cytochrome P450 7A1 | GeneCardss |
| 326 | DIO2 | Q92813 | Type II iodothyronine deiodinase | CTD/GeneCardss/OMIM/GAD |
| 327 | DNMT1 | P26358 | DNA | CTD/GeneCardss |
| 328 | DYNC2H1 | Q8NCM8 | Cytoplasmic dynein 2 heavy chain 1 | GeneCardss |
| 329 | EDN1 | P05305 | Endothelin-1 | GeneCardss |
| 330 | EDNRA | P25101 | Endothelin-1 receptor | GeneCardss |
| 331 | EGF | P01133 | Pro-epidermal growth factor | GeneCardss |
| 332 | EGFR | P00533 | Epidermal growth factor receptor | GeneCardss |
| 333 | ELANE | P08246 | Neutrophil elastase | GeneCardss |
| 334 | ENG | P17813 | Endoglin | GeneCardss |
| 335 | EPO | P01588 | Erythropoietin | GeneCardss |
| 336 | ERBB2 | P04626 | Receptor tyrosine-protein kinase erbB-2 | GeneCardss |
| 337 | ERBB3 | P21860 | Receptor tyrosine-protein kinase erbB-3 | GeneCardss |
| 338 | ERBB4 | Q15303 | Receptor tyrosine-protein kinase erbB-4 | GeneCardss |
| 339 | ESR1 | P03372 | Estrogen receptor | GeneCardss |
| 340 | ESR2 | Q92731 | Estrogen receptor beta | GeneCardss |
| 341 | F2 | P00734 | Prothrombin | GeneCardss |
| 342 | F3 | P13726 | Tissue factor | GeneCardss |
| 343 | F5 | P12259 | Coagulation factor V | GeneCardss |
| 344 | F9 | P00740 | Coagulation factor IX | GeneCardss |
| 345 | FAS | P25445 | Tumor necrosis factor receptor superfamily member 6 | GeneCardss/GAD |
| 346 | FASLG | P48023 | Tumor necrosis factor ligand superfamily member 6 | GeneCardss |
| 347 | FCGR2A | P12318 | Low affinity immunoglobulin gamma Fc region receptor II-a | GeneCardss |
| 348 | FCGR3B | O75015 | Low affinity immunoglobulin gamma Fc region receptor III-B | OMIM |
| 349 | FGA | P02671 | Fibrinogen alpha chain [Cleaved into: Fibrinopeptide A; Fibrinogen alpha chain] | GeneCardss |
| 350 | FGF1 | P05230 | Fibroblast growth factor 1 | CTD |
| 351 | FGF2 | P09038 | Fibroblast growth factor 2 | GeneCardss |
| 352 | FGF23 | Q9GZV9 | Fibroblast growth factor 23 | GeneCardss |
| 353 | FGFR3 | P22607 | Fibroblast growth factor receptor 3 | GeneCardss |
| 354 | FLNB | O75369 | Filamin-B | GeneCardss/OMIM |
| 355 | FLT1 | P17948 | Vascular endothelial growth factor receptor 1 | GeneCardss |
| 356 | FN1 | P02751 | Fibronectin | GeneCardss |
| 357 | FOXP3 | Q9BZS1 | Forkhead box protein P3 | GeneCardss/GAD |
| 358 | FXYD2 | P54710 | Sodium/potassium-transporting ATPase subunit gamma | GeneCardss |
| 359 | G6PD | P11413 | Glucose-6-phosphate 1-dehydrogenase | GeneCardss |
| 360 | GAD1 | Q99259 | Glutamate decarboxylase 1 | GeneCardss |
| 361 | GAD2 | Q05329 | Glutamate decarboxylase 2 | GeneCardss |
| 362 | GATA3 | P23771 | Trans-acting T-cell-specific transcription factor GATA-3 | GeneCardss |
| 363 | GC | P02774 | Vitamin D-binding protein | DisGeNET/OMIM/GAD |
| 364 | GCH1 | P30793 | GTP cyclohydrolase 1 | GeneCardss |
| 365 | GGT1 | P19440 | Glutathione hydrolase 1 proenzyme | GeneCardss |
| 366 | GH1 | P01241 | Somatotropin | GeneCardss |
| 367 | GHRL | Q9UBU3 | Appetite-regulating hormone | GeneCardss |
| 368 | GJA1 | P17302 | Gap junction alpha-1 protein | GeneCardss |
| 369 | GLI3 | P10071 | Transcriptional activator GLI3 | GeneCardss |
| 370 | GNAS | P84996 | Protein ALEX | GeneCardss |
| 371 | GNRH1 | P01148 | Progonadoliberin-1 | GeneCardss |
| 372 | GPT | P24298 | Alanine aminotransferase 1 | GeneCardss |
| 373 | GSR | P00390 | Glutathione reductase, mitochondrial | GeneCardss |
| 374 | GSTM1 | P09488 | Glutathione S-transferase Mu 1 | GAD |
| 375 | GSTP1 | P09211 | Glutathione S-transferase P | GAD |
| 376 | HEXA | P06865 | Beta-hexosaminidase subunit alpha | GeneCardss |
| 377 | HEXB | P07686 | Beta-hexosaminidase subunit beta | GeneCardss |
| 378 | HGF | P14210 | Hepatocyte growth factor | GeneCardss |
| 379 | HLA-A | P30450 | HLA class I histocompatibility antigen, A-26 alpha chain | GeneCardss/GAD |
| 380 | HLA-B | Q29836 | HLA class I histocompatibility antigen, B-67 alpha chain | GeneCardss/GAD |
| 381 | HLA-C | P30505 | HLA class I histocompatibility antigen, Cw-8 alpha chain | GeneCardss |
| 382 | HLA-DPA1 | P20036 | HLA class II histocompatibility antigen, DP alpha 1 chain | GeneCardss |
| 383 | HLA-DPB1 | P04440 | HLA class II histocompatibility antigen, DP beta 1 chain | GeneCardss/GAD |
| 384 | HLA-DQA1 | P01909 | HLA class II histocompatibility antigen, DQ alpha 1 chain | GeneCardss/GAD |
| 385 | HLA-DQB1 | P01920 | HLA class II histocompatibility antigen, DQ beta 1 chain | GeneCardss/GAD |
| 386 | HLA-DRB1 | Q5Y7A7 | HLA class II histocompatibility antigen, DRB1-13 beta chain | GeneCardss/GAD |
| 387 | HLA-G | P17693 | HLA class I histocompatibility antigen, alpha chain G | GeneCardss |
| 388 | HMGB1 | P09429 | High mobility group protein B1 | CTD |
| 389 | HP | P00738 | Haptoglobin | GeneCardss |
| 390 | HSD11B2 | P80365 | Corticosteroid 11-beta-dehydrogenase isozyme 2 | CTD |
| 391 | HSPG2 | P98160 | Basement membrane-specific heparan sulfate proteoglycan core protein | GeneCardss |
| 392 | ICAM1 | P05362 | Intercellular adhesion molecule 1 | GeneCardss/OMIM/GAD |
| 393 | IFIH1 | Q9BYX4 | Interferon-induced helicase C domain-containing protein 1 | DisGeNET/GeneCardss/OMIM/GAD |
| 394 | IFNG | P01579 | Interferon gamma | GeneCardss/GAD |
| 395 | IFNGR1 | P15260 | Interferon gamma receptor 1 | GeneCardss |
| 396 | IGF1 | P05019 | Insulin-like growth factor I | CTD/GeneCardss |
| 397 | IGF1R | P08069 | Insulin-like growth factor 1 receptor | GeneCardss |
| 398 | IGF2 | P01344 | Insulin-like growth factor II | GeneCardss |
| 399 | IL10 | P22301 | Interleukin-10 | GeneCardss/GAD |
| 400 | IL12A | P29459 | Interleukin-12 subunit alpha | GeneCardss |
| 401 | IL12B | P29460 | Interleukin-12 subunit beta | GeneCardss |
| 402 | IL13 | P35225 | Interleukin-13 | GeneCardss/OMIM/GAD |
| 403 | IL17A | Q16552 | Interleukin-17A | GeneCardss |
| 404 | IL18 | Q14116 | Interleukin-18 | GeneCardss |
| 405 | IL1A | P01583 | Interleukin-1 alpha | GeneCardss/GAD |
| 406 | IL1B | P01584 | Interleukin-1 beta | GeneCardss/GAD |
| 407 | IL1R1 | P14778 | Interleukin-1 receptor type 1 | GeneCardss |
| 408 | IL1RN | P18510 | Interleukin-1 receptor antagonist protein | GeneCardss/GAD |
| 409 | IL2 | P60568 | Interleukin-2 | GeneCardss/GAD |
| 410 | IL21 | Q9HBE4 | Interleukin-21 | GeneCardss |
| 411 | IL23R | Q5VWK5 | Interleukin-23 receptor | GeneCardss/GAD |
| 412 | IL2RA | P01589 | Interleukin-2 receptor subunit alpha | GeneCardss |
| 413 | IL2RB | P14784 | Interleukin-2 receptor subunit beta | GeneCardss |
| 414 | IL3 | P08700 | Interleukin-3 | GAD |
| 415 | IL4 | P05112 | Interleukin-4 | CTD/GeneCardss/OMIM/GAD |
| 416 | IL4R | P24394 | Interleukin-4 receptor subunit alpha | GAD |
| 417 | IL5 | P05113 | Interleukin-5 | CTD/GeneCardss |
| 418 | IL6 | P05231 | Interleukin-6 | GeneCardss/GAD |
| 419 | IL7 | P13232 | Interleukin-7 | GeneCardss |
| 420 | INS | P01308 | Insulin | GeneCardss/GAD |
| 421 | IQCB1 | Q15051 | IQ calmodulin-binding motif-containing protein 1 | GeneCardss |
| 422 | IRF1 | P10914 | Interferon regulatory factor 1 | GeneCardss/OMIM |
| 423 | IRF5 | Q13568 | Interferon regulatory factor 5 | GeneCardss |
| 424 | ITGAL | P20701 | Integrin alpha-L | GeneCardss |
| 425 | ITGAM | P11215 | Integrin alpha-M | GeneCardss |
| 426 | ITGB2 | P05107 | Integrin beta-2 | GeneCardss |
| 427 | ITGB3 | P05106 | Integrin beta-3 | GeneCardss |
| 428 | ITPR3 | Q14573 | Inositol 1,4,5-trisphosphate receptor type 3 | GAD |
| 429 | JAK2 | O60674 | Tyrosine-protein kinase JAK2 | GeneCardss |
| 430 | JUN | P05412 | Transcription factor AP-1 | CTD |
| 431 | KCNA1 | Q09470 | Potassium voltage-gated channel subfamily A member 1 | GeneCardss |
| 432 | KCNA2 | P16389 | Potassium voltage-gated channel subfamily A member 2 | GeneCardss |
| 433 | KCNAB2 | Q13303 | Voltage-gated potassium channel subunit beta-2 | GeneCardss |
| 434 | KCNB1 | Q14721 | Potassium voltage-gated channel subfamily B member 1 | GeneCardss |
| 435 | KCNC3 | Q14003 | Potassium voltage-gated channel subfamily C member 3 | GeneCardss |
| 436 | KCND3 | Q9UK17 | Potassium voltage-gated channel subfamily D member 3 | GeneCardss |
| 437 | KCNJ1 | P48048 | ATP-sensitive inward rectifier potassium channel 1 | GeneCardss |
| 438 | KCNJ10 | P78508 | ATP-sensitive inward rectifier potassium channel 10 | GeneCardss |
| 439 | KCNJ2 | P63252 | Inward rectifier potassium channel 2 | GeneCardss |
| 440 | KCNQ2 | O43526 | Potassium voltage-gated channel subfamily KQT member 2 | GeneCardss |
| 441 | KCNQ3 | O43525 | Potassium voltage-gated channel subfamily KQT member 3 | GeneCardss |
| 442 | KCNV2 | Q8TDN2 | Potassium voltage-gated channel subfamily V member 2 | GeneCardss |
| 443 | KDM6A | O15550 | Lysine-specific demethylase 6A | GAD |
| 444 | KIR3DL1 | P43629 | Killer cell immunoglobulin-like receptor 3DL1 | GAD |
| 445 | KITLG | P21583 | Kit ligand | GeneCardss |
| 446 | KLHL3 | Q9UH77 | Kelch-like protein 3 | GeneCardss |
| 447 | KRT5 | P13647 | Keratin, type II cytoskeletal 5 | GeneCardss |
| 448 | LAMB1 | P07942 | Laminin subunit beta-1 | CTD |
| 449 | LDLR | P01130 | Low-density lipoprotein receptor | GeneCardss |
| 450 | LEP | P41159 | Leptin | GeneCardss |
| 451 | LMOD1 | P29536 | Leiomodin-1 | GeneCardss/OMIM |
| 452 | LPA | P08519 | Apolipoprotein | GeneCardss |
| 453 | LPL | P06858 | Lipoprotein lipase | GeneCardss |
| 454 | LRP2 | P98164 | Low-density lipoprotein receptor-related protein 2 | GeneCardss/OMIM |
| 455 | LTA | P01374 | Lymphotoxin-alpha | GeneCardss/GAD |
| 456 | MAPT | P10636 | Microtubule-associated protein tau | GeneCardss |
| 457 | MBL2 | P11226 | Mannose-binding protein C | GeneCardss |
| 458 | MECP2 | P51608 | Methyl-CpG-binding protein 2 | GeneCardss |
| 459 | MET | P08581 | Hepatocyte growth factor receptor | GeneCardss |
| 460 | MICA | Q29983 | MHC class I polypeptide-related sequence A | GeneCardss/GAD |
| 461 | MIF | P14174 | Macrophage migration inhibitory factor | GeneCardss |
| 462 | MLN | P12872 | Promotilin [Cleaved into: Motilin; Motilin-associated peptide | GAD |
| 463 | MME | P08473 | Neprilysin | GeneCardss |
| 464 | MMP3 | P08254 | Stromelysin-1 | GeneCardss |
| 465 | MMP9 | P14780 | Matrix metalloproteinase-9 | CTD/GeneCardss |
| 466 | MPO | P05164 | Myeloperoxidase | Drugbank/GeneCardss |
| 467 | MRAS | O14807 | Ras-related protein M-Ras | GeneCardss |
| 468 | MTHFR | P42898 | Methylenetetrahydrofolate reductase | GeneCardss/GAD |
| 469 | MTR | Q99707 | Methionine synthase | GeneCardss |
| 470 | MTRR | Q9UBK8 | Methionine synthase reductase | GeneCardss |
| 471 | MUC1 | P15941 | Mucin-1 | GeneCardss |
| 472 | MYC | P01106 | Myc proto-oncogene protein | GeneCardss |
| 473 | MYH11 | P35749 | Myosin-11 | GeneCardss |
| 474 | MYLK | Q15746 | Myosin light chain kinase | GeneCardss |
| 475 | NEB | P20929 | Nebulin | GeneCardss |
| 476 | NEU1 | Q99519 | Sialidase-1 | GeneCardss |
| 477 | NF1 | P21359 | Neurofibromin | GeneCardss |
| 478 | NFKB1 | P19838 | Nuclear factor NF-kappa-B p105 subunit | GeneCardss/GAD |
| 479 | NFKBIA | P25963 | NF-kappa-B inhibitor alpha | GeneCardss |
| 480 | NGF | P01138 | Beta-nerve growth factor | GeneCardss |
| 481 | NKX2-1 | P43699 | Homeobox protein Nkx-2.1 | GeneCardss |
| 482 | NKX2-5 | P52952 | Homeobox protein Nkx-2.5 | GeneCardss |
| 483 | NLRP1 | Q9C000 | NACHT, LRR and PYD domains-containing protein 1 | GeneCardss |
| 484 | NOD2 | Q9HC29 | Nucleotide-binding oligomerization domain-containing protein 2 | GeneCardss |
| 485 | NOS2 | P35228 | Nitric oxide synthase, inducible | GeneCardss |
| 486 | NOTCH1 | P46531 | Neurogenic locus notch homolog protein 1 | GeneCardss |
| 487 | NOTCH2 | Q04721 | Neurogenic locus notch homolog protein 2 | GeneCardss |
| 488 | NPPA | P01160 | Natriuretic peptides A | GeneCardss |
| 489 | NPPB | P16860 | Natriuretic peptides B | GeneCardss |
| 490 | NR3C1 | P04150 | Nuclear receptor subfamily 3 group C member 1 | CTD/GeneCardss |
| 491 | PAX8 | Q06710 | Paired box protein Pax-8 | GeneCardss |
| 492 | PCNT | O95613 | Pericentrin | GeneCardss |
| 493 | PDCD1 | Q15116 | Programmed cell death protein 1 | GeneCardss |
| 494 | PDGFB | P01127 | Platelet-derived growth factor subunit B | GeneCardss |
| 495 | PDGFRA | P16234 | Platelet-derived growth factor receptor alpha | GeneCardss |
| 496 | PIK3R1 | P27986 | Phosphatidylinositol 3-kinase regulatory subunit alpha | GeneCardss |
| 497 | PLA2G7 | Q13093 | Platelet-activating factor acetylhydrolase | GeneCardss |
| 498 | PLG | P00747 | Plasminogen | GeneCardss |
| 499 | POMC | P01189 | Pro-opiomelanocortin | GeneCardss |
| 500 | PPARG | P37231 | Peroxisome proliferator-activated receptor gamma | GeneCardss/GAD |
| 501 | PRKAR1A | P10644 | cAMP-dependent protein kinase type I-alpha regulatory subunit | GeneCardss |
| 502 | PRKG1 | Q13976 | cGMP-dependent protein kinase 1 | GeneCardss |
| 503 | PRL | P01236 | Prolactin | GeneCardss |
| 504 | PRTN3 | P24158 | Myeloblastin | GeneCardss |
| 505 | PSAP | P07602 | Prosaposin | GeneCardss |
| 506 | PSEN2 | P49810 | Presenilin-2 | GeneCardss |
| 507 | PSMB9 | P28065 | Proteasome subunit beta type-9 | GeneCardss/GAD |
| 508 | PTEN | P60484 | Phosphatidylinositol 3,4,5-trisphosphate 3-phosphatase and dual-specificity protein phosphatase PTEN | GeneCardss |
| 509 | PTGS2 | P35354 | Prostaglandin G/H synthase 2 | CTD/GeneCardss |
| 510 | PTH | P01270 | Parathyroid hormone | GeneCardss |
| 511 | PTPN12 | Q05209 | Tyrosine-protein phosphatase non-receptor type 12 | GAD |
| 512 | PTPN22 | Q9Y2R2 | Tyrosine-protein phosphatase non-receptor type 22 | DisGeNET/GeneCardss/OMIM/GAD |
| 513 | PTPRC | P08575 | Receptor-type tyrosine-protein phosphatase C | GeneCardss/OMIM |
| 514 | PVALB | P20472 | Parvalbumin alpha | GeneCardss |
| 515 | RAF1 | P04049 | RAF proto-oncogene serine/threonine-protein kinase | GeneCardss |
| 516 | REN | P00797 | Renin | GeneCardss |
| 517 | RET | P07949 | Proto-oncogene tyrosine-protein kinase receptor Ret | GeneCardss |
| 518 | RETN | Q9HD89 | Resistin | GeneCardss |
| 519 | RHOH | Q15669 | Rho-related GTP-binding protein RhoH | GAD |
| 520 | RNASET2 | O00584 | Ribonuclease T2 | DisGeNET/GAD |
| 521 | RNF213 | Q63HN8 | E3 ubiquitin-protein ligase RNF213 | GeneCardss |
| 522 | RUNX2 | Q13950 | Runt-related transcription factor 2 | GeneCardss |
| 523 | RYR2 | Q92736 | Ryanodine receptor 2 | GeneCardss |
| 524 | SCN1A | P35498 | Sodium channel protein type 1 subunit alpha | GeneCardss |
| 525 | SCN4A | P35499 | Sodium channel protein type 4 subunit alpha | GeneCardss |
| 526 | SCN5A | Q14524 | Sodium channel protein type 5 subunit alpha | GeneCardss |
| 527 | SDHA | P31040 | Succinate dehydrogenase [ubiquinone] flavoprotein subunit, mitochondrial | GeneCardss |
| 528 | SDHB | P21912 | Succinate dehydrogenase [ubiquinone] iron-sulfur subunit, mitochondrial | GeneCardss |
| 529 | SELE | P16581 | E-selectin | GeneCardss/GAD |
| 530 | SELL | P14151 | L-selectin | GeneCardss |
| 531 | SELP | P16109 | P-selectin | GeneCardss |
| 532 | SERPINC1 | P01008 | Antithrombin-III | GeneCardss |
| 533 | SERPINE1 | P05121 | Plasminogen activator inhibitor 1 | GeneCardss |
| 534 | SHOC2 | Q9UQ13 | Leucine-rich repeat protein SHOC-2 | GeneCardss |
| 535 | SLC12A1 | Q13621 | Solute carrier family 12 member 1 | GeneCardss |
| 536 | SLC12A3 | P55017 | Solute carrier family 12 member 3 | GeneCardss |
| 537 | SLC2A1 | P11166 | Solute carrier family 2, facilitated glucose transporter member 1 | GeneCardss |
| 538 | SMAD4 | Q13485 | Mothers against decapentaplegic homolog 4 | GeneCardss |
| 539 | SNCA | P37840 | Alpha-synuclein | GeneCardss/OMIM |
| 540 | SOD1 | P00441 | Superoxide dismutase [Cu-Zn] | GeneCardss |
| 541 | SOD2 | P04179 | Superoxide dismutase | CTD/GeneCardss |
| 542 | SOST | Q9BQB4 | Sclerostin | GeneCardss |
| 543 | SOX9 | P48436 | Transcription factor SOX-9 | GeneCardss |
| 544 | SPP1 | P10451 | Osteopontin | GeneCardss |
| 545 | SRC | P12931 | Proto-oncogene tyrosine-protein kinase Src | GeneCardss |
| 546 | SST | P61278 | Somatostatin | GeneCardss |
| 547 | STAT3 | P40763 | Signal transducer and activator of transcription 3 | GeneCardss |
| 548 | STAT4 | Q14765 | Signal transducer and activator of transcription 4 | GeneCardss |
| 549 | STAT6 | P42226 | Signal transducer and activator of transcription 6 | GeneCardss |
| 550 | SYP | P08247 | Synaptophysin | GeneCardss |
| 551 | TAP2 | Q03519 | Antigen peptide transporter 2 | GeneCardss |
| 552 | TBX19 | O60806 | T-box transcription factor TBX19 | GeneCardss |
| 553 | TF | P02787 | Serotransferrin | GeneCardss |
| 554 | TG | P01266 | Thyroglobulin | CTD/GeneCardss/OMIM/GAD |
| 555 | TGFB1 | P01137 | Transforming growth factor beta-1 proprotein [Cleaved into: Latency-associated peptide | CTD/GeneCardss/GAD |
| 556 | TGFBR1 | P36897 | TGF-beta receptor type-1 | GeneCardss |
| 557 | THBD | P07204 | Thrombomodulin | GeneCardss |
| 558 | THRA | P10827 | Thyroid hormone receptor alpha | CTD |
| 559 | THRB | P10828 | Thyroid hormone receptor beta | CTD/GeneCardss |
| 560 | TIMP2 | P16035 | Metalloproteinase inhibitor 2 | GeneCardss |
| 561 | TLR3 | O15455 | Toll-like receptor 3 | GeneCardss |
| 562 | TLR4 | O00206 | Toll-like receptor 4 | GeneCardss |
| 563 | TLR5 | O60602 | Toll-like receptor 5 | GeneCardss |
| 564 | TNF | P01375 | Tumor necrosis factor | CTD/GeneCardss/GAD |
| 565 | TNFAIP3 | P21580 | Tumor necrosis factor alpha-induced protein 3 | GeneCardss |
| 566 | TNFRSF11A | Q9Y6Q6 | Tumor necrosis factor receptor superfamily member 11A | GeneCardss |
| 567 | TNFRSF11B | O00300 | Tumor necrosis factor receptor superfamily member 11B | GeneCardss |
| 568 | TNFRSF1A | P19438 | Tumor necrosis factor receptor superfamily member 1A | GeneCardss |
| 569 | TNFRSF1B | P20333 | Tumor necrosis factor receptor superfamily member 1B | GeneCardss |
| 570 | TNFSF11 | O14788 | Tumor necrosis factor ligand superfamily member 11 | GeneCardss |
| 571 | TNNI3 | P19429 | Troponin I, cardiac muscle | GeneCardss |
| 572 | TNNT2 | P45379 | Troponin T, cardiac muscle | GeneCardss |
| 573 | TP53 | P04637 | Cellular tumor antigen p53 | GeneCardss/GAD |
| 574 | TP63 | Q9H3D4 | Tumor protein 63 | GeneCardss |
| 575 | TPO | P07202 | Thyroid peroxidase | Drugbank/CTD/GeneCardss |
| 576 | TRH | P20396 | Pro-thyrotropin-releasing hormone | GeneCardss |
| 577 | TRHR | P34981 | Thyrotropin-releasing hormone receptor | GeneCardss |
| 578 | TSHB | P01222 | Thyrotropin subunit beta | CTD |
| 579 | TSHR | P16473 | Thyrotropin receptor | DisGeNET/CTD/GeneCardss/GAD |
| 580 | TTR | P02766 | Transthyretin | GeneCardss |
| 581 | TYR | P14679 | Tyrosinase | GeneCardss |
| 582 | VCAM1 | P19320 | Vascular cell adhesion protein 1 | GeneCardss |
| 583 | VDR | P11473 | Vitamin D3 receptor | CTD/GeneCardss/OMIM/GAD |
| 584 | VEGFA | P15692 | Vascular endothelial growth factor A | CTD/GeneCardss |
| 585 | VIM | P08670 | Vimentin | GeneCardss |
| 586 | VWF | P04275 | von Willebrand factor | GeneCardss |
| 587 | WFS1 | O76024 | Wolframin | OMIM |
| 588 | WNK1 | Q9H4A3 | Serine/threonine-protein kinase WNK1 | GeneCardss |
| 589 | XDH | P47989 | Xanthine dehydrogenase/oxidase [Includes: Xanthine dehydrogenase | GeneCardss |
| 590 | XRCC1 | P18887 | DNA repair protein XRCC1 | GeneCardss/GAD |
