# Supplementary material for: Diosgenin From Dioscorea Nipponica Rhizoma Against Graves’ Disease—On Network Pharmacology and Experimental Evaluation
Source: Front Pharmacol. 2022 Jan 24;12:806829. doi: 10.3389/fphar.2021.806829 (PMC8819592; doi:10.3389/fphar.2021.806829)
Supplement: Supplementary file 6 [file Table6.docx]

**Table S4** Information for overlapped targets after PPI analysis.

| Number | Name | Protein name | Degree | Closeness Centrality | Betweenness Centrality |
| --- | --- | --- | --- | --- | --- |
| 1 | ALB | Serum albumin | 60 | 0.82608696 | 0.15858583 |
| 2 | AKT1 | RAC-alpha serine/threonine-protein kinase | 54 | 0.77551020 | 0.08534334 |
| 3 | MAPK1 | Mitogen-activated protein kinase 1 | 50 | 0.73786408 | 0.07900465 |
| 4 | MMP9 | Matrix metalloproteinase-9 | 47 | 0.71698113 | 0.07312867 |
| 5 | EGFR | Epidermal growth factor receptor | 46 | 0.71028037 | 0.04716346 |
| 6 | SRC | Proto-oncogene tyrosine-protein kinase Src | 42 | 0.68468468 | 0.03528111 |
| 7 | IGF1 | Insulin-like growth factor I | 40 | 0.67256637 | 0.03432643 |
| 8 | ESR1 | Estrogen receptor | 36 | 0.64957265 | 0.03607985 |
| 9 | MAPK14 | Mitogen-activated protein kinase 14 | 32 | 0.62809917 | 0.01535094 |
| 10 | HSP90AA1 | Heat shock protein HSP 90-alpha | 32 | 0.63333333 | 0.02797533 |
| 11 | NOS3 | Nitric oxide synthase, endothelial | 31 | 0.62295082 | 0.01482605 |
| 12 | PIK3R1 | Phosphatidylinositol 3-kinase regulatory subunit alpha | 28 | 0.58914729 | 0.01391232 |
| 13 | IL2 | Interleukin-2 | 26 | 0.60317460 | 0.01243334 |
| 14 | PLG | Plasminogen | 26 | 0.59375000 | 0.01012545 |
| 15 | IGF1R | Insulin-like growth factor 1 receptor | 24 | 0.58914729 | 0.00531686 |
| 16 | BCL2L1 | Bcl-2-like protein 1 | 23 | 0.58461538 | 0.00344914 |
| 17 | REN | Renin | 22 | 0.57575758 | 0.01145800 |
| 18 | F2 | Prothrombin | 21 | 0.56716418 | 0.00794203 |
| 19 | NR3C1 | Glucocorticoid receptor | 21 | 0.57575758 | 0.00720724 |
| 20 | KIT | Mast/stem cell growth factor receptor Kit | 21 | 0.57575758 | 0.00845100 |
| 21 | PGR | Progesterone receptor | 21 | 0.57575758 | 0.00356888 |
| 22 | NOS2 | Nitric oxide synthase, inducible | 21 | 0.57575758 | 0.00664731 |
| 23 | ELANE | Neutrophil elastase | 21 | 0.56716418 | 0.01594292 |
| 24 | JAK2 | Tyrosine-protein kinase JAK2 | 21 | 0.57575758 | 0.00371813 |
| 25 | PPARG | Peroxisome proliferator-activated receptor gamma | 20 | 0.56296296 | 0.00793690 |
| 26 | SELE | E-selectin | 20 | 0.56296296 | 0.00520013 |
| 27 | SOD2 | Superoxide dismutase [Mn], mitochondrial | 20 | 0.57142857 | 0.00672830 |
| 28 | CCL5 | C-C motif chemokine 5 | 20 | 0.57142857 | 0.01426784 |
| 29 | MMP3 | Stromelysin-1 | 19 | 0.56296296 | 0.00282190 |
| 30 | AKT2 | RAC-beta serine/threonine-protein kinase | 19 | 0.56296296 | 0.00204708 |
| 31 | RAF1 | RAF proto-oncogene serine/threonine-protein kinase | 18 | 0.54676259 | 0.00232582 |
| 32 | PGF | Placenta growth factor | 18 | 0.55474453 | 0.00290911 |
| 33 | RXRA | Retinoic acid receptor RXR-alpha | 17 | 0.55072464 | 0.01303556 |
| 34 | FGF1 | Fibroblast growth factor 1 | 17 | 0.55072464 | 0.00129552 |
| 35 | SELP | P-selectin | 16 | 0.54676259 | 0.00338644 |
| 36 | MET | Hepatocyte growth factor receptor | 16 | 0.55474453 | 0.00097600 |
| 37 | CTSD | Cathepsin D | 14 | 0.54285714 | 0.00686439 |
| 38 | PARP1 | Poly [ADP-ribose] polymerase 1 | 14 | 0.53900709 | 0.00069400 |
| 39 | VDR | Vitamin D3 receptor | 14 | 0.53900709 | 0.00347233 |
| 40 | ADAM17 | Disintegrin and metalloproteinase domain-containing protein 17 | 13 | 0.53146853 | 0.00171384 |
| 41 | ERBB4 | Receptor tyrosine-protein kinase erbB-4 | 13 | 0.51700680 | 0.00169671 |
| 42 | HSPA8 | Heat shock cognate 71 kDa protein | 13 | 0.54285714 | 0.00654523 |
| 43 | RARA | Retinoic acid receptor alpha | 13 | 0.52054795 | 0.00202124 |
| 44 | TEK | Angiopoietin-1 receptor | 13 | 0.53146853 | 0.00083600 |
| 45 | MMP7 | Matrilysin | 12 | 0.53521127 | 0.00071300 |
| 46 | GSR | Glutathione reductase, mitochondrial | 12 | 0.52777778 | 0.00877101 |
| 47 | ESR2 | Estrogen receptor beta | 12 | 0.53521127 | 0.00011300 |
| 48 | TTR | Transthyretin | 12 | 0.52777778 | 0.00525085 |
| 49 | LPA | Apolipoprotein | 12 | 0.51351351 | 0.00161168 |
| 50 | MME | Neprilysin | 11 | 0.52413793 | 0.00199266 |
| 51 | RAC2 | Ras-related C3 botulinum toxin substrate 2 | 11 | 0.50000000 | 0.00128791 |
| 52 | PIK3CG | Phosphatidylinositol 4,5-bisphosphate 3-kinase catalytic subunit gamma isoform | 11 | 0.50000000 | 0.00040200 |
| 53 | LYZ | Lysozyme C | 11 | 0.52413793 | 0.00342418 |
| 54 | MMP8 | Neutrophil collagenase | 10 | 0.52054795 | 0.00106822 |
| 55 | LGALS3 | Galectin-3 | 10 | 0.52054795 | 0.00052000 |
| 56 | PPARA | Peroxisome proliferator-activated receptor alpha | 10 | 0.52777778 | 0.00084100 |
| 57 | PLAT | Tissue-type plasminogen activator | 10 | 0.51700680 | 0.00026400 |
| 58 | GC | Vitamin D-binding protein | 10 | 0.50666667 | 0.00325295 |
| 59 | MIF | Macrophage migration inhibitory factor | 9 | 0.52413793 | 0.00112004 |
| 60 | RARB | Retinoic acid receptor beta | 9 | 0.50000000 | 0.00136341 |
| 61 | DPP4 | Dipeptidyl peptidase 4 | 9 | 0.51351351 | 0.00050200 |
| 62 | GSTP1 | Glutathione S-transferase P | 9 | 0.52054795 | 0.00225986 |
| 63 | THRA | Thyroid hormone receptor alpha | 9 | 0.47798742 | 0.00089900 |
| 64 | THRB | Thyroid hormone receptor beta | 9 | 0.49350649 | 0.00157972 |
| 65 | LTF | Lactotransferrin | 8 | 0.48717949 | 0.00087500 |
| 66 | TGFBR1 | TGF-beta receptor type-1 | 8 | 0.51700680 | 0.00013200 |
| 67 | ITGAL | Integrin alpha-L | 8 | 0.50331126 | 0.00093700 |
| 68 | HPRT1 | Hypoxanthine-guanine phosphoribosyltransferase | 6 | 0.50331126 | 0.02073747 |
| 69 | S100A9 | Protein S100-A9 | 6 | 0.47798742 | 0.00026300 |
| 70 | AHCY | Adenosylhomocysteinase | 4 | 0.41081081 | 0.00534086 |
| 71 | GSTM1 | Glutathione S-transferase Mu 1 | 4 | 0.47204969 | 0.00000000 |
| 72 | HEXB | Beta-hexosaminidase subunit beta | 4 | 0.46341463 | 0.00000000 |
| 73 | HSD11B1 | Corticosteroid 11-beta-dehydrogenase isozyme 1 | 4 | 0.43428571 | 0.00003190 |
| 74 | CA2 | Carbonic anhydrase 2 | 3 | 0.46625767 | 0.00005580 |
| 75 | PSAP | Prosaposin | 3 | 0.43428571 | 0.00009360 |
| 76 | NR1H2 | Oxysterols receptor LXR-beta | 3 | 0.45783133 | 0.00009400 |
| 77 | PNP | Purine nucleoside phosphorylase | 2 | 0.34389140 | 0.00036700 |
